# Synthesis, physicochemical characterization and neuroprotective evaluation of novel 1-hydroxypyrazin-2(1*H*)-one iron chelators in an *in vitro* cell model of Parkinson's disease†

**DOI:** 10.1039/d1dt02604f

**Published:** 2022-02-11

**Authors:** Frank W. Lewis, Kathleen Bird, Jean-Philippe Navarro, Rawa El Fallah, Jeremy Brandel, Véronique Hubscher-Bruder, Andrew Tsatsanis, James A. Duce, David Tétard, Samuel Bourne, Mahmoud Maina, Ilse S. Pienaar

**Affiliations:** Department of Applied Sciences, Faculty of Health and Life Sciences, Northumbria University Newcastle upon Tyne Tyne and Wear NE1 8ST UK frank.lewis@northumbria.ac.uk +44 (0)0191 227 3519 +44 (0)191 227 4637; Université de Strasbourg, CNRS, IPHC UMR 7178 F-67000 Strasbourg France jbrandel@unistra.fr +33 (0)368 852 749; School of Biomedical Sciences, The Faculty of Biological Sciences, University of Leeds Leeds West Yorkshire LS2 9JT UK; Alzheimer's Research UK Cambridge Drug Discovery Institute, Cambridge Bio-medical Campus, University of Cambridge Cambridge UK jad205@cam.ac.uk +44 (0)1223 335 656; School of Life Sciences, University of Sussex Falmer Sussex BN1 9PH UK I.S.Pienaar@sussex.ac.uk +44 (0)1273 678 057; Institute of Clinical Sciences, University of Birmingham Edgbaston Birmingham B15 2TT UK

## Abstract

Iron dysregulation, dopamine depletion, cellular oxidative stress and α-synuclein protein mis-folding are key neuronal pathological features seen in the progression of Parkinson's disease. Iron chelators endowed with one or more therapeutic modes of action have long been suggested as disease modifying therapies for its treatment. In this study, novel 1-hydroxypyrazin-2(1*H*)-one iron chelators were synthesized and their physicochemical properties, iron chelation abilities, antioxidant capacities and neuroprotective effects in a cell culture model of Parkinson's disease were evaluated. Physicochemical properties (log *β*, log *D*_7.4_, pL_0.5_) suggest that these ligands have a poorer ability to penetrate cell membranes and form weaker iron complexes than the closely related 1-hydroxypyridin-2(1*H*)-ones. Despite this, we show that levels of neuroprotection provided by these ligands against the catecholaminergic neurotoxin 6-hydroxydopamine *in vitro* were comparable to those seen previously with the 1-hydroxypyridin-2(1*H*)-ones and the clinically used iron chelator Deferiprone, with two of the ligands restoring cell viability to ≥89% compared to controls. Two of the ligands were endowed with additional phenol moieties in an attempt to derive multifunctional chelators with dual iron chelation/antioxidant activity. However, levels of neuroprotection with these ligands were no greater than ligands lacking this moiety, suggesting the neuroprotective properties of these ligands are due primarily to chelation and passivation of intracellular labile iron, preventing the generation of free radicals and reactive oxygen species that otherwise lead to the neuronal cell death seen in Parkinson's disease.

## Abstract 2

We report that novel 1-hydroxypyazin-2(1*H*)-ones show comparable neuroprotective effects to related iron chelators in a cell culture model of Parkinson's disease, despite significant differences in their physicochemical properties.

## Graphical abstract

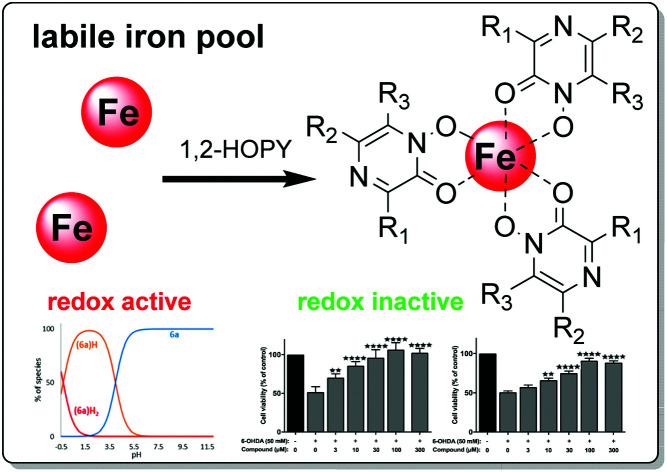

## Introduction

Parkinson's disease (PD) is a debilitating neurodegenerative disorder in which patients experience progressive loss of motor control, revealing symptoms such as bradykinesia, tremor and rigidity.^1^ Such clinical features are due to the progressive and selective degeneration of neuromelanin-containing midbrain neurons, in particular the dopaminergic neurons in the Substantia Nigra (SN) midbrain region. Although the exact cause of idiopathic PD is as yet unknown, one of the pathological hallmarks of PD is dysregulation of intracellular and extracellular iron levels, and there is strong evidence indicating that localised iron accumulation occurs in the SN of PD patients.^2^ In addition, many neurotoxins used as models of PD such as 6-hydroxydopamine (6-OHDA),^3^ 1-methyl-4-phenyl-1,2,3,6-tetrahydropyridine (MPTP),^4^ lactacystin,^5^ rotenone^6^ or paraquat^4*b*^ lead to an accumulation of intracellular labile iron in addition to other PD pathologies such as disruption of catecholaminergic neurotransmitters, α-synuclein protein mis-folding and aggregation, and oxidative damage that eventually leads to neuronal death.^7^ Moreover, implication of ferroptosis in PD is gaining increasing interest, indicating a central role played by iron accumulation in neuronal death.^8^

When weakly bound or unbound by intracellular chelators, labile iron is redox active and can cycle between its ferrous (Fe^2+^) and ferric (Fe^3+^) ion forms, which leads to excessive production of free radicals and reactive oxygen species (ROS) through the Haber–Weiss reactions.^9^ Such an accumulation of free radicals and ROS can eventually overwhelm cellular antioxidant defences such as glutathione, causing the cellular damage that is characteristic of PD such as lipid and protein oxidation,^10^ DNA damage^11^ and iron-induced α-synuclein mis-folding and aggregation.^12^ This redox cycling of iron is avoided in the healthy brain, where iron is strongly chelated by iron transport and storage proteins (ferroportin and ferritin, respectively) and labile iron levels are tightly controlled.^13^

**Fig. 1:**
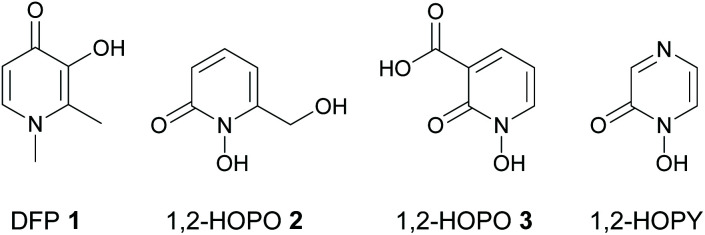
Structures of deferiprone (DFP) 1, and 1-hydroxypyridin-2(1*H*)-ones 2 and 3.

Due to the crucial role that iron dysregulation plays in the progression of PD, iron chelators have long been proposed as potential disease-modifying therapies for its treatment.^14^ Indeed, clinical iron chelators such as desferrioxamine (DFO) and deferiprone (DFP 1, Fig. 1) have been successfully used in other therapeutic areas such as the iron overload diseases thalassemia and sickle cell anemia,^15^ while other metal chelators such as 8-hydroxyquinolines^16^ and aroylhydrazones^17^ have been proposed for the treatment of both PD and Alzheimer's disease; another neurodegenerative disorder involving metal dysregulation.^18^ Furthermore, deferiprone 1 showed promising results in recent phase II clinical trials for treatment of PD.^19^

Any iron chelator used clinically for the treatment of PD must fulfil a specific set of criteria. It should be capable of stabilizing iron in its ferric (Fe^3+^) form over its ferrous (Fe^2+^) form such that redox cycling between the two forms does not occur. It should be strong enough to chelate and passivate labile iron but not so strong as to remove iron from iron transport and storage proteins or inhibit iron-containing enzymes such as tyrosine hydroxylase; an essential enzyme involved in the biosynthesis of dopamine.^20^ In addition, it should have a relatively low molecular weight and an optimum lipophilicity in line with the Lipinski parameters^21^ for orally active drugs to ensure it is reasonably capable of penetrating cell membranes and the blood–brain barrier (BBB).‡‡For example, DFO, a chelator with a high MW/large size, high hydrophilicity and very high iron affinity (log *β* = 30.5) provides only limited benefit at large doses in animal models of PD and has to be directly administered to the brain to be therapeutically effective. See: Ben-Shachar *et al.* (1992) and Dexter *et al.* (2011)^57*a*,*b*^.

As Fe^3+^ is classified as a hard Lewis acid according to Pearson's HSAB theory,^22^ hard Lewis bases are preferred in order to chelate iron and stabilize it in its ferric form as Fe^3+^ over its ferrous form as Fe^2+^. This suggests that bidentate O-donor ligands such as hydroxypyridinones (HOPOs)^23^ are particularly attractive candidates for further development as iron chelator drugs for PD therapy. However, although HOPOs fulfil many of the above criteria and are widely used as bidentate metal binding groups in many siderophore mimics,^24,25^ most research on their therapeutic use against PD has focused on 3-hydroxypyridin-4(1*H*)-ones (3,4-HOPOs) such as DFP 1 and its derivatives.^26^ We previously showed that 1-hydroxypyridin-2(1*H*)-ones (1,2-HOPOs) such as 2 and 3 (Fig. 1) are also promising iron chelators in cell culture models of PD *in vitro*.^27,28^ Compounds 2 and 3 reduced intracellular labile iron levels in both the 6-OHDA^27^ and lactacystin^28^ models of PD, and restored labile iron and iron-responsive protein expression to normal levels in the 6-OHDA model of PD.^27^ More recently, we showed that both compounds also reduced α-synuclein accumulation induced by the potent ubiquitin proteasomal inhibitor lactacystin.^28^

A closely related family of chelators to the 1,2-HOPOs are the 1-hydroxypyrazin-2(1*H*)-ones (1,2-HOPYs, Fig. 1). These chelators have been reported as bidentate iron binding groups in siderophore mimics,^29^ and have found use in other applications such as coupling reagents in peptide synthesis^30^ and as ligands in luminescent lanthanide complexes.^31^ However, currently there are no reports on the study of 1,2-HOPYs as potential iron chelators for the treatment of PD. We reasoned that 1,2-HOPYs could be particularly attractive candidates in this regard for two reasons. Firstly, the additional N-atom present in 1,2-HOPYs compared to 1,2-HOPOs renders these molecules more hydrophilic, which could lead to better oral bioavailability with minimal impact on BBB penetration.^32^ Secondly, these molecules are readily synthesized by condensation reactions of amino acid-derived hydroxamic acids with different α-dicarbonyl compounds.^33^ Thus, there is a wide scope to fine tune the physicochemical properties of the chelators (*e.g.*: partition coefficients, p*K*_a_ values, molecular weights, *etc.*) through choice of appropriate starting materials. Herein we report the synthesis, physicochemical evaluation and iron chelating properties of some novel 1,2-HOPYs, as well as their neuroprotective potential in an *in vitro* cell culture model of PD.

## Results and discussion

### Organic synthesis

**Scheme 1:**
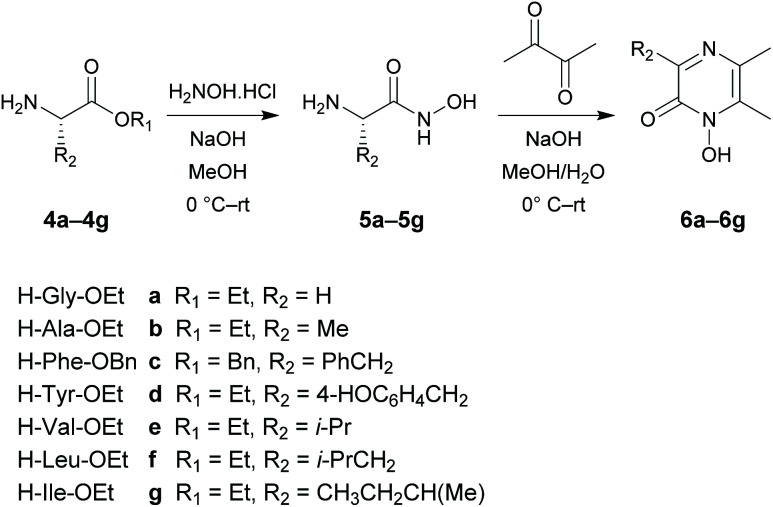
Synthesis of 1-hydroxypyrazin-2(1*H*)-ones 6a–6g.

**Scheme 2:**
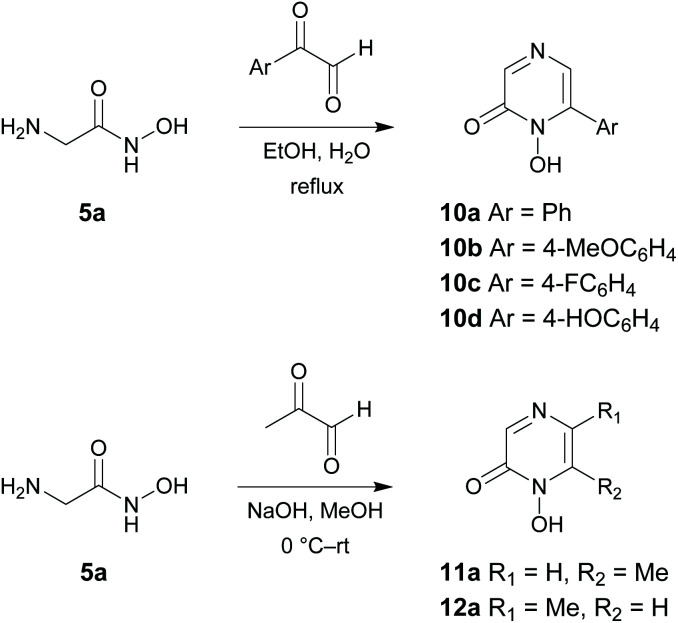
Synthesis of 1-hydroxypyrazin-2(1*H*)-ones 10a–10d, 11a and 12a.

The target 1-hydroxypyrazin-2(1*H*)-ones 6, 10, 11 and 12 were synthesized in two steps from amino acid ethyl esters following literature procedures^29–31,33–39^ as shown below in Schemes 1 and 2. It is known that multifunctional hydroxypyridinone metal chelators containing phenolic antioxidant moieties show promising efficacy against neurodegenerative diseases by acting as radical traps as well as metal chelators.^40^ Therefore, we also synthesized 1-hydroxypyrazin-2(1*H*)-ones 6d and 10d which each contain a phenol moiety which could provide a beneficial antioxidant mode of action in addition to iron chelation. A full discussion of the synthesis of 6, 10, 11 and 12 is given in the ESI.†

### Ligand p*K*_a_ values and complex stability constants

Physicochemical measurements were then carried out on the 1-hydroxypyrazin-2(1*H*)-ones to evaluate their acid–base behaviour and Fe^3+^ complexation properties with respect to pH. Acid–base properties of ligands 6a, 6c, 6d, 10a and 11a were determined by direct UV-visible spectrophotometric titrations *vs*. pH between pH 2 and 12 and batch titration between p[H] −0.36 and 2. For solubility reasons, ligands 6a and 11a and their Fe^3+^ complexes were studied in water while ligands 6c and 6d were studied in a mixed MeOH/H_2_O (80/20 w/w) solvent. For ligand 10a, protonation constants of the ligand were studied in both media while complexation studies were carried out only in the mixed solvent for solubility reasons.

**Fig. 2:**
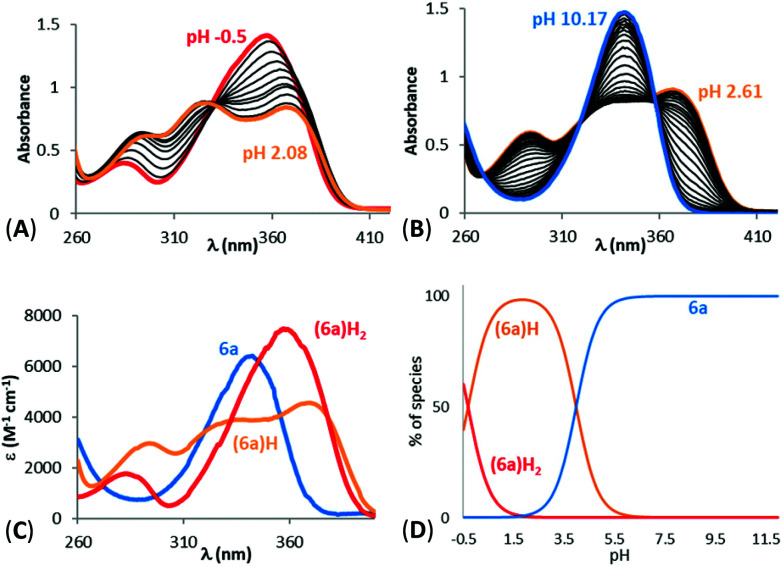
Spectrophotometric titrations *vs.* pH of ligand 6a between (A) −0.5 < pH < 2.08 (batch titration, [6a] = 2.57 × 10^−4^ M) and (B) 2.61 < pH < 10.17 (direct titration, [6a] = 2.55 × 10^−4^ M). (C) Electronic spectra and (D) distribution curves ([6a] = 1.54 × 10^−4^ M) of the protonated species of ligand 6a. Solvent: H_2_O, *I* = 0.1 M (NaClO_4_), *T* = 25.0 °C.

The titrations of the five ligands (6a in Fig. 2A and B, 6c, 6d, 10a and 11a in Fig. S1(A,B)–S5(A,B)†) showed the presence of one protonation equilibrium in the 2 to 12 pH range and a second one in the −0.36 to 2 p[H] range for each ligand. Analysis of the spectral variations^41^ allowed us to suggest the stoichiometry of the species formed and to calculate their protonation constants (Table 1). From these protonation constants the electronic spectra and distribution curves of the species *vs*. pH were calculated (6a in Fig. 2C and D, 6c, 6d, 10a and 11a in Fig. S1(C,D)–S5(C,D)†).

**Table 1:** Protonation constants (log *K*) of ligands, stability constants (log *β*) of their Fe^3+^ complexes, pL_0.5_ values, distribution coefficients and C log *P* values of ligands^a^

Ligand	6a	11a	10a	10a	6d	6c
Solvent	H_2_O	H_2_O	H_2_O	MeOH/H_2_O	MeOH/H_2_O	MeOH/H_2_O
log *K* LH	4.58(4)	3.98(5)	3.18(1)	4.24(2)	10.57(1)	5.53(2)
log *K* LH_2_	0.04(2)	0.2(2)	∼−0.6	∼−0.9	5.96(1)	0.66(1)
log *K* LH_3_	—	—	—	—	0.94(1)	—
log *β* FeL	10.38(6)	7.83(1)	—	9.79(2)	—	12.9(1)
log *β* FeLH	—	—	—	—	26.7(1)	—
log *β* FeL_2_	18.1(4)	14.28(2)	—	17.89(2)	—	21.9(1)
log *β* FeL_2_H_2_	—	—	—	—	46.3(1)	—
log *β* FeL_3_	23.85(1)	20.19(4)	—	21.7(1)	—	27.8(1)
log *β* FeL_3_H_3_	—	—	—	—	62.3(1)	—
pL_0.5_	4.36	3.13	—	3.95	7.34	5.98
log D_7.4_^b^	−1.73(6)	−0.4(1)	−1.2(2)	—	—	—
C log *P*^c^	−0.13	−0.75	0.84	0.84	1.56	2.12
BBB score^d^	3.88	3.97	4.47	4.47	4.05	4.70

a Hydroxide formation constants (log *β*) were Fe(OH) = −2.56, Fe(OH)_2_ = −6.20, Fe(OH)_3_ = −11.41, Fe(OH)_4_ = −21.88, Fe_2_(OH)_2_ = −2.84 and Fe_3_(OH)_4_ = −6.05 in H_2_O [see ref. 54] and Fe(OH) = −1.57, Fe(OH)_2_ = −4.63 in MeOH/H_2_O (80/20).

b Measured in HEPES buffered aqueous phase.

c Calculated using ACD-I/Lab.

d Obtained from ref. 32. Structural parameters used for BBB score calculations were obtained using SwissADME (http://www.swissadme.ch/).

The distribution curves suggested that, at physiological pH (pH 7.4), the five studied ligands are negatively charged with the *N*-hydroxyl group of the 1-hydroxypyrazin-2(1*H*)-one ring being deprotonated. The phenolic proton of ligand 6d remains protonated. The study of ligand 10a in both H_2_O and MeOH/H_2_O (80/20 w/w) solvents showed an increase of the first protonation constant of around 1 order of magnitude in the mixed solvent. As expected, the protonation constants of the 1-hydroxypyrazin-2(1*H*)-ones corresponding to loss of the N–OH proton are lower than those of the related 1-hydroxypyridin-2(1*H*)-ones 2 and 3^28^ due to the added stabilisation of the conjugate base by the additional N-atom in the pyrazine ring of 1-hydroxypyrazin-2(1*H*)-ones. The protonation constant for 6a is also in excellent agreement with those published previously for this compound (p*K*_a_ = 4.7) and the corresponding unsubstituted 1-hydroxypyrazin-2(1*H*)-one (p*K*_a_ = 4.4).^35^ The low protonation constants suggest these molecules will have a relatively poor ability to cross cell membranes by passive diffusion.

**Fig. 3:**
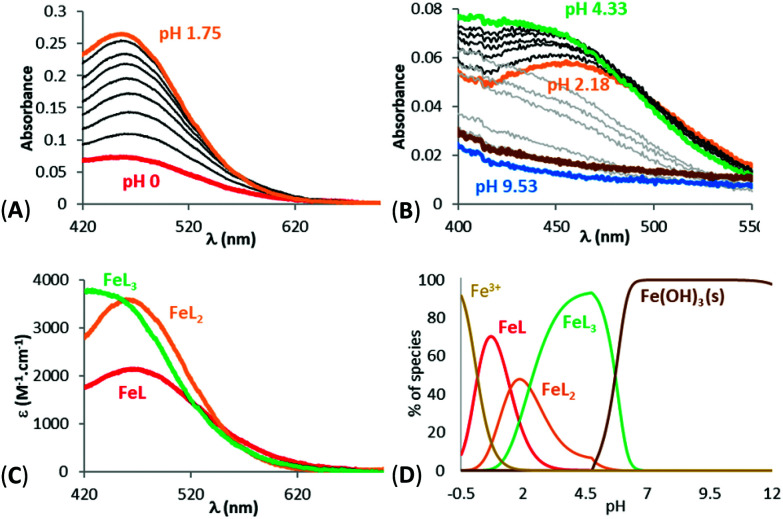
Spectrophotometric titration *vs.* pH of Fe^3+^ complexes of ligand 11a between (A) 0 ≤ pH ≤ 1.75 (batch titration, [11a] = 2.55 × 10^−4^ M, [Fe^3+^] = 7.88 × 10^−5^ M) and (B) 2.18 ≤ pH ≤ 11.48 (direct titration, [11a] = 1.02 × 10^−4^ M, [Fe^3+^] = 3.12 × 10^−5^ M). (C) Electronic spectra and (D) distribution curves ([11a] = 2.55 × 10^−4^ M, [Fe^3+^] = 8.04 × 10^−5^ M) of the Fe^3+^ complexes of 11a. Solvent: H_2_O, *I* = 0.1 M (NaClO_4_), *T* = 25.0 °C.

Fe^3+^ complexation studies *vs.* pH were carried out in the same way, *via* spectrophotometric batch titrations between p[H] −0.36 and 2 and *via* direct titrations between pH 2 and 12. The spectra of Fe^3+^ complexation titrations showed the appearance of a ligand to metal charge transfer (LMCT) band between 450 and 650 nm for all the studied ligands (11a in Fig. 3A and B, 6a, 6c, 6d, 10a in Fig. S6(A,B)–S9(A,B)†); a sign that complexation started at very low pH. The LMCT band underwent a hyperchromic and hypsochromic shift with increasing pH suggesting an increase of the number of ligands in the coordination sphere of Fe^3+^. The LMCT band then decreased together with an increase of the baseline, indicating the progressive decomposition of the complexes and precipitation of iron hydroxides (Fe(OH)_3_).

Analysis of the spectral variations prior to precipitation suggested the successive formation of FeL, FeL_2_ and FeL_3_ complexes for L = 6a, 6c, 10a, 11a, and FeLH, FeL_2_H_2_ and FeL_3_H_3_ complexes for ligand 6d as precipitation occurred before deprotonation of the phenolic proton. The calculated stability constants (log *β*) of the species are reported in Table 1. From the values of these stability constants, the electronic spectra of the complex species (11a in Fig. 3C, 6a, 6c, 6d, 10a in Fig. S6C–S9C†) and their distribution curves (11a in Fig. 3D, 6a, 6c, 6d, 10a in Fig. S6D–S9D†) were calculated. The stability of the 1 : 3 Fe^3+^ complex of 6a is in reasonable agreement with that reported previously under different experimental conditions (log *β*_3_ = 20.2).^35^

As the complexation of a metal by a ligand is dependent, among other parameters (metal ion concentration, ionic strength, ionic medium, pH, temperature), on the protonation constants of the ligands, the stability constants of the complexes cannot be used as such to compare the sequestering ability of a series of ligands for a given metal. This sequestering power can be evaluated by the determination of an empirical and quantitative parameter; pL_0.5_, which represents the total concentration of ligand required for the sequestration of 50% of the metal.^42^

**Fig. 4:**
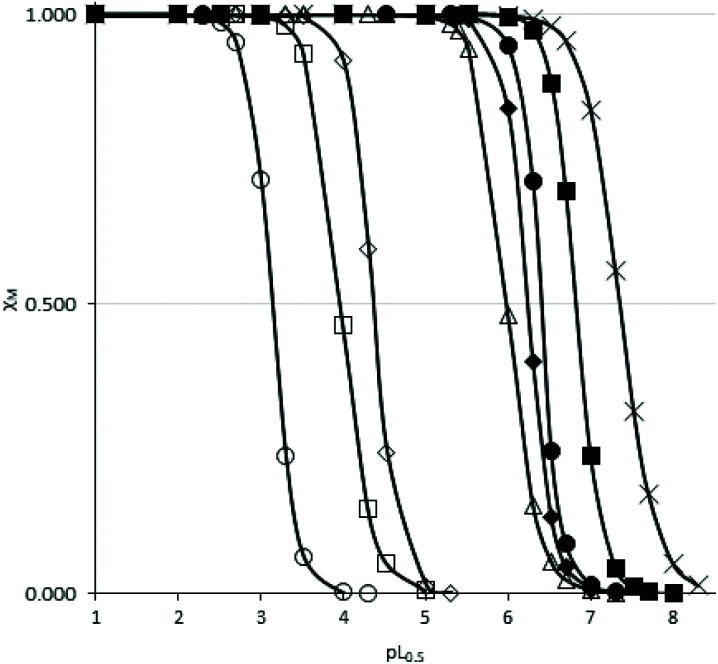
Sequestration diagrams towards Fe^3+^ at pH = 7.4 of ligands 11a (O), 10a (□), 6a (◇), 6c (Δ), 6d (×) and previously studied ligands 2 (◆), 3 (●) and DFP 1 (■). [Fe^3+^] = 10^−9^ M, *T* = 25.0 °C, *I* = 0.1 M (NaClO_4_).

It can be calculated from a sigmoidal Boltzmann-type equation:
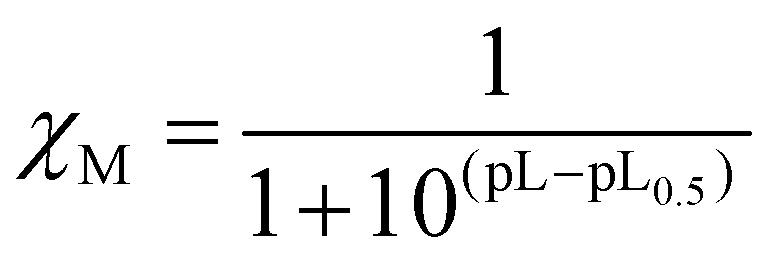
where χ_M_ = mole fraction of metal cation complexed by the ligand, pL = −log *c*_L_ and pL_0.5_ = −log *c*_L_, if *χ*_M_ = 0.5. According to this equation, the higher the pL_0.5_ value, the higher the sequestering ability of the ligand. The sequestering ability of ligands 6a, 6c, 6d, 10a, 11a, as well as ligands 2, 3 and DFP 1 towards Fe^3+^ at pH 7.4 are presented in Fig. 4, and the pL_0.5_ values are reported in Table 1.

The pL_0.5_ values suggested the following order of sequestering ability at pH 7.4: 6d > 6c > 6a > 10a > 11a. These values suggested that ligands 6c and 6d are the strongest chelators of the 1-hydroxypyrazin-2(1*H*)-ones series at this pH, which can be explained by the higher p*K*_a_ values of these ligands. It also suggests that ligand 6d might be a stronger Fe^3+^ chelator than DFP 1. However, ligand 6d (as well as 6c and 10a) were studied in MeOH/H_2_O (80/20 w/w) for solubility reasons, so the chelation power in water would likely be lower, as suggested by the p*K*_a_ value of 10a which is 1 order of magnitude lower in H_2_O than in MeOH/H_2_O (80/20 w/w). The chelation powers of 1-hydroxypyrazin-2(1*H*)-ones 6a and 11a in water are slightly lower than those of 1-hydroxypyridin-2(1*H*)-ones 2 and 3,^28^ and the order of chelation power of the water-soluble species at pH 7.4 is thus DFP 1 > 1-hydroxypyridin-2(1*H*)-ones > 1-hydroxypyrazin-2(1*H*)-ones.

### Distribution coefficients

The distribution coefficients (log *D*_7.4_) between an n-octanol phase and a HEPES-buffered aqueous phase at pH 7.4 were determined for ligands 6a, 10a and 11a (Table 1) and Fe^3+^ complexes of ligands 6a (log *D*_7.4_ = 0.9(1)) and 11a (log *D*_7.4_ = 0.1(1)). Log *D*_7.4_ values of ligands 6c and 6d and their Fe^3+^ complexes could not be determined due to their low solubilities in the aqueous phase. The partition coefficients of 1-hydroxypyrazin-2(1*H*)-ones are very similar to those of the 1-hydroxypyridin-2(1*H*)-ones reported previously (*e.g.*: log *D*_7.4_ of 6a = −1.73, log *D*_7.4_ of 2 = −1.71).^28^ Although this suggests a relatively poor ability of 1-hydroxypyrazin-2(1*H*)-ones to penetrate cell membranes by passive diffusion, the possibility that they can penetrate cell membranes by active transport mechanisms cannot be discounted. In addition, the distribution coefficients of the Fe^3+^ complexes of 6a and 11a are higher than that of 2 (log *D*_7.4_ = −0.18). This suggests that the Fe^3+^ complexes of 1-hydroxypyrazin-2(1*H*)-ones can penetrate cell membranes more easily than the free ligands, which could enable therapeutic re-distribution of labile Fe^3+^ to take place between neurons within the brain more easily. The predicted BBB penetration scores of the compounds are indicated in Table 1.^32^ A score between 4 and 5 indicates that the compounds have a 54.5% statistical ability to penetrate the BBB. Compounds 6c (BBB score = 4.70), 6d (BBB score = 4.05) and 10a (BBB score = 4.47) are therefore predicted to have good prospects for penetrating the BBB and reaching the central nervous system.

### DPPH radical scavenging assay

DPPH˙ free radical scavenging assays were carried out on ligands 6a, 6c, 6d, 10a and 11a at pH 7.4 (Fig. S11–S15†). This assay, as well as the Trolox Equivalent Antioxidant Capacity (TEAC) assay, should not be considered to reflect the actual antioxidant ability of the ligands *in vivo*, as it is too simplistic compared to the complexity of an *in vivo* environment. Nevertheless, they are a useful way to compare the relationship between structure and antioxidant properties in this series of ligands.

The kinetics of reaction suggested that these ligands can be classified as being slow radical scavengers, just like the 1-hydroxypyridin-2(1*H*)-ones 2 and 3.^28,43^ The calculated EC_50_ values (Table 2) showed that ligands 6a, 6c, 6d and 11a have a similar radical scavenging capability, while ligand 10a exhibited a 4 to 5 times lower EC_50_ value, which is slightly better than the commercial ligand DFP 1 (EC_50_ = 0.008 mg mL^−1^ = 5.8 × 10^−5^ M) and comparable to multifunctional metal chelators containing phenol antioxidant moieties.^44^ Somewhat surprisingly, ligand 6d was no better than the other ligands in this assay despite its added phenol moiety. The higher radical scavenging ability of 10a compared to 6c and 6d in this assay could be due to the phenyl ring in 10a being conjugated to the 1-hydroxypyrazin-2(1*H*)-one ring. Addition of free radicals such as the hydroxyl radical to the phenyl ring of 10a would therefore generate a more heavily delocalised radical compared to those generated by radical addition to the phenyl ring of 6c, or hydrogen atom abstraction from the phenol ring of 6d.

**Table 2:** EC_50_ values from the DPPH radical scavenging assay

Ligand	6a	11a	10a	6d	6c
EC_50_ (mg mL^−1^)	0.020	0.020	0.005	0.029	0.027
M (g mol^−1^)	140.06	126.04	188.06	246.26	230.26
EC_50_ (mol L^−1^)	1.4 × 10^−4^	1.6 × 10^−4^	2.8 × 10^−5^	1.2 × 10^−4^	1.2 × 10^−4^

### Trolox equivalent antioxidant capacity (TEAC) assay

The antioxidant capacities of ligands 6a, 6c, 6d, 10a and 11a were also investigated using a TEAC assay.^45^ 2,2′-Azinobis-(3-ethylbenzothiazoline)-6-sulfonic acid radical cation (ABTS˙^+^) is a blue/green chromophore that absorbs at 745 nm and was produced through the reaction between ABTS and potassium persulfate. Addition of antioxidants to the radical cation reduces it to the non-absorbing ABTS to an extent and on a time-scale depending on its antioxidant activity, the concentration of the antioxidant and the duration of the reaction. The extent of inhibition of the absorbance of ABTS˙^+^ at 745 nm was plotted as a function of the concentration of ligands 6a, 6c, 6d, 10a and 11a at different time points (*t* = 1, 4 and 6 minutes, Fig. S16–S20†). The TEAC value for a given ligand at a given time point was obtained by dividing the corresponding slope of the ligand by the slope of Trolox (Table 3 and Fig. S21†).

**Table 3:** Antioxidant activity as TEAC (mM) at specific time-points

Ligand	6a	11a	10a	6d	6c	DFP 1
*t* = 1 min	0.08	0.02	0.66	0.41	0.21	∼0.7
*t* = 3 min	0.16	0.03	0.69	0.55	0.31	∼0.7
*t* = 6 min	0.22	0.05	0.70	0.64	0.40	∼0.7

The results suggest that ligand 10a was the only one to have an essentially complete reaction after 3 minutes. In comparison, DFP 1 had completely reacted within 1 minute. It can be observed that ligand 11a had less effect on the quenching of ABTS˙^+^, followed by ligand 6a. The quenching ability therefore seems to increase with the addition of a benzyl substituent in the 3-position in ligand 6c and a *p*-hydroxybenzyl substituent in the 3-position in ligand 6d. In agreement with the results from the DPPH assay, the highest radical scavenging capacity is observed for ligand 10a, suggesting that the spacer between the phenyl ring and the 1-hydroxypyrazin-2(1*H*)-one ring (CH_2_ spacer in 6c and 6d v no spacer in 10a) and/or its position (3-position in 6c and 6d v 6-position in 10a) plays an important role in the antioxidant properties of these ligands. Thus, the addition of a phenyl ring to these ligands is beneficial for antioxidant activity in this assay, especially if it is conjugated to the 1-hydroxypyrazin-2(1*H*)-one ring as in 10a.

### Novel 1-hydroxypyrazin-2(1*H*)-one derivatives offer neuroprotection against PD-relevant neurotoxicity

**Fig. 5:**
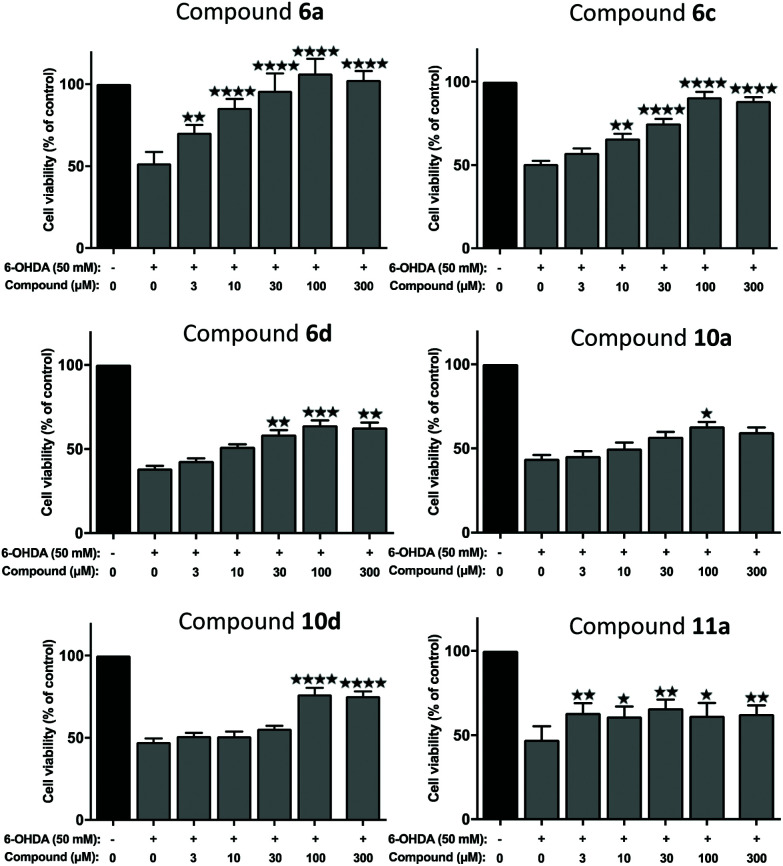
Assessment of neuroprotection offered against the PD-relevant neurotoxin, 6-OHDA. All novel 1,2-HOPY chelators offered at least partial neuroprotection against 6-OHDA. The degree of protection varied between the compounds, depending, at least in part, whether the compound structurally included a phenol moiety and extra carbon linker between the benzene and pyrazine rings. As measured *via* a well-established cell viability assay, incubation with 6-OHDA decreased cell viability by approximately 50%. However, pre-incubation with 6a fully rescued SH-SY5Y cells against 6-OHDA-induced cell death, particularly at higher concentrations. Co-incubation with 6c and 10d also partially prevented SH-SY5Y-mediated cell death, where this was most prevalent at higher concentrations. 10a offered only marginal protection, reaching statistical significance at a single dose. Cell viability was calculated as a percentage, relative to untreated (no toxin/compound) controls. Levels of significance (indicated as the number of stars: ****P ≤ 0.0001, ***P ≤ 0.001, **P ≤ 0.01, *P ≤ 0.05) is shown, in reference to toxin-only treated cells. All data is shown as the mean ± standard error mean (SEM) of three independent experiments. One-way ANOVA followed by a Dunnett's *post-hoc* test.

We next evaluated the neuroprotective ability of these compounds when cells are exposed to 6-hydroxydopamine (6-OHDA), a neurotoxin that emulates various pathological aspects of PD in dopaminergic neurons, in both *in vitro* and *in vivo* disease models, including mitochondrial impairment and cell death.^26*c*,28,46^ We evaluated the potential protective effects offered by 6a, 11a, 6c, 6d, 10a and 10d against 6-OHDA-induced cell injury in SH-SY5Y neuroblastoma cells, a catecholaminergic cell line that is used routinely for screening potential anti-PD therapeutic agents.^28,47^ Compounds 10b and 10c were not evaluated due to their low water solubilities. Cells were pre-treated with the compound of interest at different concentrations for 1 hour prior to treatment with 6-OHDA (50 mM) for a further 24 hours. The results of the cell viability assay for compounds 6a, 6c, 6d, 10a, 10d and 11a are shown in Fig. 5.

Considerable variation was noted in terms of the different compounds’ anti-toxin neuroprotective capacity. Compound 6a showed the highest degree of neuroprotection, and restored cell viability to ∼90% and 100% when cells were pre-treated with doses of 30 μM and 100 μM, respectively. This result was somewhat surprising, given the low partition coefficient of 6a, compared to the other compounds tested here (*cf.* log *D*_7.4_ values, Table 1), suggesting that 6a has a poor ability to penetrate cell membranes *via* passive diffusion. In contrast, treatment with compound 11a, at a 100 μM dosage, restored cell viability by merely ∼60%, relative to non-toxin control cells, despite having a higher partition coefficient than 6a. Pre-application of 6c also provided dose-related neuroprotection against the toxin treatment, which peaked at the 100 μM treatment dosage (by restoring cell viability to ∼89%, compared to toxin-treated cells; ****P < 0.0001). At this dosage, 10d, 10a and 6d also afforded significant neuroprotection against toxin-induced cell injury by restoring mean cell viability levels to 76.48%, 63.09% and 64.06%, respectively. It was noted that the highest drug concentration tested here (300 μM), offered no additional neuroprotective benefit over treatment at the 100 μM dose, for any of the compounds tested.

In order to chelate and passivate intracellular labile iron, the ligands must first penetrate cellular plasma membranes to gain entry to the cell. We therefore also calculated the partition coefficients (C log *P*) of the ligands to shed light on their hydrophobicities (Table 1). Although these values should always be interpreted with care, this property can be useful for predicting the membrane permeability of drugs.^21^ Examination of these values suggest there is no clear discernible relationship between the calculated hydrophobicities of the ligands and their neuroprotective properties, except between ligands that are closely related structurally. Thus, 6a is more neuroprotective than 11a, in line with its greater hydrophobicity than 11a. Similarly, 6c is more neuroprotective than 6d, in line with its greater hydrophobicity compared to 6d. On the other hand, 10d is more neuroprotective than 10a at higher doses despite being more hydrophilic than 10a. However, this could be due to the additional antioxidant effects conferred by the phenol moiety in 10d, in addition to iron chelation and passivation. As mentioned earlier, it is possible that the ligands could gain entry to the cell by active transport mechanisms in addition to, or instead of, passive diffusion.

Finally, comparing the ligands containing a phenol antioxidant moiety to closely related ligands without this moiety (6d*vs.*6c, 10d*vs.*10a), it is evident that the presence of a phenol moiety does not confer a significantly higher degree of neuroprotection to 6-OHDA neurotoxin insult, apart from the slightly higher levels of neuroprotection seen with 10d relative to 10a at 100 μM and 300 μM doses. Taken together, the results of these *in vitro* neuroprotection measurement studies suggest that all compounds screened provided partial neuroprotection against 6-OHDA-induced neurotoxicity at higher molar concentrations (100 μM and 300 μM), with 6a and 6c offering the most efficient levels of protection in this particular cell model of PD. At a dose level of 100 μM, compounds 6a and 6c offered comparable levels of neuroprotection to those seen with DFP 1 and with the 1-hydroxypyridin-2(1*H*)-ones 2 and 3 studied previously (see Fig. S22† for a comparison).^27^

## Conclusions

In conclusion, we have synthesized and evaluated some novel 1-hydroxypyrazin-2(1*H*)-one iron chelator ligands as potential multifunctional disease modifying therapies for the treatment of PD. Two different synthetic routes to these ligands have been explored, and their physicochemical properties, iron chelation potential and antioxidant capacities have been determined. It has been found that 1-hydroxypyrazin-2(1*H*)-ones are more acidic than the closely related 1-hydroxypyridin-2(1*H*)-ones, although the two families of ligands have comparable distribution coefficients at physiological pH. In addition, 1-hydroxypyrazin-2(1*H*)-ones have weaker iron chelation abilities and form less stable iron complexes than 1-hydroxypyridin-2(1*H*)-ones. Ligand 10a showed the best radical trapping capacities of the studied ligands in two antioxidant assays, and the results were comparable to the clinically used iron chelator DFP 1. Despite their weaker iron chelating abilities and higher acidities, 1-hydroxypyrazin-2(1*H*)-ones showed similar neuroprotective cell rescue effects to the previously studied 1-hydroxypyridin-2(1*H*)-ones and the 3-hydroxypyridin-4(1*H*)-one DFP 1.^27^ Ligands 6a and 6c are able to restore neuronal cell viability to ≥89% at optimal doses, while the remaining compounds provided partial neuroprotection against 6-OHDA neurotoxin insult *in vitro*. Surprisingly, the ligands containing an additional phenol antioxidant moiety (6d and 10d) did not provide significantly higher levels of neuroprotection than those without this moiety. Our results suggest that 1-hydroxypyrazin-2(1*H*)-ones are also worthy of consideration amongst the broader hydroxypyridinone (HOPO) family of iron chelators for further development as disease modifying therapies for the treatment of Parkinson's disease, and that the neuroprotective effects of these compounds are primarily due to their ability to chelate and passivate intracellular labile iron, and prevent the generation of free radicals and ROS which would otherwise lead to oxidative stress and ultimately neuronal cell death. Further studies on other iron chelating heterocycles related to 1-hydroxypyrazin-2(1*H*)-ones are currently underway in our laboratories.

Since multiple, complex pathways are implicated in PD, it will be essential for the neuroprotective ability of the 1-hydroxypyrazin-2(1*H*)-one chelators demonstrated here, to be validated against a variety of PD-relevant toxic mechanisms of action. Moreover, future efficacy studies with the lead compounds identified here, to establish the molecular mechanistic basis for how the compounds interact with both 6-OHDA as well as other neurotoxins’ toxic mode of action, are warranted. For instance, it may be that in addition to the compounds’ iron chelation ability, they can decrease production of 6-OHDA-induced metal-catalysed lipid peroxide, which induce cell damage.^48^

A further consideration for future work concerns the preferential subcellular distribution of our compounds. In particular, recent interest in iron chelators capable of penetrating the subcellular mitochondria has shown therapeutic promise in *in vitro* models of PD.^49^ Since mitochondria are the principal destination for labile iron, this feature makes these organelles particularly susceptible to oxidative damage, which is a biochemical feature of PD. Hence, compounds capable of chelating excess iron within mitochondria could represent a therapeutic step forward for treating neurodegenerative conditions involving both iron accumulation and oxidative damage components. The ability of the series of 1-hydroxypyrazin-2(1*H*)-ones presented here to alter mitochondrial functions should also be assessed, using mitochondria-specific toxins such as rotenone, a potent complex I electron transport chain inhibitor.

## Experimental

### General procedures

All solvents and reagents were purchased from Sigma-Aldrich, Acros Organics or Alfa-Aesar and used without further purification unless otherwise specified. Reactions were monitored by TLC using silica gel with UV_254_ fluorescent indicator. Uncorrected melting points were measured in open capillary tubes using a DigiMelt MPA161 SRS instrument. NMR spectra were recorded on either a JEOL JNM-EX270FT Delta spectrometer (270.17 MHz for ^1^H NMR, 67.93 MHz for ^13^C NMR) or on a JEOL ECS400FT Delta spectrometer (399.78 MHz for ^1^H NMR, 100.53 MHz for ^13^C NMR). Chemical shifts are reported in parts per million (ppm) relative to tetramethylsilane as internal standard. Coupling constants (*J*) are measured in hertz. Multiplets are reported as follows: br = broad, s = singlet, d = doublet, dd = double doublet, t = triplet, q = quartet, qu = quintet, s = sextet, sp = septet, m = multiplet, app d = apparent doublet, app t = apparent triplet. Low resolution mass spectra were obtained in methanol solutions on a Thermo Finnigan LCQ Advantage MS detector using electrospray ionisation (ESI). High resolution mass spectra were obtained on a Finnigan MAT900XLT high-resolution double focussing MS spectrometer using nano-electrospray ionisation (NESI) at the EPSRC National Mass Spectrometry Service (University of Swansea). Column chromatography was conducted using 0.060–0.20 mm silica gel (70–230 mesh), and automated flash column chromatography was performed using a Biotage Isolera One ISO-1SV instrument. The calculated partition coefficients (C log *P*) for compounds 6a–6d, 10a, 11a and DFP 1 were determined using ACD-I/Lab (available at: https://www.psds.ac.uk/). The BBB scores of 6a–6d, 10a, 11a, 2, 3 and DFP 1 were obtained by inputting the structural parameters of the compounds (obtained from SwissADME at: http://www.swissadme.ch/) into the previously published algorithm.^32^ The synthesis of known compounds 8^50^ and 9^51^ is described in the ESI.†

### Glycine hydroxamic acid 5a^33,34*a*,*b*^

**Method A**. To a solution of glycine ethyl ester hydrochloride 4a (6.02 g, 43.129 mmol) in water (4 mL) was added hydroxylamine hydrochloride (2.997 g, 43.129 mmol, 1 eq.). The solution was cooled to 0 °C and aqueous sodium hydroxide (11.8 mL, 12 M, 3.3 eq.) was added dropwise over 10 minutes. The solution was stirred at 0 °C for 30 minutes and was then allowed to warm to room temperature and stirring was continued for 24 hours. Aqueous hydrochloric acid (37%, 4.3 mL, 10 M, 1 eq.) was then added to bring the solution to pH 2 and the solution was cooled to 0 °C. The precipitated solid was filtered and washed with cold water (10 mL) and was allowed to dry in air to afford glycine hydroxamic acid 5a as a white solid (2.49 g, 64%). **Method B**. Glycine ethyl ester hydrochloride 4a (5.72 g, 40.980 mmol) was dissolved in a solution of sodium hydroxide (1.64 g, 40.980 mmol, 1 eq.) in methanol (50 mL) in a beaker. The precipitated sodium chloride was filtered off to afford a solution of glycine ethyl ester in methanol. In a separate beaker, hydroxylamine hydrochloride (2.85 g, 40.980 mmol, 1 eq.) was dissolved in a solution of sodium hydroxide (1.64 g, 40.980 mmol, 1 eq.) in methanol (50 mL). The precipitated sodium chloride was filtered off to afford a solution of hydroxylamine in methanol. This solution was added slowly dropwise to the solution of glycine ethyl ester at 0 °C over 1 hour. The solution was stirred at 0 °C for 1 hour, and was then allowed to warm to room temperature and stirring was continued for a further 24 hours. The precipitated solid was filtered and washed with methanol (20 mL) and was allowed to dry in air to afford glycine hydroxamic acid 5a as a white solid (2.06 g, 56%). *δ*_H_ (399.8 MHz, D_2_O) 3.16 (2H, s, C*H*_2_). *δ*_H_ (399.8 MHz, DMSO-*d*_6_) 2.94 (2H, s, C*H*_2_).

### Alanine hydroxamic acid 5b^34*b*,*c*^

To a solution of alanine ethyl ester hydrochloride 4b (0.50 g, 3.255 mmol) in water (1 mL) was added hydroxylamine hydrochloride (0.23 g, 3.255 mmol, 1 eq.). The solution was cooled to 0 °C and aqueous sodium hydroxide (0.89 mL, 12 M, 3.3 eq.) was added dropwise over 5 minutes. The solution was stirred at 0 °C for 30 minutes and was allowed to warm to room temperature and stirring was continued for a further 24 hours. Aqueous hydrochloric acid (37%, 0.32 mL, 10 M, 1 eq.) was added and the solution was cooled to 0 °C for 15 minutes. The precipitated solid was filtered and washed with cold water (5 mL) and was allowed to dry in air to afford alanine hydroxamic acid 5b as a white solid (0.19 g, 56%). *δ*_H_ (399.8 MHz, D_2_O) 1.12 (3H, d, *J* 7.2, C*H*_3_CH), 3.36 (1H, q, *J* 7.2, CH_3_C*H*).

### Phenylalanine hydroxamic acid 5c^33*b*,37^

Phenylalanine benzyl ester hydrochloride 4c (3.00 g, 10.28 mmol) was dissolved in a solution of sodium hydroxide (0.41 g, 10.28 mmol, 1 eq.) in methanol (30 mL) in a beaker. This afforded a clear yellow solution of phenylalanine benzyl ester in methanol. In a separate beaker, hydroxylamine hydrochloride (2.14 g, 30.85 mmol, 3 eq.) was dissolved in a solution of sodium hydroxide (1.23 g, 30.85 mmol, 3 eq.) in methanol (50 mL). The precipitated sodium chloride was filtered off to afford a solution of hydroxylamine in methanol. This solution was added slowly dropwise to the solution of phenylalanine benzyl ester at −30 °C over 1 hour. The solution was stirred at −30 °C for 1 hour, and was then allowed to warm to room temperature and stirring was continued for a further 24 hours. The precipitated solid was filtered and washed with methanol (20 mL) and was allowed to dry in air to afford phenylalanine hydroxamic acid 5c as a white solid (1.04 g, 56%). *δ*_H_ (399.8 MHz, DMSO-*d*_6_) 2.56 (1H, dd, *J* 13.2 and 6.4, ArC*H*_2_CH), 2.79 (1H, dd, *J* 13.2 and 6.4, ArC*H*_2_CH), 3.19 (1H, t, *J* 6.4, ArCH_2_C*H*), 7.13–7.24 (5H, m, Ar*H*).

### Tyrosine hydroxamic acid 5d^38^

Tyrosine ethyl ester hydrochloride 4d (3.00 g, 1.221 mmol) was dissolved in a solution of sodium hydroxide (0.488 g, 1.221 mmol, 1 eq.) in methanol (50 mL) in a beaker. This afforded a clear brown solution of tyrosine ethyl ester in methanol. In a separate beaker, hydroxylamine hydrochloride (2.54 g, 3.663 mmol, 3 eq.) was dissolved in a solution of sodium hydroxide (1.46 g, 3.663 mmol, 3 eq.) in methanol (100 mL). The precipitated sodium chloride was filtered off to afford a solution of hydroxylamine in methanol. This solution was added slowly dropwise to the solution of tyrosine ethyl ester at 0 °C over 1 hour. The solution was stirred at 0 °C for 1 hour, and was then allowed to warm to room temperature and stirring was continued for a further 24 hours. The clear solution was evaporated to a small volume and the precipitated solid was filtered and washed with methanol (10 mL) and was allowed to dry in air to afford tyrosine hydroxamic acid 5d as a pale pink solid (0.814 g, 34%). *δ*_H_ (399.8 MHz, DMSO-*d*_6_) 2.43 (1H, dd, *J* 13.2 and 6.0, ArC*H*_2_CH), 2.67 (1H, dd, *J* 13.2 and 6.0, ArC*H*_2_CH), 3.09 (1H, m, ArCH_2_C*H*), 6.59 (2H, d, *J* 8.4, 2 × Ar*H*), 6.91 (2H, d, *J* 8.4, 2 × Ar*H*), 8.66 (1H, br s, NHO*H*), 9.12 (1H, br s, ArO*H*).

### 1-Hydroxy-5,6-dimethylpyrazin-2(1*H*)-one 6a^30,31,33*b*,35^

To a solution of glycine hydroxamic acid 5a (0.70 g, 7.777 mmol) in methanol (50 mL) and water (30 mL) at 0 °C was added a solution of 2,3-butanedione (0.78 mL, 1.15 eq.) in methanol (10 mL). Aqueous sodium hydroxide (1.0 mL, 2 M) was added dropwise over 5 minutes and the solution was stirred at 0 °C for 1 hour. The solution was then allowed to warm to room temperature and stirring was continued for 24 hours. Aqueous hydrochloric acid (37%, 10 M) was then added dropwise to bring the solution to pH 3 and the solution was evaporated to a small volume. The solution was diluted with water (20 mL) and extracted with chloroform (5 × 50 mL). The combined organic extracts were dried and evaporated to afford a light brown solid (0.65 g). The solid was triturated with acetone (10 mL) and the insoluble solid was filtered and washed with acetone to afford 1-hydroxy-5,6-dimethylpyrazin-2(1*H*)-one 6a as a light brown solid (0.26 g, 24%). *δ*_H_ (399.8 MHz, CDCl_3_, Me_4_Si) 2.40 (3H, s, 5-C*H*_3_), 2.48 (3H, s, 6-C*H*_3_), 8.10 (1H, s, 3-Ar*H*), 8.33 (1H, br s, 1-O*H*). *δ*_H_ (399.8 MHz, D_2_O) 2.29 (3H, s, 5-C*H*_3_), 2.30 (3H, s, 6-C*H*_3_), 7.50 (1H, s, 3-Ar*H*). *δ*_C_ (100.5 MHz, CDCl_3_, Me_4_Si) 12.9 (5-*C*H_3_), 20.1 (6-*C*H_3_), 133.0 (quat), 133.9 (quat), 137.1 (C-3), 157.3 (C-2).

### 1-Hydroxy-3-benzyl-5,6-dimethylpyrazin-2(1*H*)-one 6c^33*b*^

To a solution of phenylalanine hydroxamic acid 5c (0.93 g, 5.16 mmol) in methanol (56 mL) and water (28 mL) at −30 °C was added a solution of 2,3-butanedione (0.56 mL, 5.67 mmol, 1.1 eq.) in methanol (18 mL). Aqueous sodium hydroxide (2.32 mL, 2 M) was added dropwise over 15 minutes and the solution was stirred at −30 °C for 1 hour. The solution was then allowed to warm to room temperature and stirring was continued for a further 24 hours. Aqueous hydrochloric acid (37%, 10 M) was then added dropwise to bring the solution to pH 3 and the solution was evaporated to a small volume. The solution was extracted with chloroform (5 × 50 mL). The combined organic extracts were dried and evaporated to afford a brown oil. The oil was triturated with ether (15 mL) and the insoluble solid was filtered and washed with ether (5 mL) to afford 1-hydroxy-3-benzyl-5,6-dimethylpyrazin-2(1*H*)-one 6c as a light orange solid (0.28 g, 24%). *δ*_H_ (399.8 MHz, CDCl_3_, Me_4_Si) 2.36 (3H, s, 5-C*H*_3_), 2.40 (3H, s, 6-C*H*_3_), 4.14 (2H, s, 3-C*H*_2_), 7.16–7.39 (5H, m, 5 × Ar*H*). *δ*_C_ (100.5 MHz, CDCl_3_, Me_4_Si) 12.8 (5-*C*H_3_), 20.0 (6-*C*H_3_), 39.6 (3-*C*H_2_), 126.6 (1 × Ar*C*), 128.5 (2 × Ar*C*), 129.2 (2 × Ar*C*), 130.2 (quat), 131.6 (quat), 137.8 (quat), 148.1 (quat), 150.0 (C-2).

### 1-Hydroxy-3-(4-hydroxybenzyl)-5,6-dimethylpyrazin-2(1*H*)-one 6d

To a solution of tyrosine hydroxamic acid 5d (0.40 g, 0.204 mmol) in methanol (30 mL) and water (17 mL) at 0 °C was added a solution of 2,3-butanedione (0.193 g, 0.197 mL, 0.224 mmol, 1.1 eq.) in methanol (8 mL). Aqueous sodium hydroxide (1.0 mL, 2 M) was added dropwise over 10 minutes and the solution was stirred at 0 °C for 1 hour. The solution was then allowed to warm to room temperature and stirring was continued for a further 24 hours. Aqueous hydrochloric acid (37%, 10 M) was then added dropwise to bring the solution to pH 3 and the solution was evaporated to a small volume. The solution was extracted with chloroform (5 × 50 mL). The combined organic extracts were dried and evaporated to afford a light brown solid. The solid was triturated with DCM (10 mL) and the insoluble solid was filtered and washed with DCM (5 mL) to afford 1-hydroxy-3-(4-hydroxybenzyl)-5,6-dimethylpyrazin-2(1*H*)-one 6d as a light brown solid (0.21 g, 42%). Mp 196.5–197.8 °C (from chloroform). *δ*_H_ (399.8 MHz, DMSO-*d*_6_) 2.18 (3H, s, 5-C*H*_3_), 2.21 (3H, s, 6-C*H*_3_), 3.81 (2H, s, 3-C*H*_2_), 6.58 (2H, d, *J* 8.4, 2 × Ar*H*), 7.01 (2H, d, *J* 8.4, 2 × Ar*H*), 9.14 (1H, s, ArO*H*). *δ*_C_ (100.5 MHz, DMSO-*d*_6_) 13.2 (5-*C*H_3_), 19.9 (6-*C*H_3_), 38.6 (3-*C*H_2_), 115.5 (2 × Ar*C*), 128.2 (quat), 129.0 (quat), 130.3 (2 × Ar*C*), 133.2 (quat), 151.3 (quat), 152.0 (quat), 156.2 (C-2). *m*/*z* (ESI) 269.0898 ([M + Na]^+^); C_13_H_14_N_2_O_3_Na requires 269.0902.

### 1-Hydroxy-6-phenylpyrazin-2(1*H*)-one 10a

To a solution of glycine hydroxamic acid 5a (0.35 g, 3.889 mmol) in ethanol (30 mL) and water (30 mL) was added phenylglyoxal (0.54 g, 4.083 mmol, 1.05 eq.). The solution was heated under reflux for 24 hours. The flask was allowed to cool to room temperature and the solvents were evaporated to afford crude 10a as a light brown solid (0.68 g). The crude solid was triturated with methanol (10 mL) and the insoluble solid was filtered and washed with methanol (15 mL) and diethyl ether (20 mL). The solid was allowed to dry in air to afford 1-hydroxy-6-phenylpyrazin-2(1*H*)-one 10a as a light brown solid (0.22 g, 30%). Mp 142.5–144.5 °C (from EtOH/water). *δ*_H_ (399.8 MHz, DMSO-*d*_6_) 7.20 (1H, t, *J* 7.4, Ar*H*), 7.33 (2H, t, *J* 7.4, 2 × Ar*H*), 7.69 (1H, s, 5-Ar*H*), 7.79 (2H, d, *J* 7.4, 2 × Ar*H*), 8.41 (1H, s, 3-Ar*H*). *δ*_H_ (399.8 MHz, D_2_O) 7.19–7.29 (3H, m, 3 × Ar*H*), 7.45–7.47 (2H, m, 2 × Ar*H*), 7.72 (1H, s, 5-Ar*H*), 8.02 (1H, s, 3-Ar*H*). *δ*_C_ (100.5 MHz, DMSO-*d*_6_) 124.9 (2 × Ar*C*), 127.3 (Ar*C*), 128.6 (C-5), 129.1 (2 × Ar*C*), 133.4 (quat), 137.2 (quat), 138.7 (C-3), 156.9 (C-2). *δ*_C_ (100.5 MHz, D_2_O) 125.7 (2 × Ar*C*), 128.4 (Ar*C*), 128.9 (2 × Ar*C*), 129.5 (C-5), 135.3 (quat), 137.6 (quat), 139.8 (C-3), 156.8 (C-2). *m*/*z* (ESI) 189.0659 ([M + H]^+^); C_10_H_9_N_2_O_2_ requires 189.0664.

### 1-Hydroxy-6-(4-methoxyphenyl)-pyrazin-2(1*H*)-one 10b

To a solution of glycine hydroxamic acid 5a (0.296 g, 3.29 mmol) in ethanol (45 mL) and water (45 mL) was added 4-methoxyphenylglyoxal hydrate (0.60 g, 3.29 mmol, 1 eq.). The solution was heated under reflux for 24 hours. The flask was allowed to cool to room temperature and the solvents were evaporated to afford the crude product as a light brown solid. The crude solid was triturated with methanol (10 mL) and the insoluble solid was filtered and washed with methanol (15 mL) and diethyl ether (24 mL). The solid was allowed to dry in air to afford 1-hydroxy-6-(4-methoxyphenyl)-pyrazin-2(1*H*)-one 10b as a light brown solid (0.19 g, 27%). Mp 176.8–179.0 °C (from EtOH/water). *δ*_H_ (399.8 MHz, CDCl_3_, Me_4_Si) 3.85 (3H, s, OC*H*_3_), 6.97 (2H, d, *J* 8.4, 2 × Ar*H*), 7.70 (2H, d, *J* 8.4, 2 × Ar*H*), 8.03 (1H, s, 5-Ar*H*), 8.38 (1H, s, 3-Ar*H*). *δ*_C_ (100.5 MHz, CDCl_3_, Me_4_Si) 55.5 (O*C*H_3_), 114.6 (2 × Ar*C*), 120.3 (C-5), 126.8 (2 × Ar*C*), 127.2 (quat), 127.5 (quat), 130.1 (quat), 144.3 (C-3), 160.3 (C-2). *m*/*z* (ESI) 219.0762 ([M + H]^+^); C_11_H_11_N_2_O_3_ requires 219.0769.

### 1-Hydroxy-6-(4-fluorophenyl)-pyrazin-2(1*H*)-one 10c

To a solution of glycine hydroxamic acid 5a (0.32 g, 3.50 mmol) in ethanol (45 mL) and water (45 mL) was added 4-fluorophenylglyoxal hydrate (0.60 g, 3.50 mmol, 1 eq.). The solution was heated under reflux for 24 hours. The flask was allowed to cool to room temperature and the solvents were evaporated to afford the crude product as a light brown solid. The crude solid was triturated with methanol (10 mL) and the insoluble solid was filtered and washed with methanol (15 mL) and diethyl ether (24 mL). The solid was allowed to dry in air to afford 1-hydroxy-6-(4-fluorophenyl)-pyrazin-2(1*H*)-one 10c as a light brown solid (0.16 g, 24%). Mp 210.6–212.5 °C (from EtOH/water). *δ*_H_ (399.8 MHz, DMSO-*d*_6_) 7.22 (2H, t, *J* 8.8, 2 × Ar*H*), 7.89 (2H, dd, *J* 8.8 and 5.6, 2 × Ar*H*), 8.18 (1H, s, 5-Ar*H*), 8.61 (1H, s, 3-Ar*H*). *δ*_C_ (100.5 MHz, DMSO-*d*_6_) 116.1 (d, *J* 83.6, 2 × Ar*C*), 116.8 (quat), 120.5 (quat), 125.5 (C-5), 127.3 (d, *J* 34, 2 × Ar*C*), 132.0 (d, *J* 167.7, quat), 146.8 (C-3), 151.8 (C-2). *m*/*z* (ESI) 207.0563 ([M + H]^+^); C_10_H_8_N_2_O_2_F requires 207.0569.

### 1-Hydroxy-6-(4-hydroxyphenyl)-pyrazin-2(1*H*)-one 10d

To a solution of 1-hydroxy-6-(4-methoxyphenyl)-pyrazin-2(1*H*)-one 10b (0.050 g, 0.23 mmol) in dry DCM (1.5 mL) was added a solution of boron tribromide (1 M in DCM, 0.69 mL, 0.69 mmol, 3 eq.) *via* syringe. The solution was stirred at room temperature overnight and then water (2 mL) was added. The insoluble solid was filtered and allowed to dry in air to afford 1-hydroxy-6-(4-hydroxyphenyl)-pyrazin-2(1*H*)-one 10d as a dark brown solid (0.010 g, 21%). Mp >260 °C (from DCM). *δ*_H_ (399.8 MHz, DMSO-*d*_6_) 6.76 (2H, d, *J* 8.8, 2 × Ar*H*), 7.59 (2H, d, *J* 8.8, 2 × Ar*H*), 8.11 (1H, s, 5-Ar*H*), 8.28 (1H, s, 3-Ar*H*). *δ*_C_ (100.5 MHz, DMSO-*d*_6_) 116.2 (2 × Ar*C*), 124.1 (C-5), 126.4 (quat), 126.9 (2 × Ar*C*), 133.9 (quat), 146.5 (C-3), 151.9 (quat), 157.7 (C-2). *m*/*z* (ESI) 205.0607 ([M + H]^+^); C_10_H_9_N_2_O_3_ requires 205.0613.

### 1-Hydroxy-6-methylpyrazin-2(1*H*)-one 11a and 1-hydroxy-5-methylpyrazin-2(1*H*)-one 12a

To a solution of glycine hydroxamic acid 4a (0.50 g, 5.555 mmol) in methanol (40 mL) and water (20 mL) at 0 °C was added a solution of pyruvaldehyde (1.15 g, 40 wt% in water, 1.15 eq.) in methanol (15 mL). Aqueous sodium hydroxide (2.0 mL, 2 M) was added dropwise over 15 minutes and the solution was stirred at 0 °C for 1 hour. The solution was then allowed to warm to room temperature and stirring was continued for 24 hours. Aqueous hydrochloric acid (37%, 10 M) was then added dropwise to bring the solution to pH 3 and the solution was evaporated to a small volume. The solution was diluted with water (40 mL) and extracted with chloroform (3 × 100 mL). The combined organic extracts were dried and evaporated to afford a light brown solid. The solid was triturated with diethyl ether (50 mL) and filtered and washed with diethyl ether (50 mL) to afford a 12 : 1 mixture of 1-hydroxy-6-methylpyrazin-2(1*H*)-one 11a together with its regioisomer 1-hydroxy-5-methylpyrazin-2(1*H*)-one 12a as a light brown solid (0.075 g, 11%). Mp 212.5–214.5 °C (from chloroform). The major regioisomer 11a had *δ*_H_ (399.8 MHz, CDCl_3_, Me_4_Si) 2.38 (3H, s, 6-C*H*_3_), 7.55 (1H, s, 5-Ar*H*), 8.25 (1H, s, 3-Ar*H*). *δ*_C_ (100.5 MHz, CDCl_3_, Me_4_Si) 19.4 (6-*C*H_3_), 126.1 (C-5), 131.9 (quat), 146.7 (C-3), 151.8 (C-2). The minor regioisomer 12a had *δ*_H_ (399.8 MHz, CDCl_3_, Me_4_Si) 2.47 (3H, s, 5-C*H*_3_), 7.43 (1H, s, 6-Ar*H*), 8.15 (1H, s, 3-Ar*H*). *m*/*z* (ESI) 127.0504 ([M + H]^+^); C_5_H_7_N_2_O_2_ requires 127.0507.

### Determination of p*K*_a_ values, stability constants and pL_0.5_ values

Distilled water was purified by passing through a mixed bed of ion exchanger (Bioblock Scientific R3-83002, M3-83006) and activated carbon (Bioblock Scientific ORC-83005). All of the stock solutions were prepared by weighing solid products using an AG 245 Mettler Toledo analytical balance (precision 0.01 mg). Fe^3+^ cation stock solution was prepared from its perchlorate salt and its concentration was determined spectrophotometrically.^52^ All experiments were done in duplicate, at least.

The acid–base properties (log *K*) of ligands 6a, 6c, 6d, 10a and 11a and their affinity for Fe^3+^ were determined *via* spectrophotometric titrations *versus* pH between pH 2 and 12. The titrations were carried out in water (*I* = 0.1 M NaClO_4_) for ligands 6a, 10a and 11a and in a mixed MeOH/H_2_O 80/20 w/w (*I* = 0.1 M NaClO_4_) solvent for 6c, 6d and 10a due to their very low solubility in water. Sodium hydroxide (NaOH) and perchloric acid (HClO_4_) were used to adjust pH during titrations. The ionic strength of all the solutions was fixed to 0.1 M with sodium perchlorate (NaClO_4_). The measurement of pH was achieved by the use of combined glass electrodes (Metrohm, 6.0234.100, Long Life) filled with 0.1 M NaCl for studies in water or NaCl in MeOH/H_2_O (80/20 w/w) for studies in MeOH/H_2_O (80/20 w/w). The electrodes were calibrated daily as hydrogen concentration probes by titrating known amounts of hydrochloric acid with CO_3_^2−^-free sodium hydroxide solutions. The GLEE program^53^ was used for the glass electrode calibration with a p*K*_w_ of 13.77 for studies in water and 14.42 for studies in MeOH/H_2_O (80/20 w/w).

Between pH 2 and 12.5, direct titrations were carried out. Typically, an aliquot of 40 mL of ligand solution was introduced into a thermostated jacketed titration vessel (25.0(2) °C), with an additional 0.3 equiv. of Fe^3+^ in the case of complexation titrations. A known volume of perchloric acid solution was added to adjust the pH to around 2, and the titrations were carried out between pH 2 and 12 by addition of known volumes of sodium hydroxide solution with a Metrohm 904 DMS Titrino automatic titrator (Methrom AG; Herisau, Switzerland) equipped with a 2 mL Dosino 800 burette. After each addition, the pH was allowed to equilibrate and a UV-visible spectrum was recorded automatically with an Agilent Cary 60 UV-visible spectrophotometer.

As the Fe^3+^ complexes were already fully formed at pH 2, protonation and complexation studies had to be carried out in very acidic medium. For this purpose, the batch technique was used and a series of samples were prepared between pH ∼−0.6 and pH 2. Each sample was prepared separately by mixing a known amount of ligand, or ligand and Fe^3+^ in case of complexation studies ([Fe^3+^]/[L] = 3) either in water or in a MeOH/H_2_O (80/20 w/w) mixture. The pH of each sample was adjusted by addition of a known calculated amount of HClO_4_ (pH = −log [H^+^]). The ionic strength was not fixed at pH < 1 in the batch titrations and no decomposition of the ligands were observed, even in strongly acidic conditions. An absorption spectrum of each sample was recorded in a 1cm quartz Supra-sil spectrophotometric cell using a Shimadzu UV-2401PC UV-visible spectrophotometer.

The spectrophotometric data were fitted with the HypSpec software7 (http://www.hyperquad.co.uk),^41*a*^ to calculate the protonation constants of the ligands (log *K*), the stability constants (log *β*) of the formed complex species and the coordination model of the studied systems. The data for Fe^3+^ hydrated species and their solubility products were taken into account in the equilibrium model, according to previously published guidelines.^54^

### Determination of distribution coefficients

The lipophilicity of ligands 6a, 6c, 6d, 10a and 11a, along with their corresponding Fe^3+^ complexes, were determined by calculating their respective distribution coefficient (log *D*_7.4_) values using the Shake Flask method, by referring to the published method of Ma *et al.*^55^ The method is based on the principle that the solute's distribution is determined as a ratio of concentrations of the test compound in a solution mixture, consisting of two immiscible phases, namely n-octanol and an aqueous buffer (HEPES buffer, pH 7.4).

### Trolox equivalent antioxidant capacity (TEAC) assay

The antioxidant capacity of ligands 6a, 6c, 6d, 10a and 11a was determined by a TEAC assay using Trolox as standard. TEAC values were calculated according to an ABTS radical cation decolorization assay. 2,2′-Azinobis-(3-ethylbenzothiazoline-6-sulphonic acid diammonium salt (ABTS, 7 mM) was dissolved in water (10 mL) and exposed to potassium persulphate (2.45 mM). After a 16-hour incubation period (dark, room temperature), the resulting solution was diluted with HPLC-grade methanol such that the absorbance of the solution at 745 nm was around 0.7. ABTS˙^+^ solution (2 mL) was loaded into a cuvette and the absorbance was recorded at 745 nm. 50 μL of various concentrations of the given ligand solution were then added, the cuvette was vigorously shaken and the absorbance was measured after 1, 3 and 6 minutes. Data were plotted such that for each time point a linear system of percentage of inhibition of ABTS˙^+^*versus* concentration of ligand was obtained. The slope of each plot was normalised with respect to that of Trolox to give the Trolox equivalence value for each time point. Compounds and standards were checked to ensure that they did not absorb at 745 nm.

### DPPH antioxidant assay

The 2,2-diphenyl-1-picrylhydrazyl (DPPH) assay was performed according to previously published methods.^56^ For this, 50 μL of different concentrations of ligands 6a, 6c, 6d, 10a and 11a and DFP 1 (0.01 to 0.1 mg mL^−1^ in methanol) was dissolved in 2 mL of DPPH methanolic solution (the concentration was adapted in order to achieve an absorbance of ∼1). The samples were shaken vigorously and allowed to stand until the reaction reached steady-state. The absorbance of the samples was then measured at 515 nm using a UV-visible absorption spectrophotometer (UV-2401 PC; Shimadzu Corporation, Kyoto, Japan). Scavenging of DPPH free radicals was calculated as: DPPH scavenging activity (%) = [(*A*_c_ − *A*_t_)/*A*_c_] × 100 where, *A*_c_ is the absorbance of the control tube (containing all reagents except the test compound) and *A*_t_ is the absorbance of the test tube.

### Determining potential compound toxicity and neuroprotection against 6-OHDA insult in SH-SY5Y neuroblastoma cells

Human SH-SY5Y neuroblastoma cells (ATCC® CRL-2266™) were plated at 20 000 cells per well in 96-well microplates, and left to adhere overnight to the well surface, in cell culture media composed of 50% advanced minimum essential medium (MEM), 50% Ham's F12 medium, 1% l-glutamine, that was supplemented with 2% fetal bovine serum (FBS). All media components were obtained from Thermo Fisher Scientific (Gibco™).

Each well's media was then replaced with 100 μL per well of serum free media, containing ascending concentrations of the novel compounds that ranged from 0–300 μM. Cells were pre-incubated with the compounds for 1 hour at 37 °C, 5% CO_2_, before exposure to 50 μM 6-OHDA (apart from the wells containing non-toxin treated control cells) for a further 24 hours. Just before application to cells, a 10 mM 6-OHDA hydrochloride (Sigma-Aldrich) stock concentration was prepared, by dissolving the powder in 1 mL distilled water containing 0.1% ascorbic acid (Sigma-Aldrich); the solution was protected against light and kept on ice until use.

After a further 24 hours incubation period at 37 °C in a 5% CO_2_ environment, cell viability was measured using the tetrazolium dye, 3-(4,5-dimethylthiazol-2-yl)-2,5-diphenyltetrazolium bromide (MTT). For this colorimetric assay, 11 μL of MTT (5 mg mL^−1^) was added to the 100 μL media per well, and then incubated for 3 hours at 37 °C, 5%, CO_2_. For lysing the cells, an equal volume of solubilizing solution (dimethyl sulfoxide (DMSO)) was added to each well and mixed thoroughly. Absorption was measured at a wavelength of 570 nm by using a scanning multiwell spectrophotometer (Sunrise Tecan, Durham, NC, USA). Cell viability was calculated as a percentage compared to the untreated (not receiving toxin or drug compound) control wells. Data is expressed as the means ± the SEM of three independent experiments. Statistical analyses of the MTT-derived cell viability data were performed using Prism software (v3, GraphPad, San Diego, CA, USA) and using a one-way ANOVA, followed by a Dunnett's *post-hoc* test for multiple comparisons. Results were considered significant at *P* < 0.05.

## Author contributions

FWL and DT conceived the design of the HOPY compounds, JB and VHB conceived their physicochemical evaluation, and JAD and ISP conceived the biological screening assays. FWL, KB, JPN, REF, JB, AT, SB and MM performed all experiments, and FWL, JAD and ISP obtained funding for the work and analysed the data. All authors wrote and reviewed the final version of the manuscript.

## Conflicts of interest

There are no conflicts of interests to declare.

## Supplementary Material

DT-051-D1DT02604F-s001
