# Supplementary material for: Synthesis, physicochemical characterization and neuroprotective evaluation of novel 1-hydroxypyrazin-2(1H)-one iron chelators in an in vitro cell model of Parkinson's disease
Source: Dalton Trans. 2022 Feb 11;51(9):3590–603. doi: 10.1039/d1dt02604f (PMC8886574; doi:10.1039/d1dt02604f)
Supplement: DT-051-D1DT02604F-s001 [file DT-051-D1DT02604F-s001.pdf]

Electronic Supplementary Material (ESI) for Dalton Transactions.
This journal is © The Royal Society of Chemistry 2022

ELECTRONIC SUPPLEMENTARY INFORMATION

for the paper entitled

Synthesis, Physicochemical Characterization and Neuroprotective Evaluation of Novel 1-hydroxypyrazin-2(1*H*)-one Iron Chelators in an *In Vitro* Cell Model of Parkinson's Disease

Frank W. Lewis,*[a] Kathleen Bird,[a] Jean-Philippe Navarro,[a] Rawa El Fallah,[b] Jeremy Brandel,[b] Véronique Hubscher-Bruder,[b] Andrew Tsatsanis,[c,d] James A. Duce,[c,d] David Tétard,[a] Samuel Bourne,[e] Mahmoud Maina[e] and Ilse S. Pienaar[e,f]

[a] *Department of Applied Sciences, Faculty of Health and Life Sciences, Northumbria University, Newcastle upon Tyne, Tyne and Wear NE1 8ST, UK. E-mail: frank.lewis@northumbria.ac.uk*

[b] *Université de Strasbourg, CNRS, IPHC UMR 7178, F-67000 Strasbourg, France. E-mail: j.brandel@unistra.fr*

[c] *School of Biomedical Sciences, The Faculty of Biological Sciences, University of Leeds, Leeds, West Yorkshire LS2 9JT, UK.*

[d] *Alzheimer's Research UK Cambridge Drug Discovery Institute, Cambridge Bio-medical Campus, University of Cambridge, Cambridge, UK. E-mail: jad205@cam.ac.uk*

[e] *School of Life Sciences, University of Sussex, Falmer, Sussex BN1 9PH, UK. E-mail: I.S.Pienaar@sussex.ac.uk*

[f] *Institute of Clinical Sciences, University of Birmingham, Edgbaston, Birmingham B15 2TT, UK.*

CONTENTS		PAGE

1.	**Organic Synthesis**	S3
2.	**Experimental Procedures**	S7
3.	**NMR Spectra**	S9
4.	**Mass Spectra**	S46
5.	**Determination of pKa Values of the Ligands and Stability Constants of the Complexes**	S55
6.	**BBB Penetration Scores**	S64
7.	**DPPH Antioxidant Assay**	S75
8.	**Trolox Equivalent Antioxidant Capacity (TEAC) Assay**	S78
9.	**Neuroprotection against 6-OHDA Neurotoxicity**	S81
10.	**References**	S82

1. Organic Synthesis

Discussion

The target 1-hydroxypyrazin-2(1*H*)-ones **6** were synthesized in two steps from amino acid ethyl esters following the literature procedures as shown below in Scheme S1.[1] Initially, reaction of glycine ethyl ester hydrochloride **4a** with hydroxylamine hydrochloride in alkaline water afforded the known glycine hydroxamic acid **5a** in 64 % yield.[1a,2] Condensation reaction of **5a** with 2,3-butanedione afforded the known 1-hydroxypyrazin-2(1*H*)-one **6a**[1b,3–5] in 24 % yield (Scheme S1). Unfortunately, application of this two-step procedure to the synthesis of **6b** from alanine ethyl ester **4b** failed to give the desired product, due to the high solubility of the hydroxamic acid **5b** in water. We subsequently modified this procedure by using methanol as the solvent and we were able to obtain **5a** from **4a** in 56 % yield (Scheme S1). However, application of this modified procedure to the synthesis of alanine hydroxamic acid **5b**[2b,2c,6] from alanine ethyl ester **4b** gave a mixture of **5b** and another compound (presumed to be the corresponding diketopiperazine) in low yield as judged by ^{1}H NMR spectroscopy. Reaction of this mixture with 2,3-butanedione gave an intractable mixture of products from which the novel 1-hydroxypyrazin-2(1*H*)-one **6b** could not be isolated by chromatography. However, 1-hydroxypyrazin-2(1*H*)-ones **6c** and **6d** were successfully obtained by this modified procedure from the known hydroxamic acids **5c**[1b,2b,7] and **5d**,[8] albeit in only 13 % and 14 % overall yields from **4c** and **4d**, respectively (Scheme S1). There are some reports of multifunctional hydroxypyridinone metal chelators containing phenolic antioxidant moieties that show promising efficacy against neurodegenerative diseases by acting as radical traps as well as metal chelators.[9] Accordingly, we synthesized 1-hydroxypyrazin-2(1*H*)-one **6d** that contains a phenol moiety which could provide a beneficial antioxidant mode of action in addition to iron chelation. Unfortunately, all our attempts to isolate hydroxamic acids **5e**–**5g** from amino esters **4e**–**4g** met with no success.

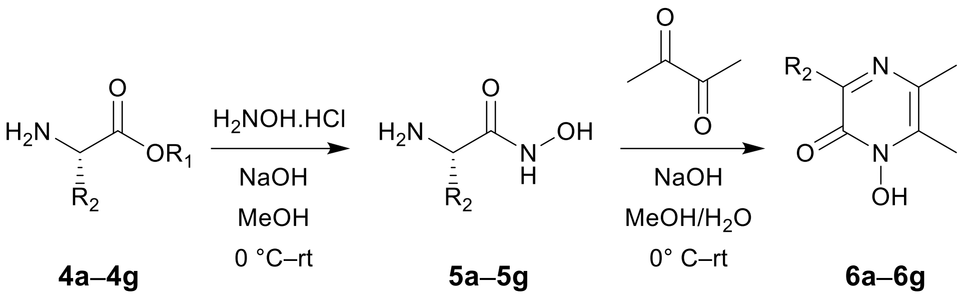

4a–4g **5a–5g** **6a–6g**

H-Gly-OEt **a** R_1 = Et, R_2 = H
H-Ala-OEt **b** R_1 = Et, R_2 = Me
H-Phe-OBn **c** R_1 = Bn, R_2 = $PhCH_2$
H-Tyr-OEt **d** R_1 = Et, R_2 = 4-$HOC_6H_4CH_2$
H-Val-OEt **e** R_1 = Et, R_2 = *i*-Pr
H-Leu-OEt **f** R_1 = Et, R_2 = *i*-$PrCH_2$
H-Ile-OEt **g** R_1 = Et, R_2 = $CH_3CH_2CH(Me)$

Scheme S1. Synthesis of 1-hydroxypyrazin-2(1*H*)-ones **6a**–**6g**.

Due to the low yields obtained above and the failure to synthesize certain 1-hydroxypyrazin-2(1*H*)-ones **6** by the procedure shown in Scheme S1, we sought a more general synthetic method which could be applied to the synthesis of a broader range of these compounds. The synthesis of 1-hydroxypyrazin-2(1*H*)-ones **6** in 4 steps from *N*-Boc amino acids *via* their protected hydroxamic acid benzyl esters was previously reported.[3–5,10] Inspired by this approach, we explored a new synthesis of 1-hydroxypyrazin-2(1*H*)-ones **6** from activated *N*-Boc amino acid *N*-hydroxysuccinimide esters **7** as shown below in Scheme S2.

Reaction of *N*-Boc-protected *N*-hydroxysuccinimide esters **7a**, **7b**, **7c**, **7e** and **7f** with *O*-benzylhydroxylamine generated the Boc-protected aminohydroxamic acid benzyl esters **8a**, **8b**, **8c**, **8e** and **8f** in high yields. Subsequent *N*-Boc deprotection (TFA in DCM) gave the free aminohydroxamic acid benzyl esters **9b**, **9c**, **9e** and **9f** in excellent yields. However, despite the known formation of **6'a** from **9a** (as HCl salt) and 2,3-butanedione reported in the literature,[5,11] attempted condensation reactions of compounds **9b**, **9c**, **9e** and **9f** with 2,3-butanedione in our hands failed to generate the desired 1-benzyloxypyrazin-2(1*H*)-ones **6'b**, **6'c**, **6'e** and **6'f**. This synthetic approach was subsequently abandoned in favour of the approach outlined above in Scheme S1.

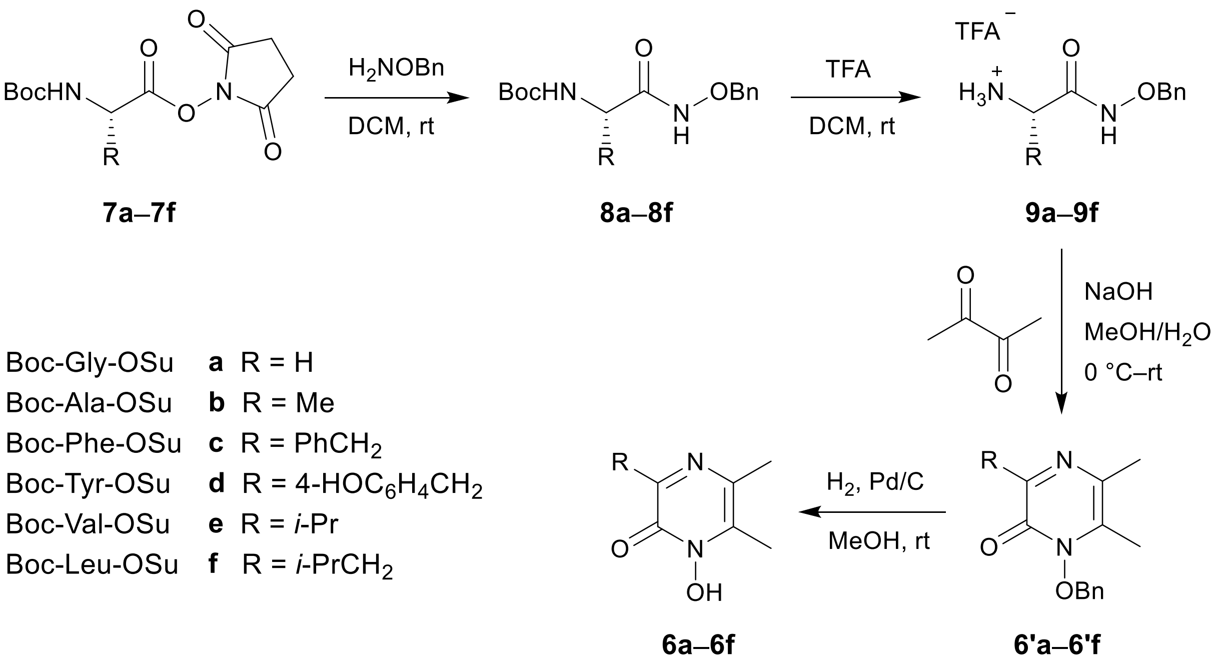

Scheme S2. Attempted synthesis of 1-hydroxypyrazin-2(1*H*)-ones **6a**–**6f**.

We also explored the reactions of glycine hydroxamic acid **5a** with both aromatic and aliphatic α-ketoaldehydes (glyoxals) as shown below in Scheme S3. Reaction of **5a** with phenylglyoxal in ethanol/water at reflux afforded the novel 1-hydroxypyrazin-2(1*H*)-one **10a** in 30 % yield as a single regioisomer. Similarly, reaction of **5a** with 4-methoxyphenylglyoxal and 4-fluorophenylglyoxal gave **10b** and **10c** as single regioisomers in 27 % and 24 % yields, respectively. As with 1-hydroxypyrazin-2(1*H*)-one **6d**, we sought to convert **10b** into a 1-hydroxypyrazin-2(1*H*)-one bearing a phenol moiety with potential antioxidant activity. Accordingly, deprotection of the methoxy group of **10b** with boron tribromide in DCM afforded the novel 1-hydroxypyrazin-2(1*H*)-one **10d** in 21 % yield. Reaction of **5a** with pyruvaldehyde gave the novel 1-hydroxypyrazin-2(1*H*)-ones **11a** and **12a** as a 12:1 mixture of regioisomers, as judged by ^{1}H NMR spectroscopy (Scheme S3). The major regioisomer was tentatively assigned as **11a** on the basis that the free primary amino group of **5a** would preferentially react with the aldehyde carbonyl group of the glyoxal, rather than the less electrophilic ketone carbonyl group. These regioisomers proved inseparable by recrystallisation or chromatography, and were studied without further purification.

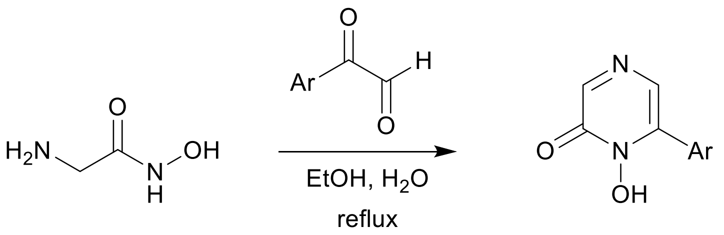

5a

10a Ar = Ph
10b Ar = 4-$MeOC_6H_4$
10c Ar = 4-FC_6H_4
10d Ar = 4-HOC_6H_4

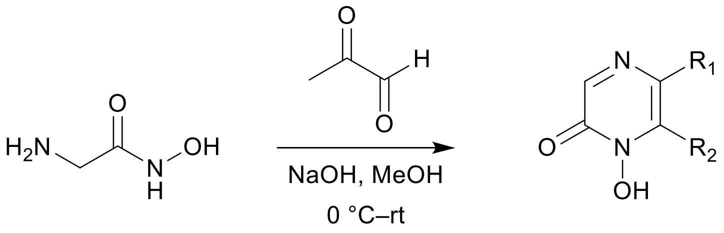

5a

11a R_1 = H, R_2 = Me
12a R_1 = Me, R_2 = H

Scheme S3. Synthesis of 1-hydroxypyrazin-2(1*H*)-ones **10a**–**10d**, **11a** and **12a**.

2. Experimental Procedures

Synthesis of *N*-Boc hydroxamic acid benzyl esters 8a–8f: General procedure

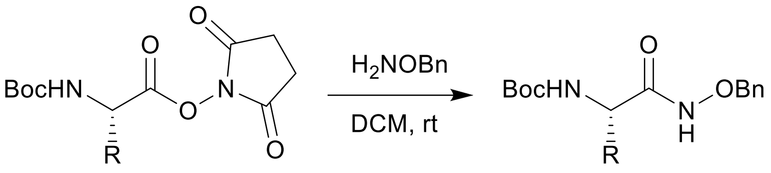

7a–7f **8a–8f**

To a solution of the appropriate *N*-Boc amino acid *N*-hydroxysuccinimide (OSu) ester **7** (1.47 mmol) in DCM (20 mL) at room temperature was added *O*-benzylhydroxylamine (1.47 mmol, 1 eq). The solution was allowed to stir at room temperature for 24 hours. The solvent was evaporated to afford the crude *N*-Boc hydroxamic acid benzyl ester **8** as an oil that crystallised contaminated with *N*-hydroxysuccinimide. This mixture was used in the next step without further purification.

***N*-Boc glycine hydroxamic acid benzyl ester 8a**[3–5,11] δ_H(399.8 MHz, $CDCl_3$, Me_4Si) 1.40 (9H, s, $(CH_3)_3$), 3.67 (2H, s, CH_2NHBoc), 4.82 (1H, br s, N*H*), 4.88 (2H, s, OCH_2Ph), 5.25 (1H, br s, N*H*Boc), 7.36 (5H, s, Ar*H*).

***N*-Boc alanine hydroxamic acid benzyl ester 8b**[11] δ_H(399.8 MHz, $CDCl_3$, Me_4Si) 1.28 (3H, d, *J* 6.8, CH_3CH), 1.38 (9H, s, $(CH_3)_3$), 4.05 (1H, app t, *J* 6.8, CH_3C*H*), 4.86 (2H, s, OCH_2Ph), 5.29 (1H, d, *J* 6.4, N*H*Boc), 7.27–7.36 (5H, m, Ar*H*).

***N*-Boc phenylalanine hydroxamic acid benzyl ester 8c**[12,13] δ_H(399.8 MHz, $CDCl_3$, Me_4Si) 1.36 (9H, s, $(CH_3)_3$), 2.96–3.06 (2H, m, CHCH_2Ph), 4.20 (1H, q, *J* 7.6, C*H*NHBoc), 4.62–4.84 (2H, m, OCH_2Ph), 5.26 (1H, d, *J* 7.6, CHN*H*Boc), 7.17–7.36 (10H, m, Ar*H*).

***N*-Boc valine hydroxamic acid benzyl ester 8e**[12,13] δ_H(399.8 MHz, $CDCl_3$, Me_4Si) 0.88 (3H, d, *J* 6.4, $(CH_3)_2$CH), 0.90 (3H, d, *J* 6.4, $(CH_3)_2$CH), 1.39 (9H, s, $(CH_3)_3$), 1.96–2.02 (1H, m, $(CH_3)_2$C*H*), 3.70 (1H, t, *J* 8.4, C*H*NHBoc), 4.88 (2H, s, OCH_2Ph), 5.28 (1H, d, *J* 8.4, CHN*H*Boc), 7.28–7.37 (5H, m, Ar*H*), 9.45 (1H, br s, N*H*OCH_2Ph).

***N*-Boc leucine hydroxamic acid benzyl ester 8f**[11] δ_H(399.8 MHz, $CDCl_3$, Me_4Si) 0.85–0.87 (6H, m, $(CH_3)_2$CH), 1.39 (9H, s, $(CH_3)_3$), 1.41–1.47 (1H, m, $(CH_3)_2$C*H*), 1.54–1.60 (2H, m, (CH_2CH$(CH_3)_2$),

3.97 (1H, q, *J* 8.0, C*H*NHBoc), 4.88 (2H, s, OC*H*$_2$Ph), 5.17 (1H, d, *J* 8.0, CHN*H*Boc), 7.24–7.37 (5H, m, Ar*H*), 9.45 (1H, br s, N*H*OCH$_2$Ph).

Synthesis of hydroxamic acid benzyl ester TFA salts 9b–9f: General procedure

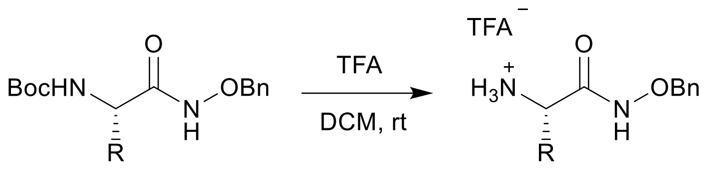

8b–8f **9b–9f**

The appropriate crude *N*-Boc hydroxamic acid benzyl ester **8** (1.47 mmol) was dissolved in DCM (10 mL) and trifluoroacetic acid (10 mL) was added. The solution was allowed to stir at room temperature for 24 hours. The solvents were evaporated to afford the crude TFA salt **9** as a clear oil. The oil was triturated with diethyl ether (10 mL) and the resulting white solid was filtered and washed with diethyl ether (10 mL) and allowed to dry in air to afford the pure TFA salt **9** as a white solid.

Alanine hydroxamic acid benzyl ester TFA salt 9b[4,11] Obtained from **7b** in 87% overall yield. δ_H(399.8 MHz, DMSO-*d*$_6$) 1.24 (3H, d, *J* 7.2, C*H*$_3$CH), 3.64 (1H, br s, CH$_3$C*H*), 4.77 (1H, d, *J* 11.2, OC*H*$_2$Ph), 4.81 (1H, d, *J* 11.2, OC*H*$_2$Ph), 7.34–7.38 (5H, m, Ar*H*).

Phenylalanine hydroxamic acid benzyl ester TFA salt 9c[12,13] Obtained from **7c** in 78% overall yield. δ_H(399.8 MHz, D$_2$O) 2.91 (1H, dd, *J* 14.0 and 8.4, CHC*H*$_2$Ph), 2.99 (1H, dd, *J* 14.0 and 6.8, CHC*H*$_2$Ph), 3.82 (1H, dd, *J* 8.4 and 6.8, C*H*CH$_2$Ph), 4.41 (1H, d, *J* 11.0, OC*H*$_2$Ph), 4.59 (1H, d, *J* 11.0, OC*H*$_2$Ph), 7.06–7.13 (4H, m, Ar*H*), 7.20–7.26 (6H, m, Ar*H*).

Valine hydroxamic acid benzyl ester TFA salt 9e[12,13] Obtained from **7e** in 91% overall yield. δ_H(399.8 MHz, D$_2$O) 0.69 (3H, d, *J* 6.4, (C*H*$_3$)$_2$)CH), 0.73 (3H, d, *J* 6.4, (C*H*$_3$)$_2$)CH), 1.88 (1H, sp, *J* 6.4, (CH$_3$)$_2$C*H*), 3.38 (1H, d, *J* 6.4, C*H*NH$_3^+$), 4.75 (1H, d, *J* 11.2, OC*H*$_2$Ph), 4.80 (1H, d, *J* 11.2, OC*H*$_2$Ph), 7.27–7.32 (5H, m, Ar*H*).

Leucine hydroxamic acid benzyl ester TFA salt 9f[11] Obtained from **7f** in 84% overall yield. δ_H(399.8 MHz, D$_2$O) 0.65 (3H, d, *J* 6.0, (C*H*$_3$)$_2$CH), 0.67 (3H, d, *J* 6.0, (C*H*$_3$)$_2$CH), 1.01–1.10 (1H, m, (CH$_3$)$_2$C*H*), 1.37 (2H, t, *J* 7.2, C*H*$_2$CH(CH$_3$)$_2$), 3.56 (1H, t, *J* 7.2, C*H*NH$_3^+$), 4.74 (1H, d, *J* 11.2, OC*H*$_2$Ph), 4.82 (1H, d, *J* 11.2, OC*H*$_2$Ph), 7.25–7.34 (5H, m, Ar*H*).

3. NMR Spectra

Glycine hydroxamic acid **5a**

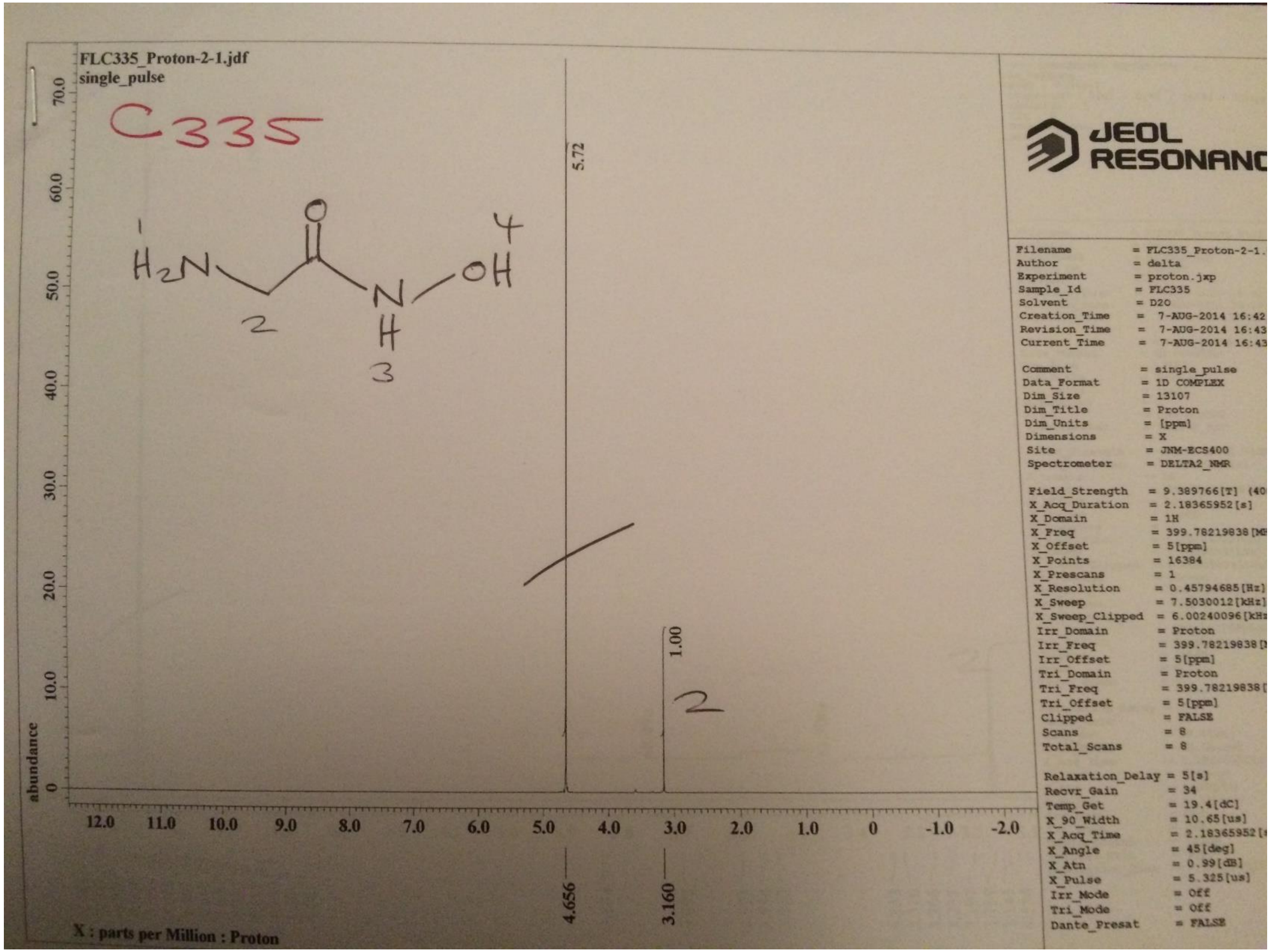

Alanine hydroxamic acid **5b**

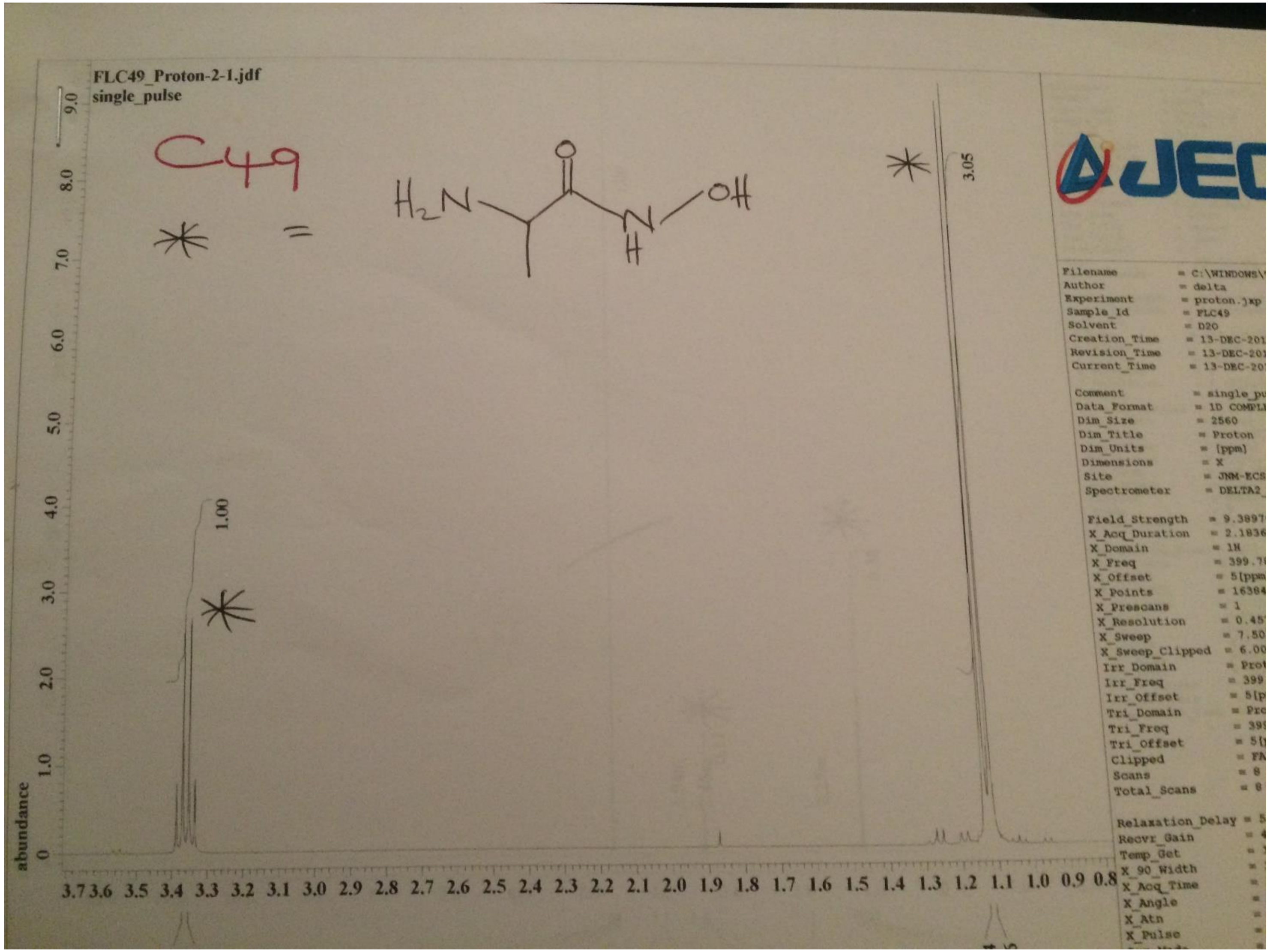

Phenylalanine hydroxamic acid **5c**

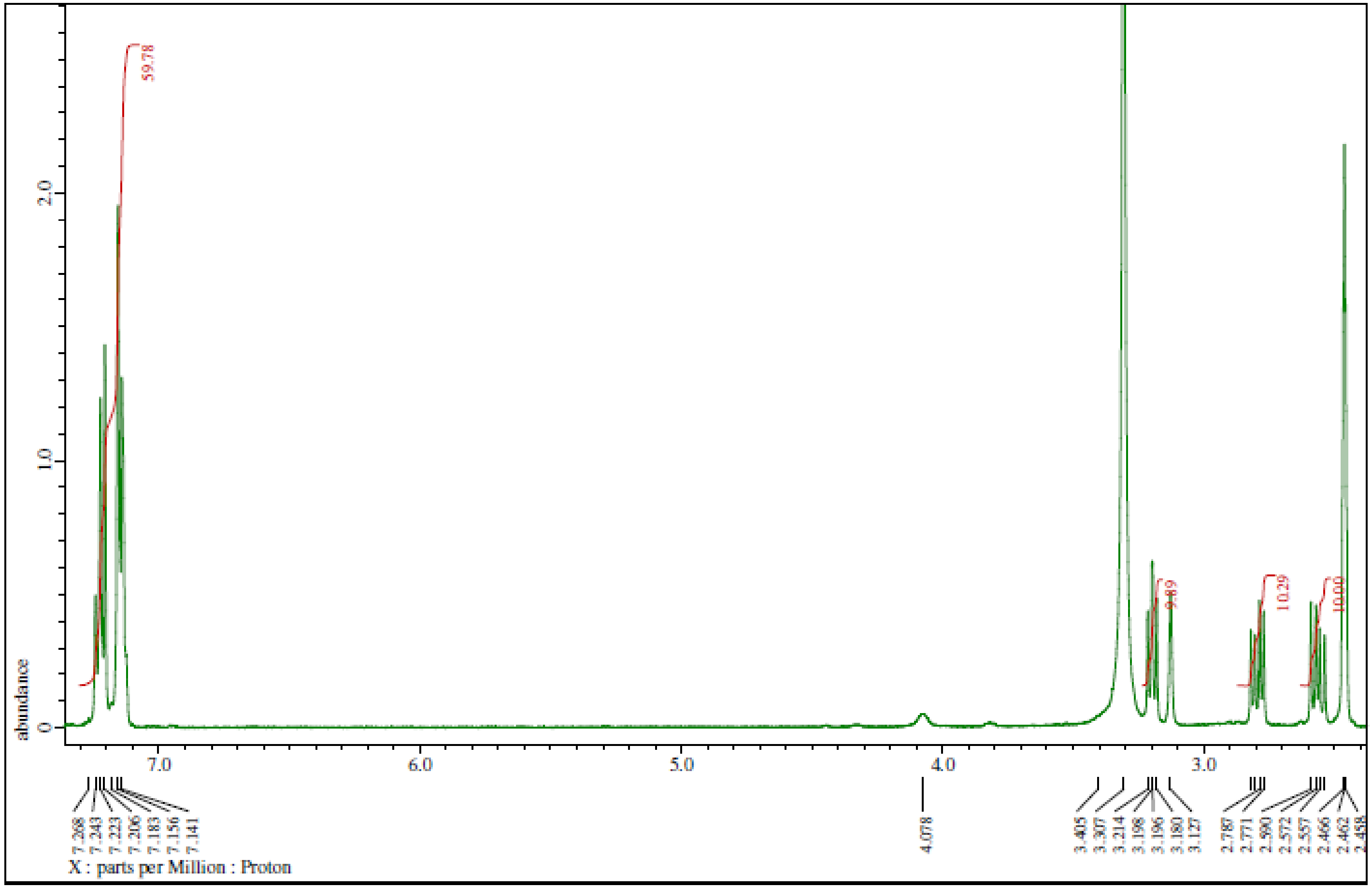

Tyrosine hydroxamic acid **5d**

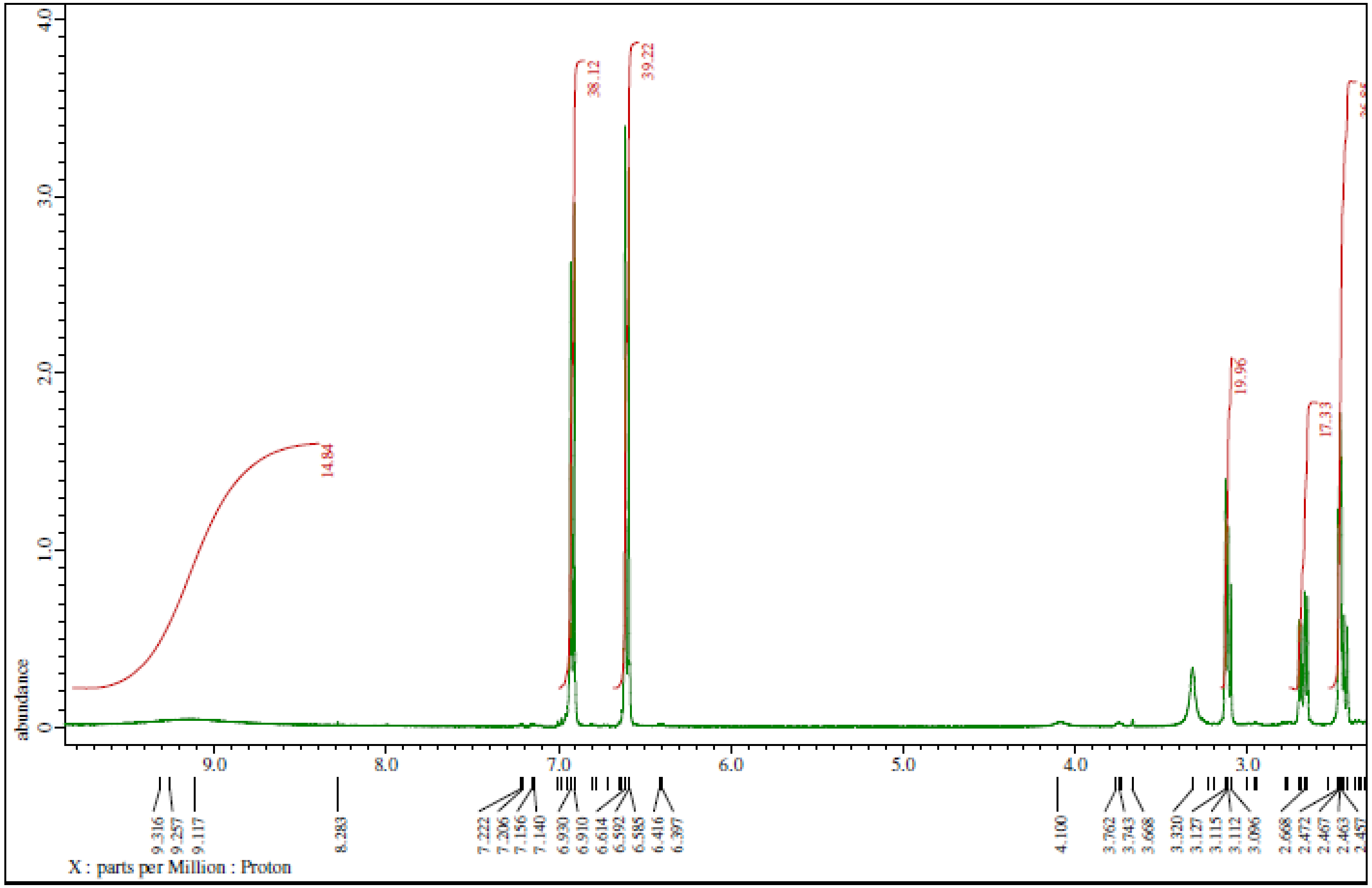

1-Hydroxy-5,6-dimethylpyrazin-2(1*H*)-one **6a**

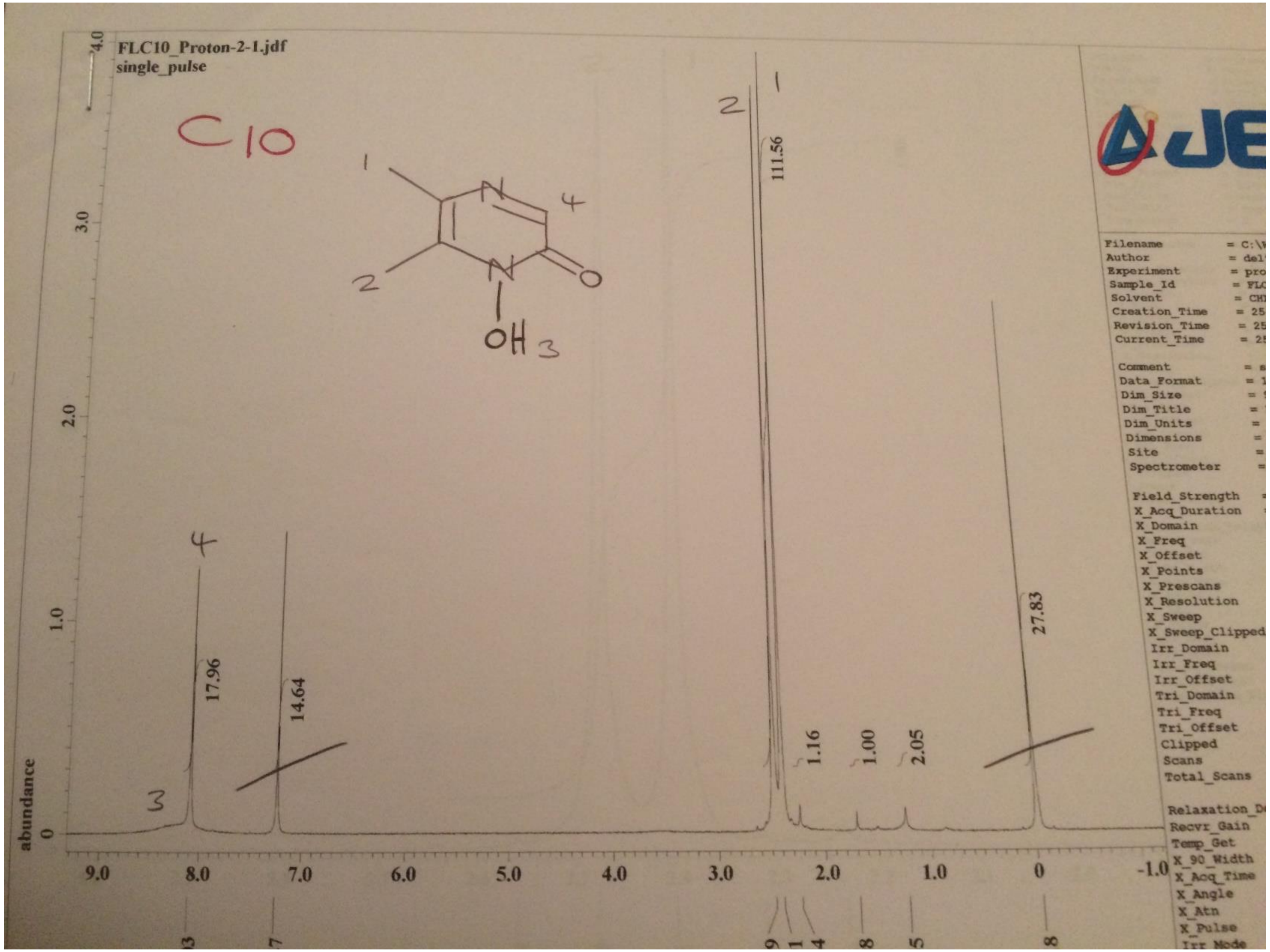

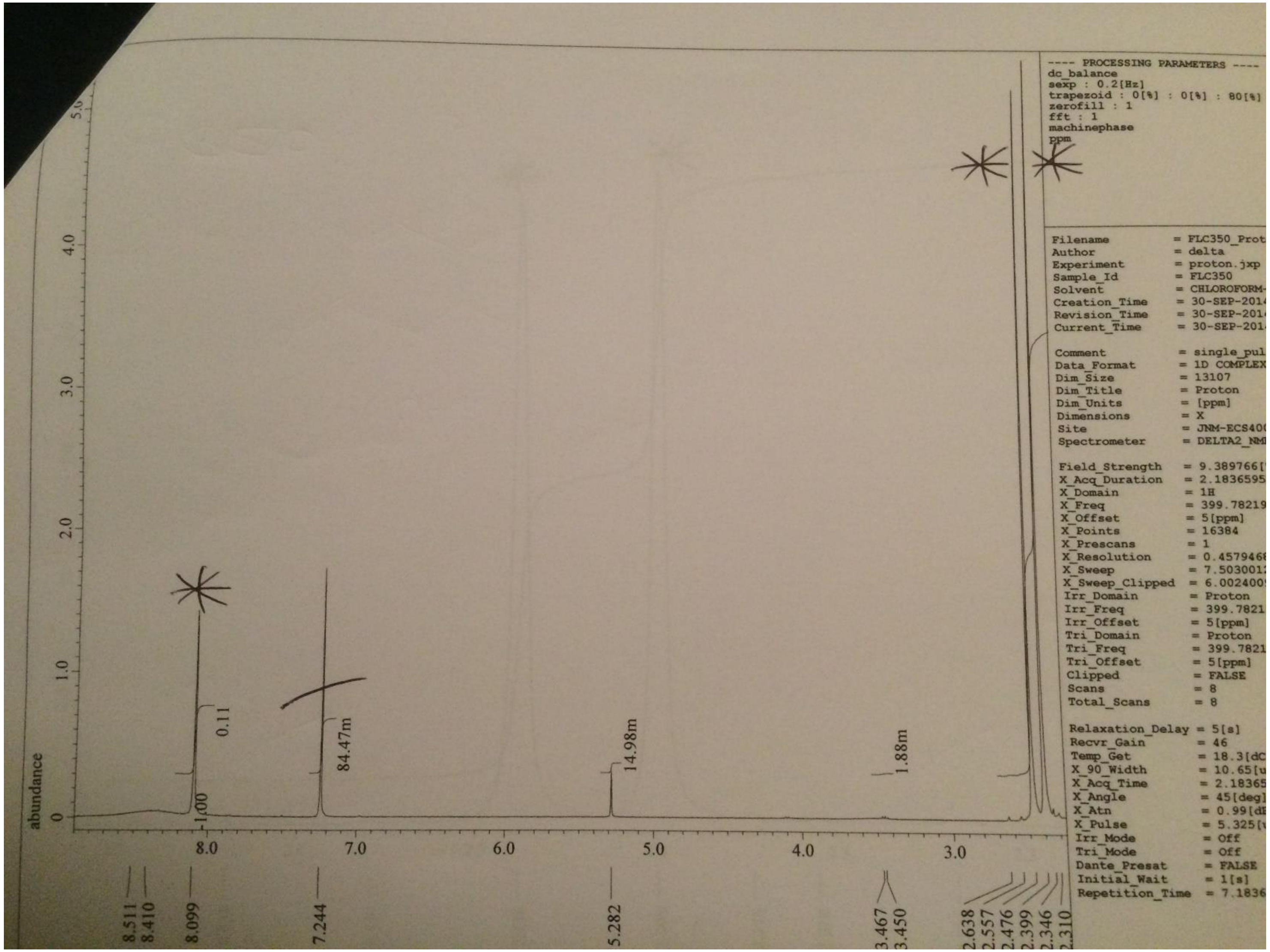
---- PROCESSING PARAMETERS ----
dc_balance
sexp : 0.2[Hz]
trapezoid : 0[%] : 0[%] : 80[%]
zerofill : 1
fft : 1
machinephase
ppm
Filename = FLC350_Prot
Author = delta
Experiment = proton.jxp
Sample_Id = FLC350
Solvent = CHLOROFORM-
Creation_Time = 30-SEP-201
Revision_Time = 30-SEP-201
Current_Time = 30-SEP-201
Comment = single_pul
Data_Format = 1D COMPLEX
Dim_Size = 13107
Dim_Title = Proton
Dim_Units = [ppm]
Dimensions = X
Site = JNM-ECS40
Spectrometer = DELTA2_NM
Field_Strength = 9.389766[
X_Acq_Duration = 2.1836595
X_Domain = 1H
X_Freq = 399.78219
X_Offset = 5[ppm]
X_Points = 16384
X_Prescans = 1
X_Resolution = 0.4579468
X_Sweep = 7.503001
X_Sweep_Clipped = 6.002400
Irr_Domain = Proton
Irr_Freq = 399.7821
Irr_Offset = 5[ppm]
Tri_Domain = Proton
Tri_Freq = 399.7821
Tri_Offset = 5[ppm]
Clipped = FALSE
Scans = 8
Total_Scans = 8
Relaxation_Delay = 5[s]
Recvr_Gain = 46
Temp_Get = 18.3[dC
X_90_Width = 10.65[u
X_Acq_Time = 2.18365
X_Angle = 45[deg]
X_Atn = 0.99[dB
X_Pulse = 5.325[u
Irr_Mode = Off
Tri_Mode = Off
Dante_Presat = FALSE
Initial_Wait = 1[s]
Repetition_Time = 7.1836
abundance
0
1.0
2.0
3.0
4.0
8.0
7.0
6.0
5.0
4.0
3.0
1.00
0.11
84.47m
14.98m
1.88m
8.511
8.410
8.099
7.244
5.282
3.467
3.450
2.638
2.557
2.476
2.399
2.346
2.310

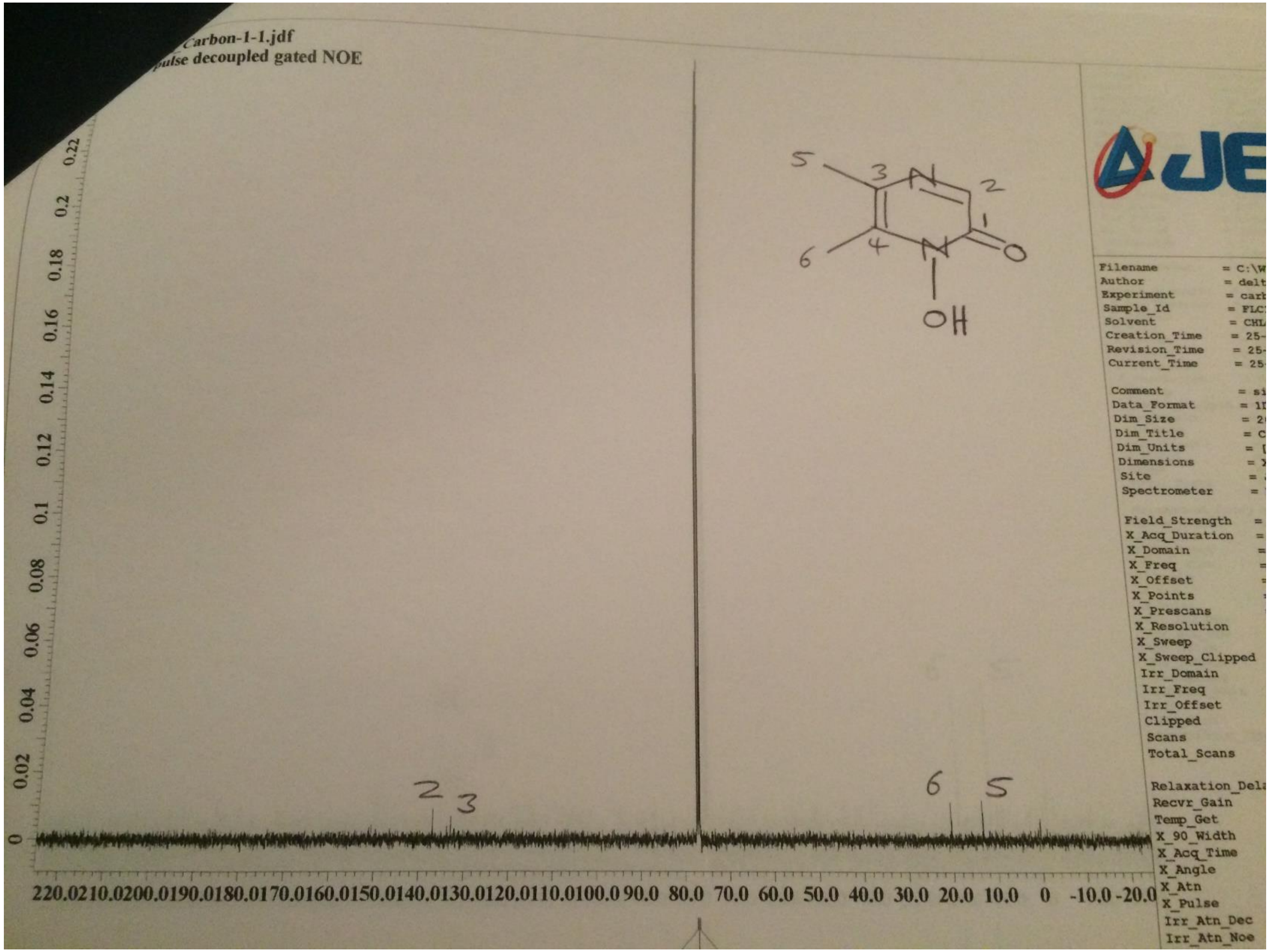
Carbon-1-1.jdf
pulse decoupled gated NOE
Filename
Author
Experiment
Sample_Id
Solvent
Creation_Time
Revision_Time
Current_Time
Comment
Data_Format
Dim_Size
Dim_Title
Dim_Units
Dimensions
Site
Spectrometer
Field_Strength
X_Acq_Duration
X_Domain
X_Freq
X_Offset
X_Points
X_Prescans
X_Resolution
X_Sweep
X_Sweep_Clipped
Irr_Domain
Irr_Freq
Irr_Offset
Clipped
Scans
Total_Scans
Relaxation_Dela
Recvr_Gain
Temp_Get
X_90_Width
X_Acq_Time
X_Angle
X_Atn
X_Pulse
Irr_Atn_Dec
Irr_Atn_Noe
220.0 210.0 200.0 190.0 180.0 170.0 160.0 150.0 140.0 130.0 120.0 110.0 100.0 90.0 80.0 70.0 60.0 50.0 40.0 30.0 20.0 10.0 0 -10.0 -20.0
0 0.02 0.04 0.06 0.08 0.1 0.12 0.14 0.16 0.18 0.2 0.22

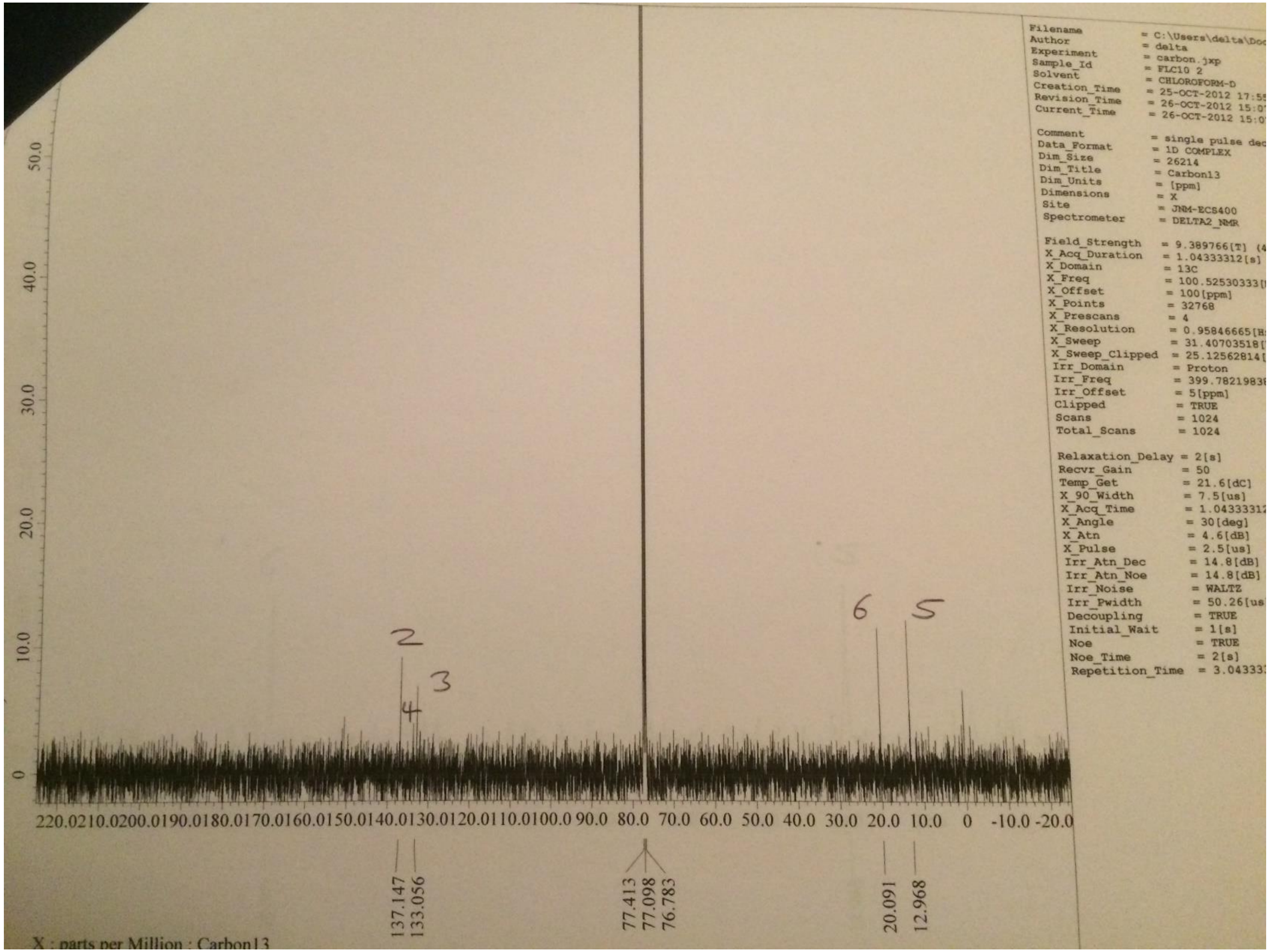
Filename = C:\Users\delta\Doc
Author = delta
Experiment = carbon.jxp
Sample_Id = FLC10 2
Solvent = CHLOROFORM-D
Creation_Time = 25-OCT-2012 17:5
Revision_Time = 26-OCT-2012 15:0
Current_Time = 26-OCT-2012 15:0
Comment = single pulse dec
Data_Format = 1D COMPLEX
Dim_Size = 26214
Dim_Title = Carbon13
Dim_Units = [ppm]
Dimensions = X
Site = JNM-ECS400
Spectrometer = DELTA2_NMR
Field_Strength = 9.389766[T]
X_Acq_Duration = 1.04333312[s]
X_Domain = 13C
X_Freq = 100.52530333
X_Offset = 100[ppm]
X_Points = 32768
X_Prescans = 4
X_Resolution = 0.95846665
X_Sweep = 31.40703518
X_Sweep_Clipped = 25.12562814
Irr_Domain = Proton
Irr_Freq = 399.7821983
Irr_Offset = 5[ppm]
Clipped = TRUE
Scans = 1024
Total_Scans = 1024
Relaxation_Delay = 2[s]
Recvr_Gain = 50
Temp_Get = 21.6[dC]
X_90_Width = 7.5[us]
X_Acq_Time = 1.04333312
X_Angle = 30[deg]
X_Atn = 4.6[dB]
X_Pulse = 2.5[us]
Irr_Atn_Dec = 14.8[dB]
Irr_Atn_Noe = 14.8[dB]
Irr_Noise = WALTZ
Irr_Pwidth = 50.26[us
Decoupling = TRUE
Initial_Wait = 1[s]
Noe = TRUE
Noe_Time = 2[s]
Repetition_Time = 3.04333
2
3
4
6
5
137.147
133.056
77.413
77.098
76.783
20.091
12.968
X : parts per Million : Carbon13

1-Hydroxy-3-benzyl-5,6-dimethylpyrazin-2(1*H*)-one **6c**

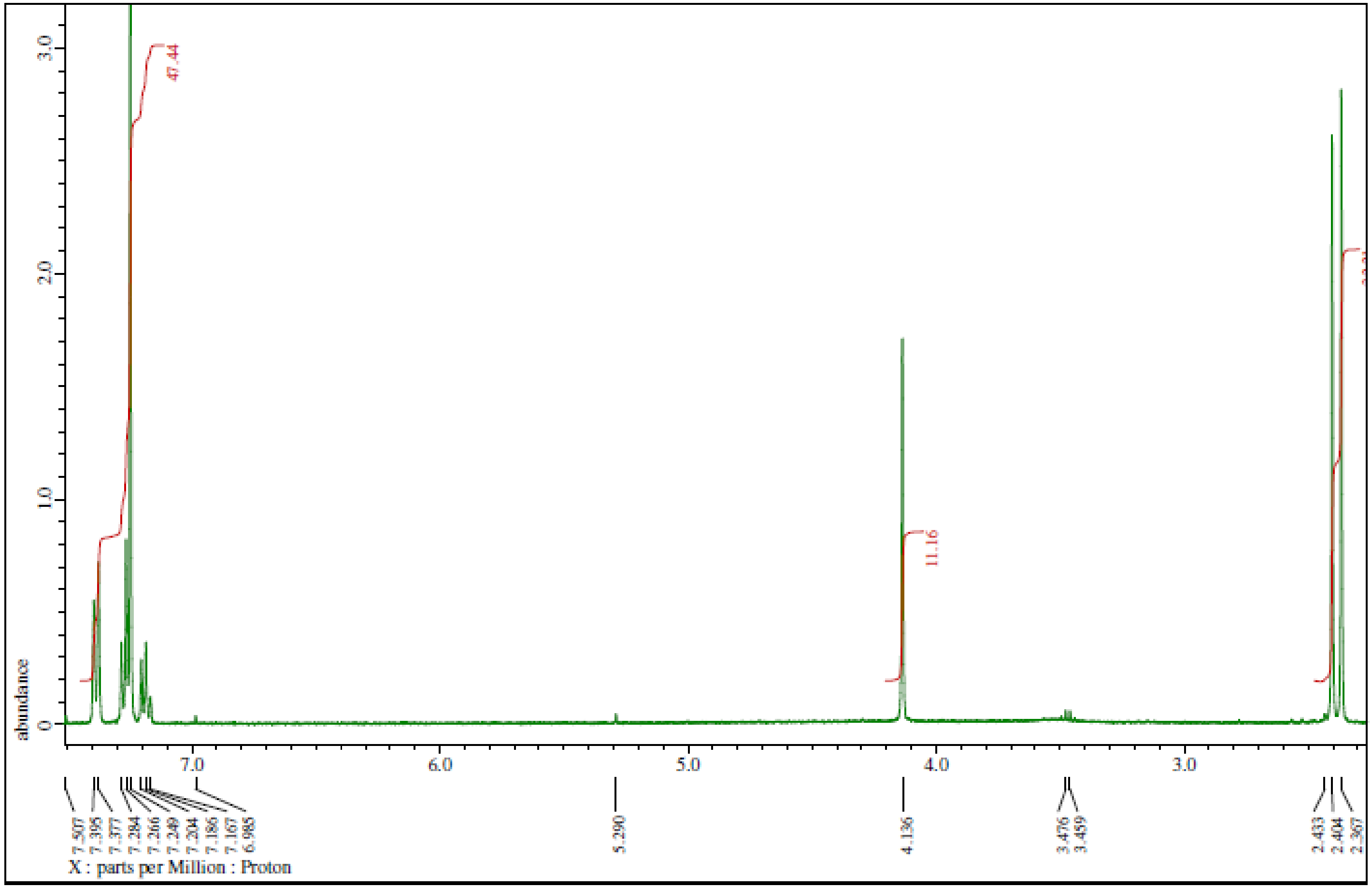

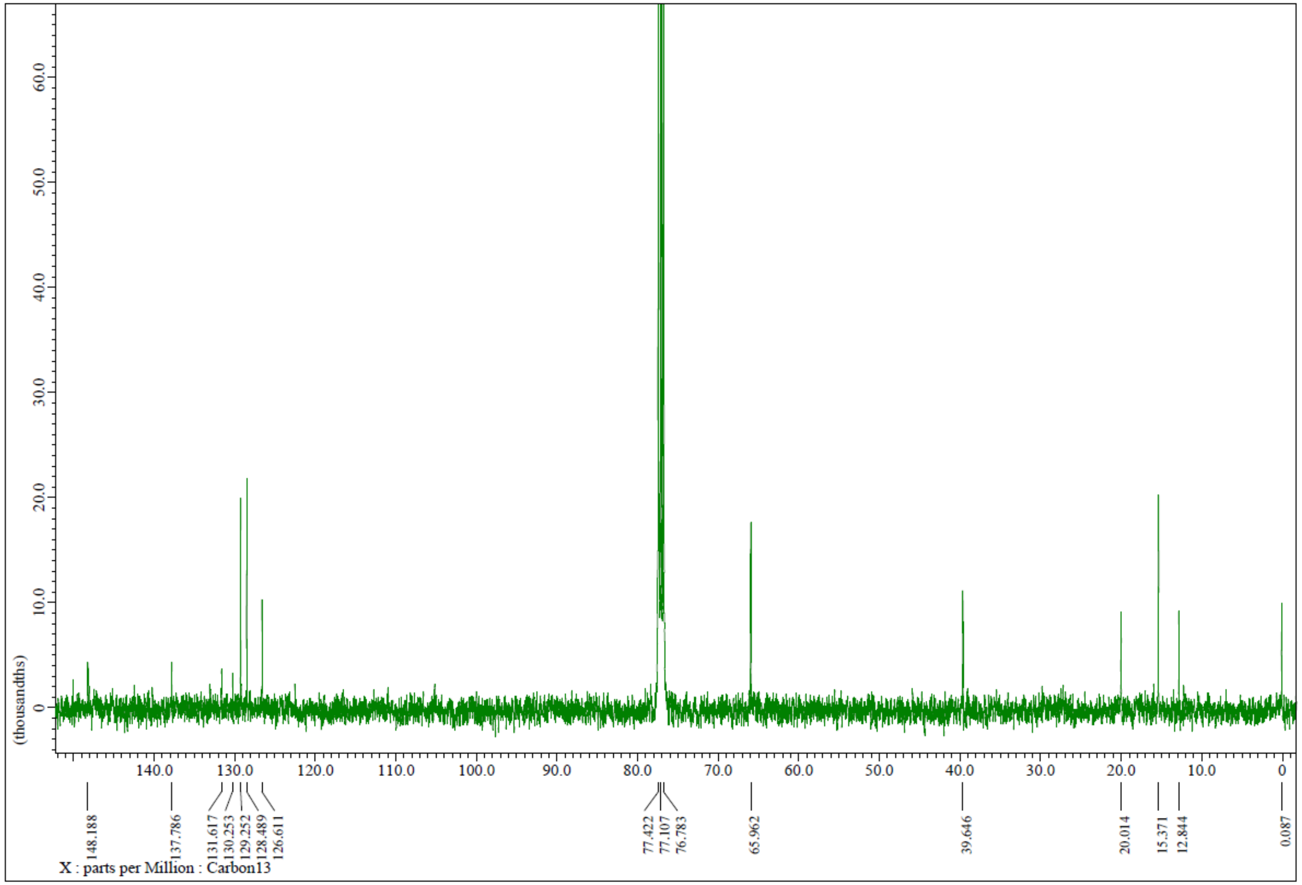
(thousandths)
0
10.0
20.0
30.0
40.0
50.0
60.0
140.0
130.0
120.0
110.0
100.0
90.0
80.0
70.0
60.0
50.0
40.0
30.0
20.0
10.0
0
148.188
137.786
131.617
130.253
129.252
128.489
126.611
77.422
77.107
76.783
65.962
39.646
20.014
15.371
12.844
0.087
X : parts per Million : Carbon13

1-Hydroxy-3-(4-hydroxybenzyl)-5,6-dimethylpyrazin-2(1*H*)-one **6d**

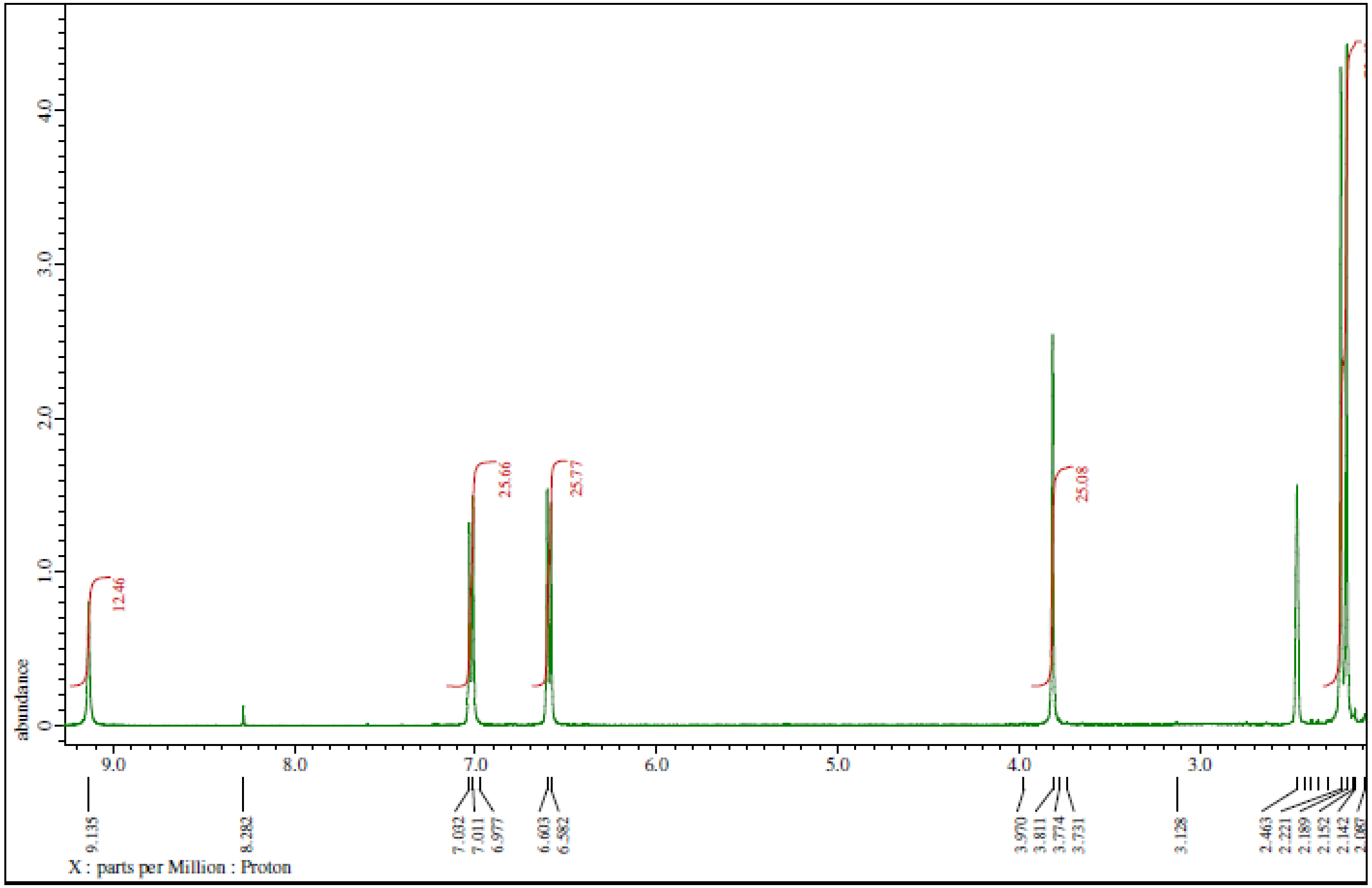

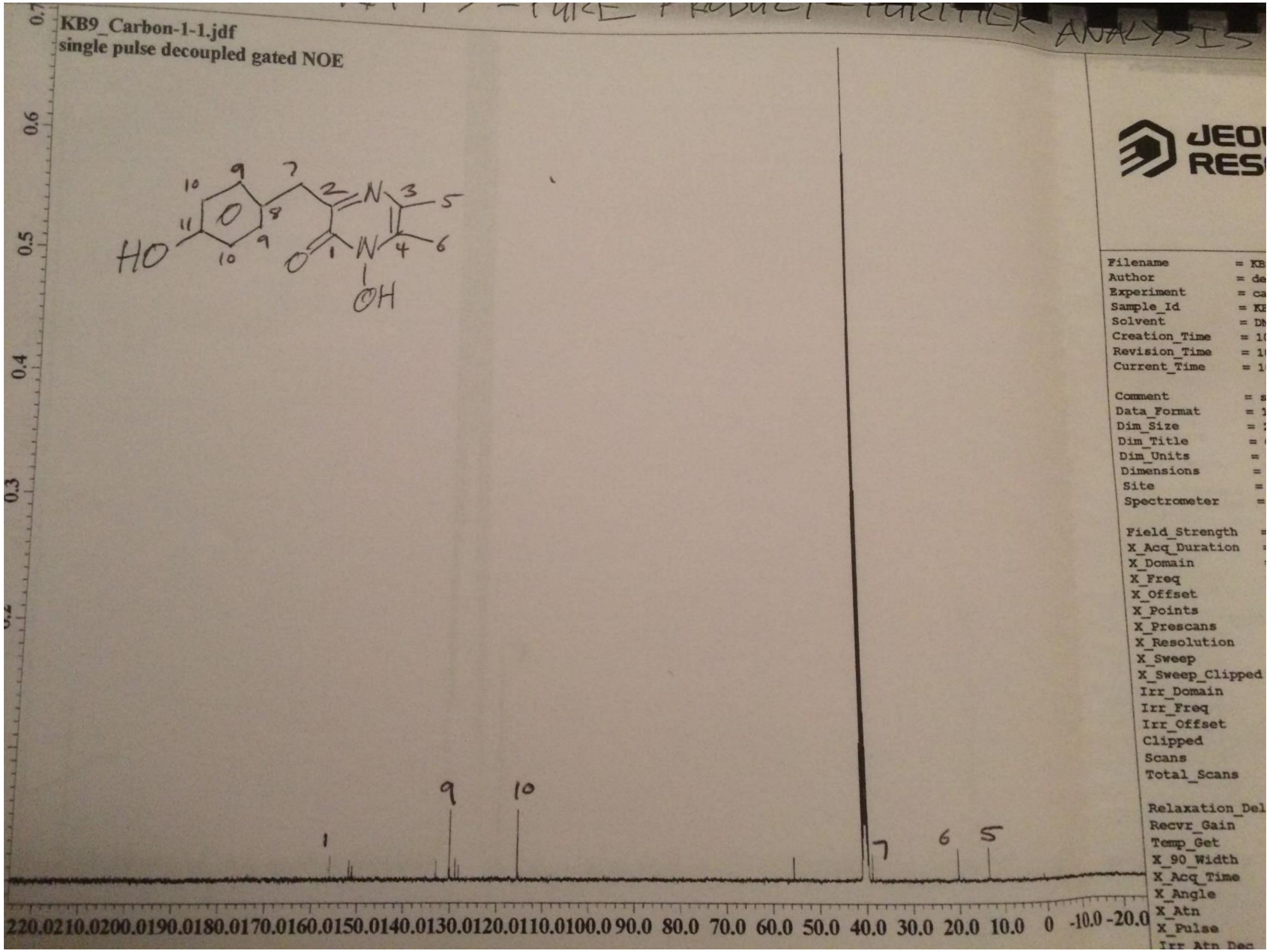

KB9_Carbon-1-1.jdf
single pulse decoupled gated NOE
HO
OH
Filename
Author
Experiment
Sample_Id
Solvent
Creation_Time
Revision_Time
Current_Time
Comment
Data_Format
Dim_Size
Dim_Title
Dim_Units
Dimensions
Site
Spectrometer
Field_Strength
X_Acq_Duration
X_Domain
X_Freq
X_Offset
X_Points
X_Prescans
X_Resolution
X_Sweep
X_Sweep_Clipped
Irr_Domain
Irr_Freq
Irr_Offset
Clipped
Scans
Total_Scans
Relaxation_Del
Recvr_Gain
Temp_Get
X_90_Width
X_Acq_Time
X_Angle
X_Atn
X_Pulse
220.0 210.0 200.0 190.0 180.0 170.0 160.0 150.0 140.0 130.0 120.0 110.0 100.0 90.0 80.0 70.0 60.0 50.0 40.0 30.0 20.0 10.0 0 -10.0 -20.0

N-Boc glycine hydroxamic acid benzyl ester **8a**

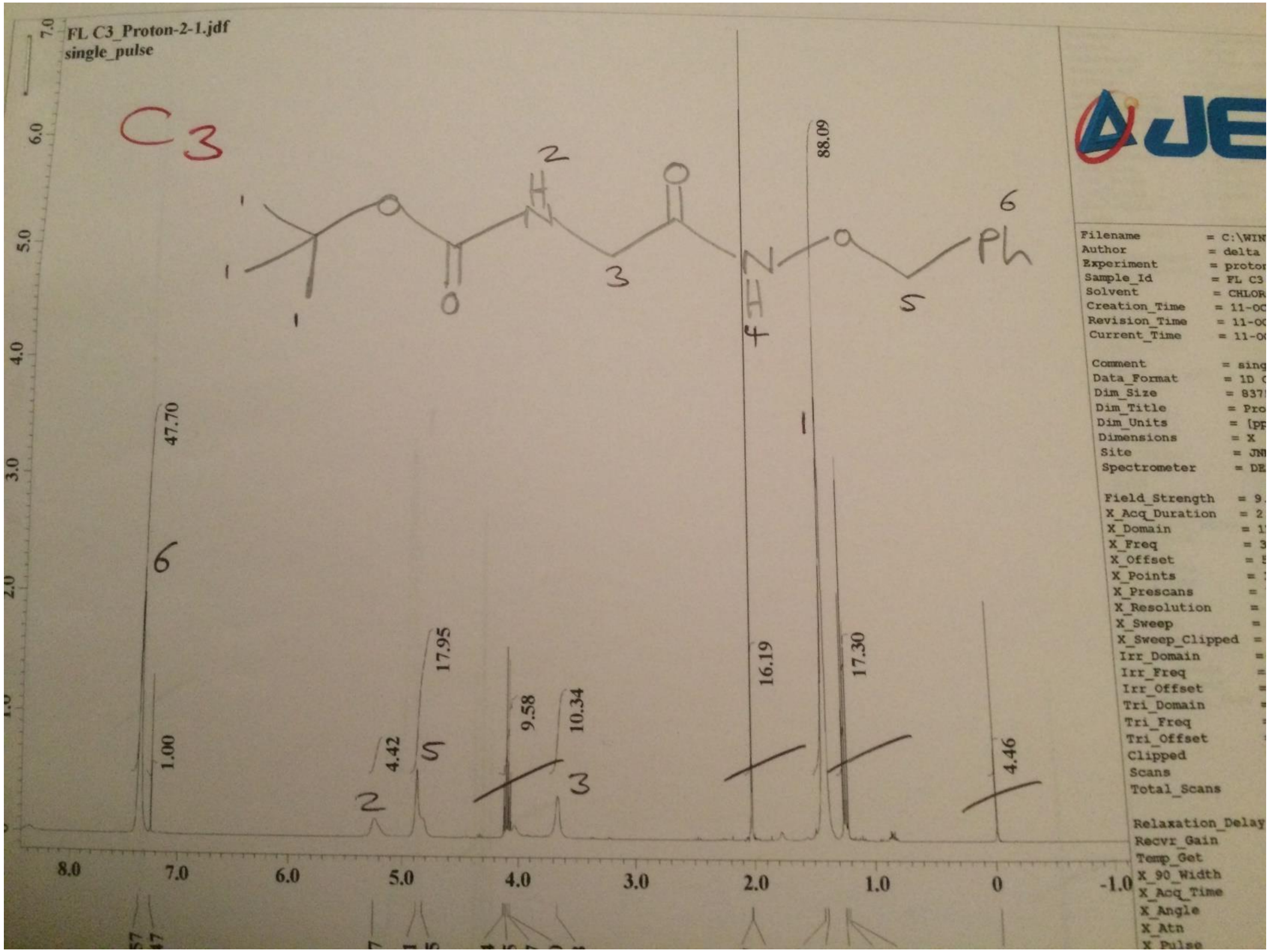

N-Boc alanine hydroxamic acid benzyl ester **8b**

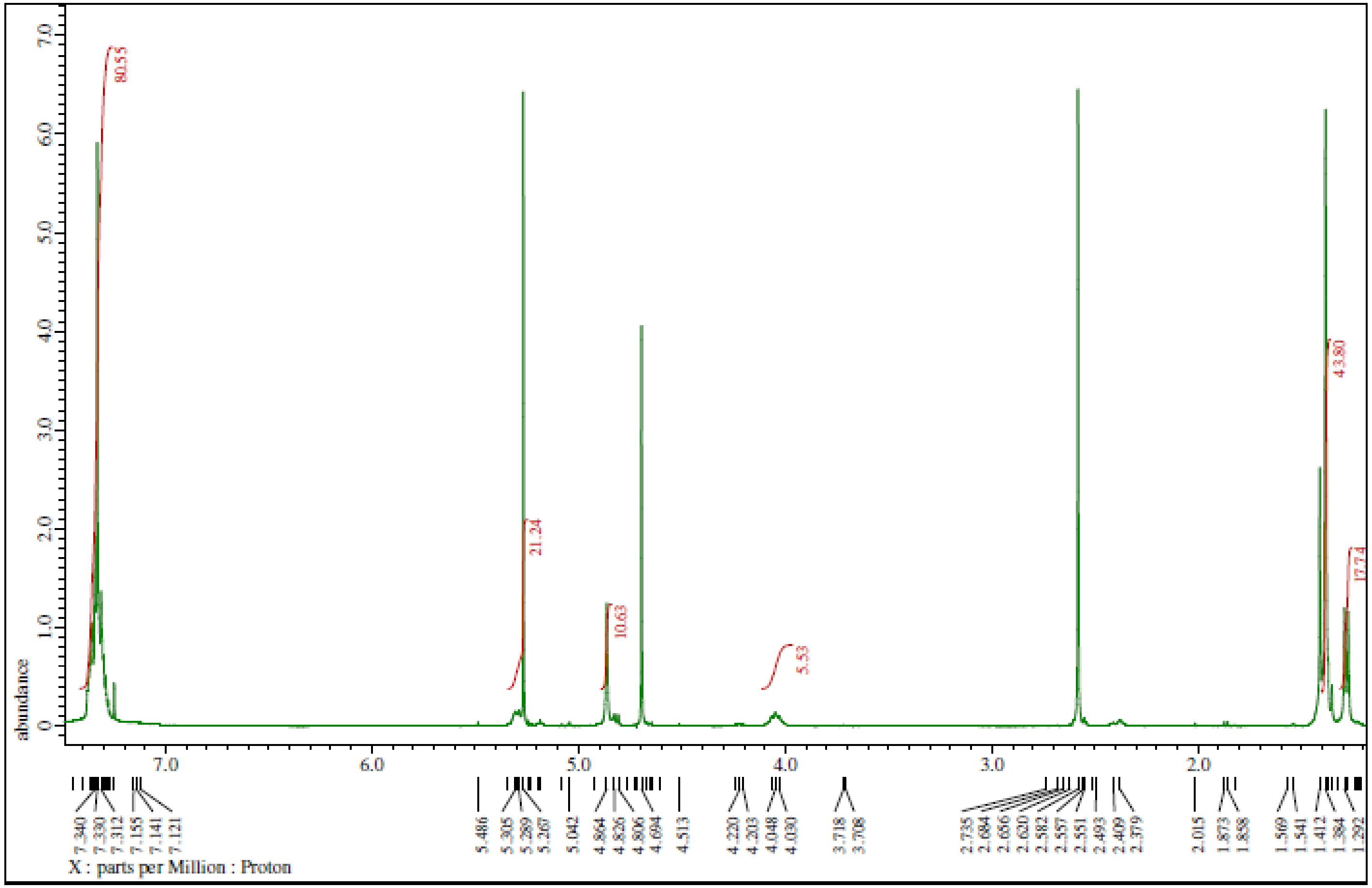

N-Boc phenylalanine hydroxamic acid benzyl ester **8c**

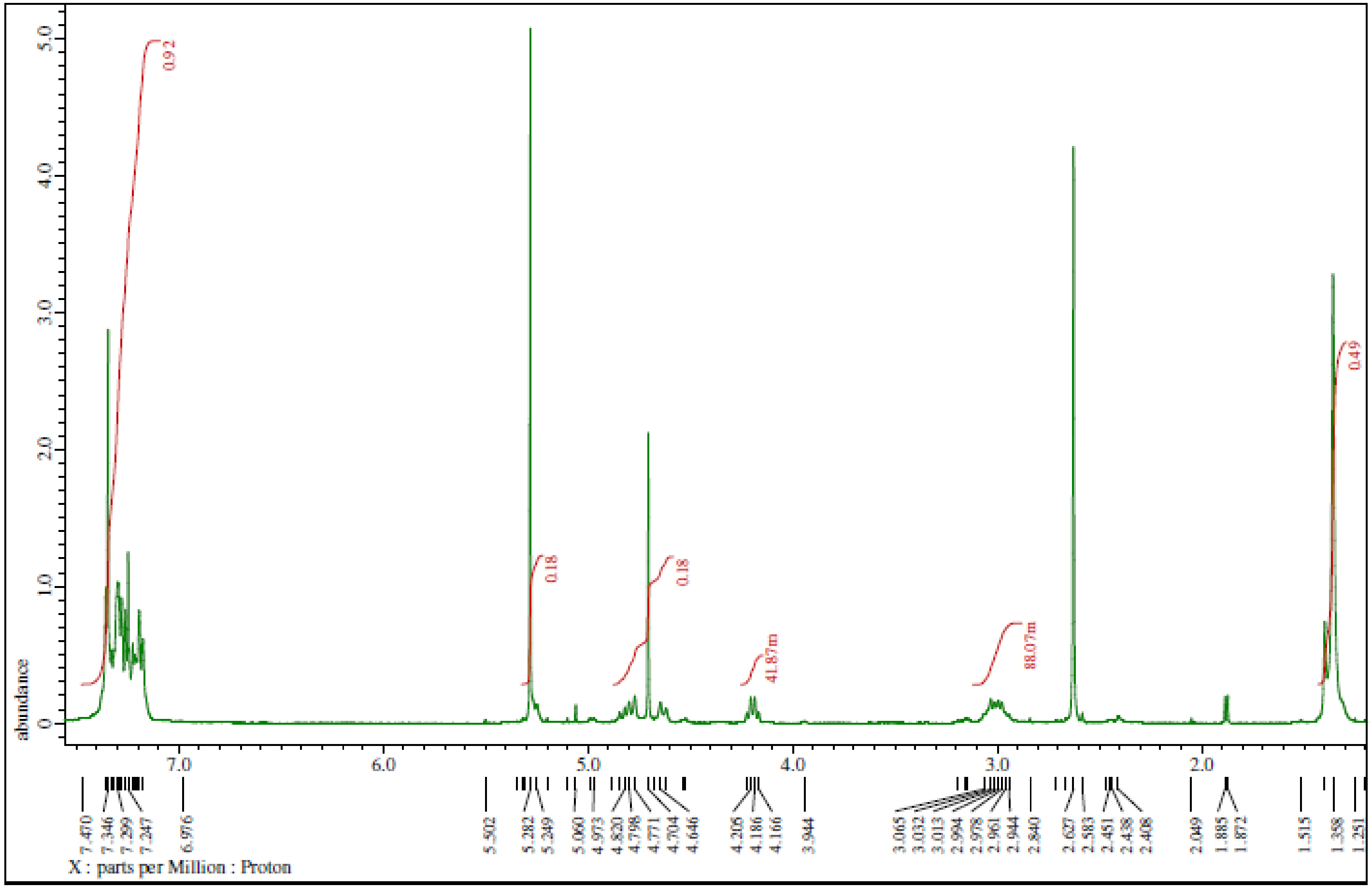

N-Boc valine hydroxamic acid benzyl ester **8e**

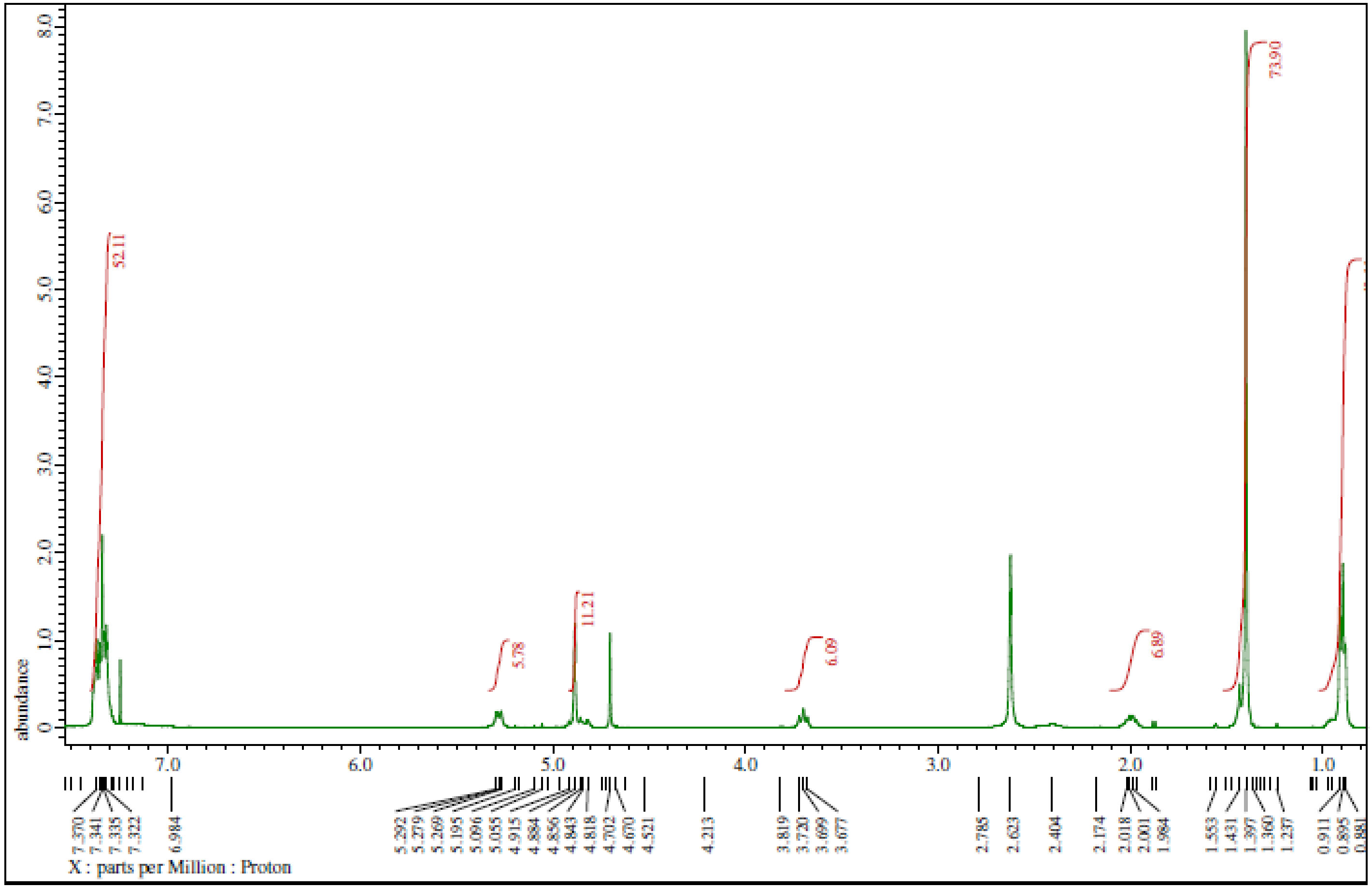

N-Boc leucine hydroxamic acid benzyl ester **8f**

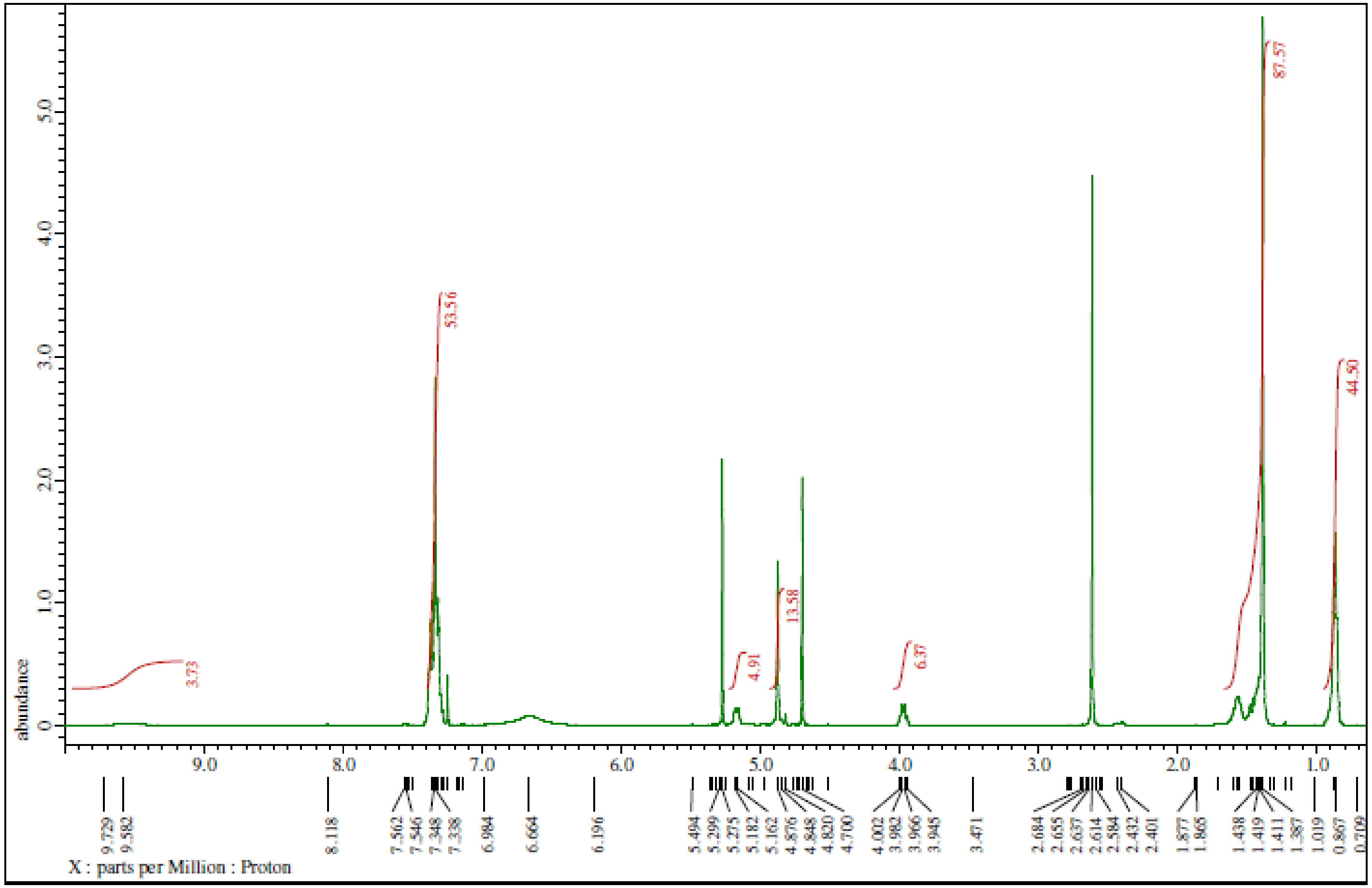

Alanine hydroxamic acid benzyl ester TFA salt **9b**

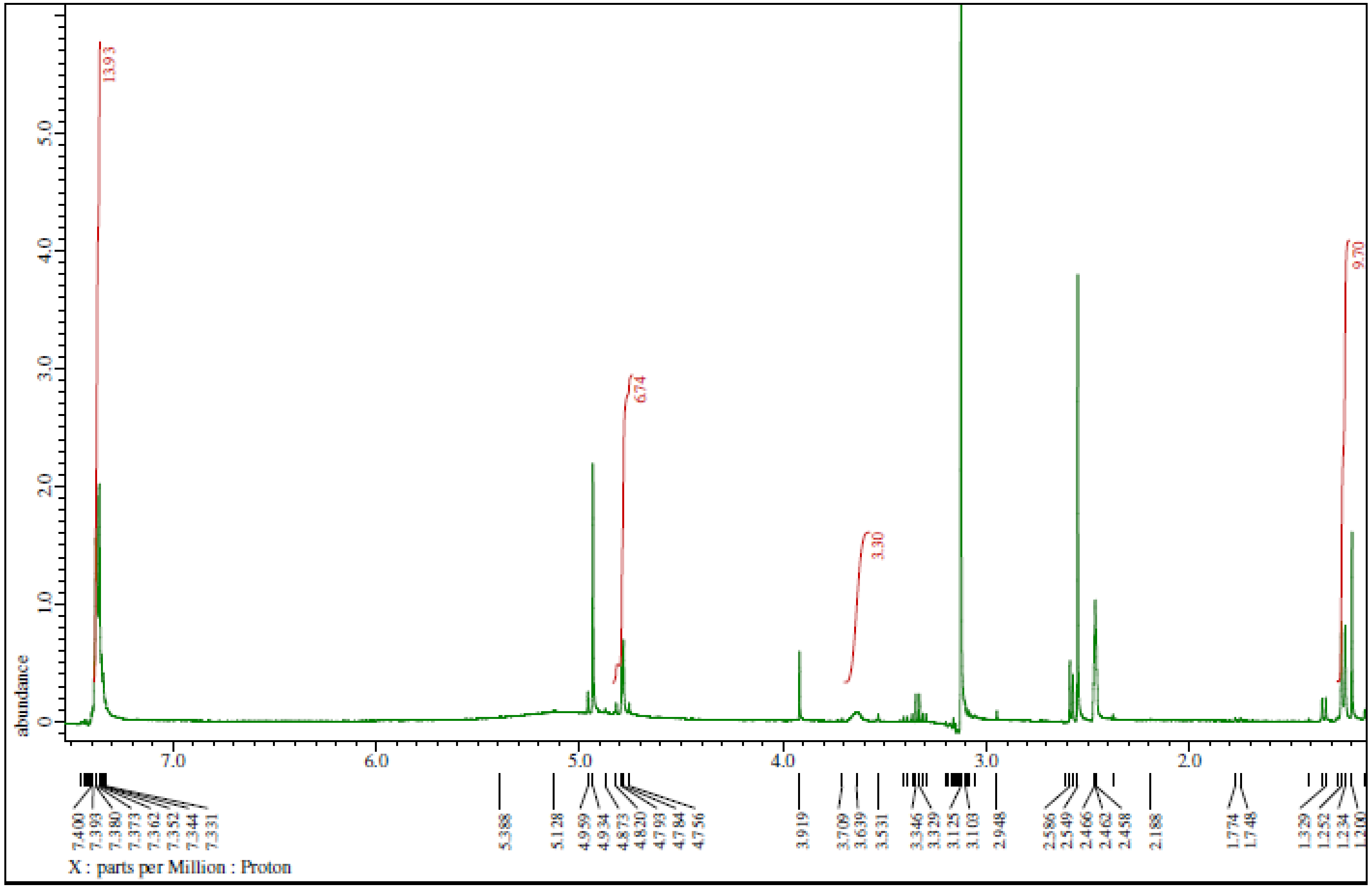

Phenylalanine hydroxamic acid benzyl ester TFA salt **9c**

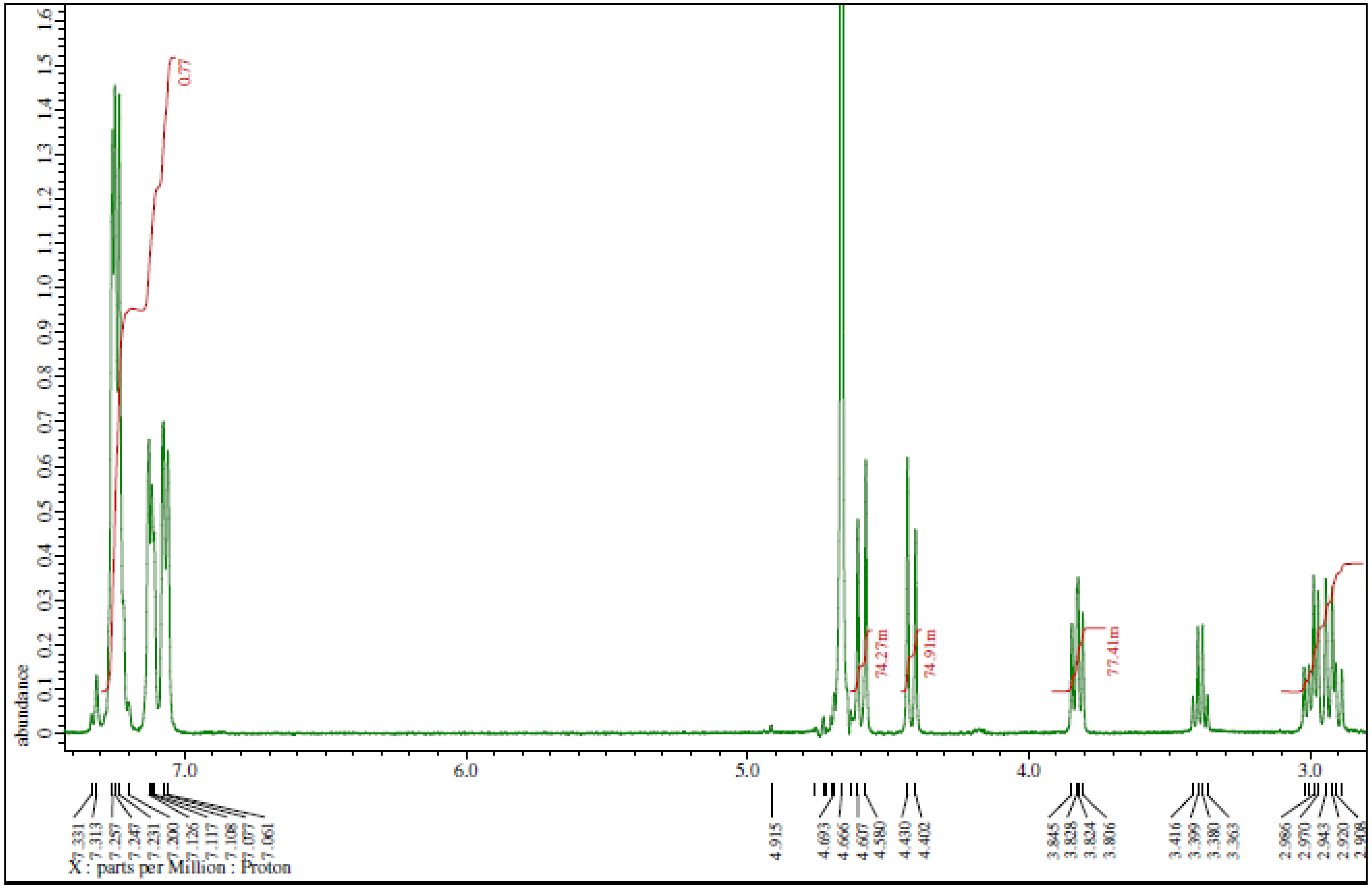

Valine hydroxamic acid benzyl ester TFA salt **9e**

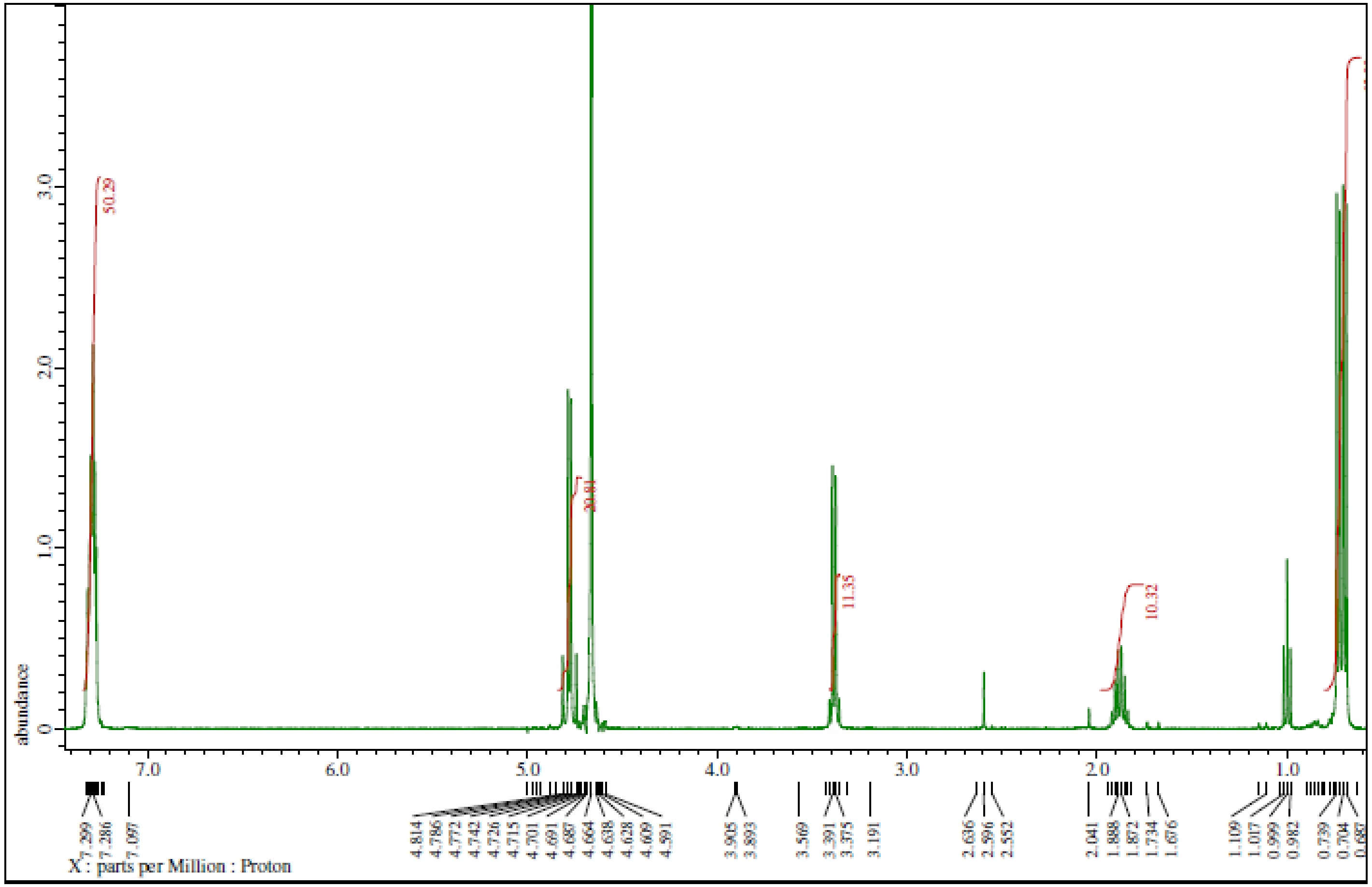

Leucine hydroxamic acid benzyl ester TFA salt **9f**

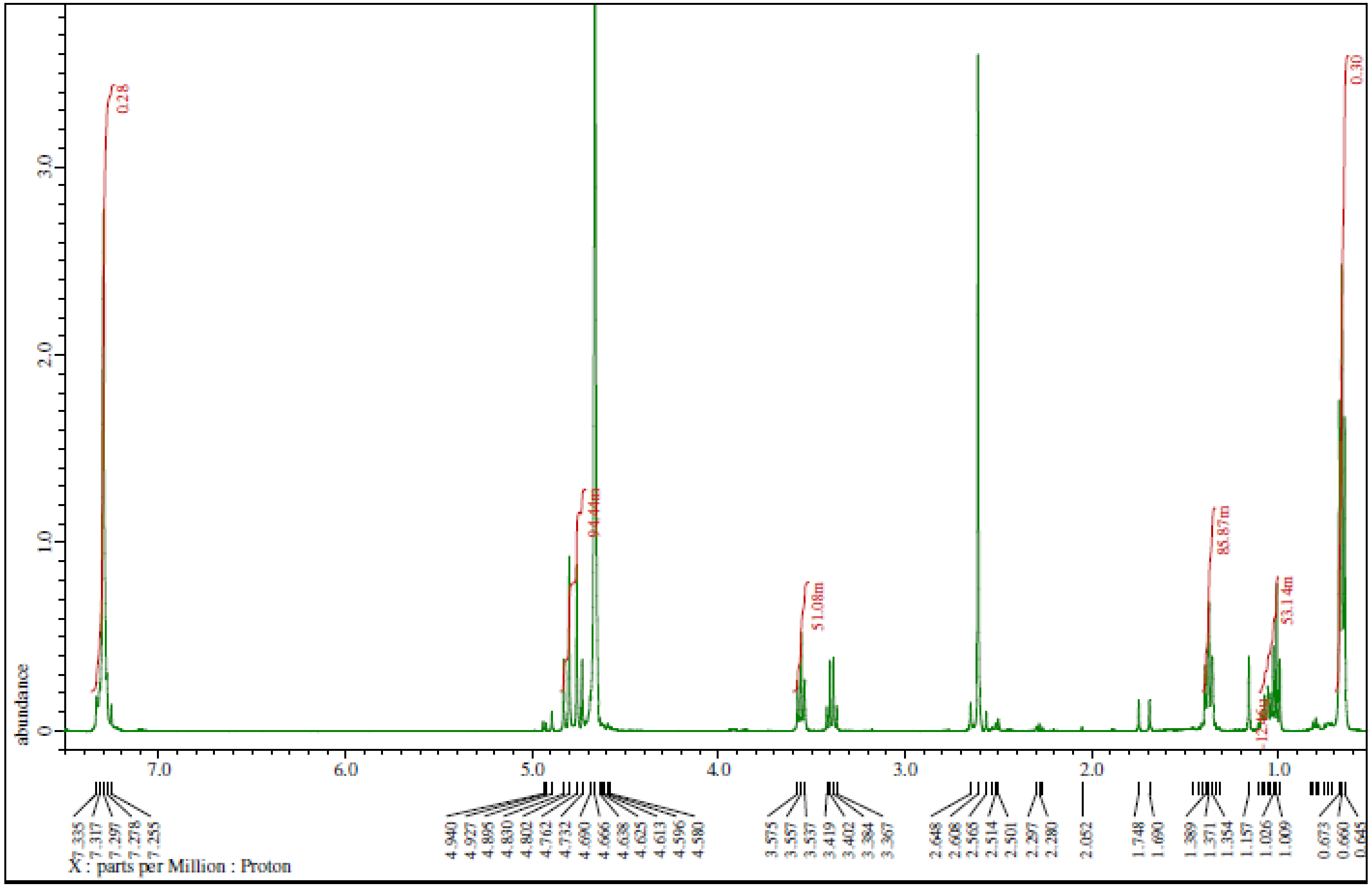

1-Hydroxy-6-phenylpyrazin-2(1*H*)-one **10a**

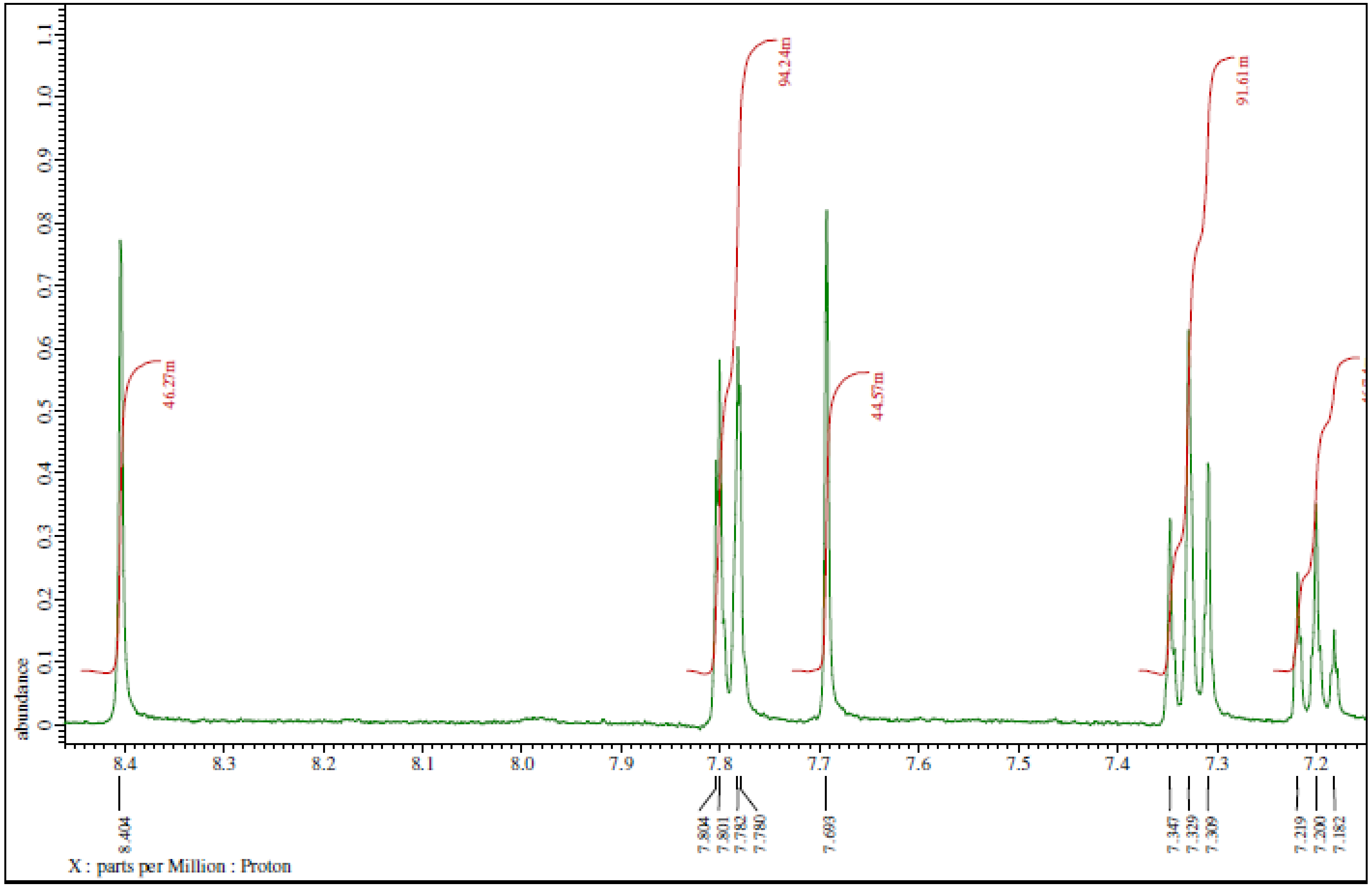

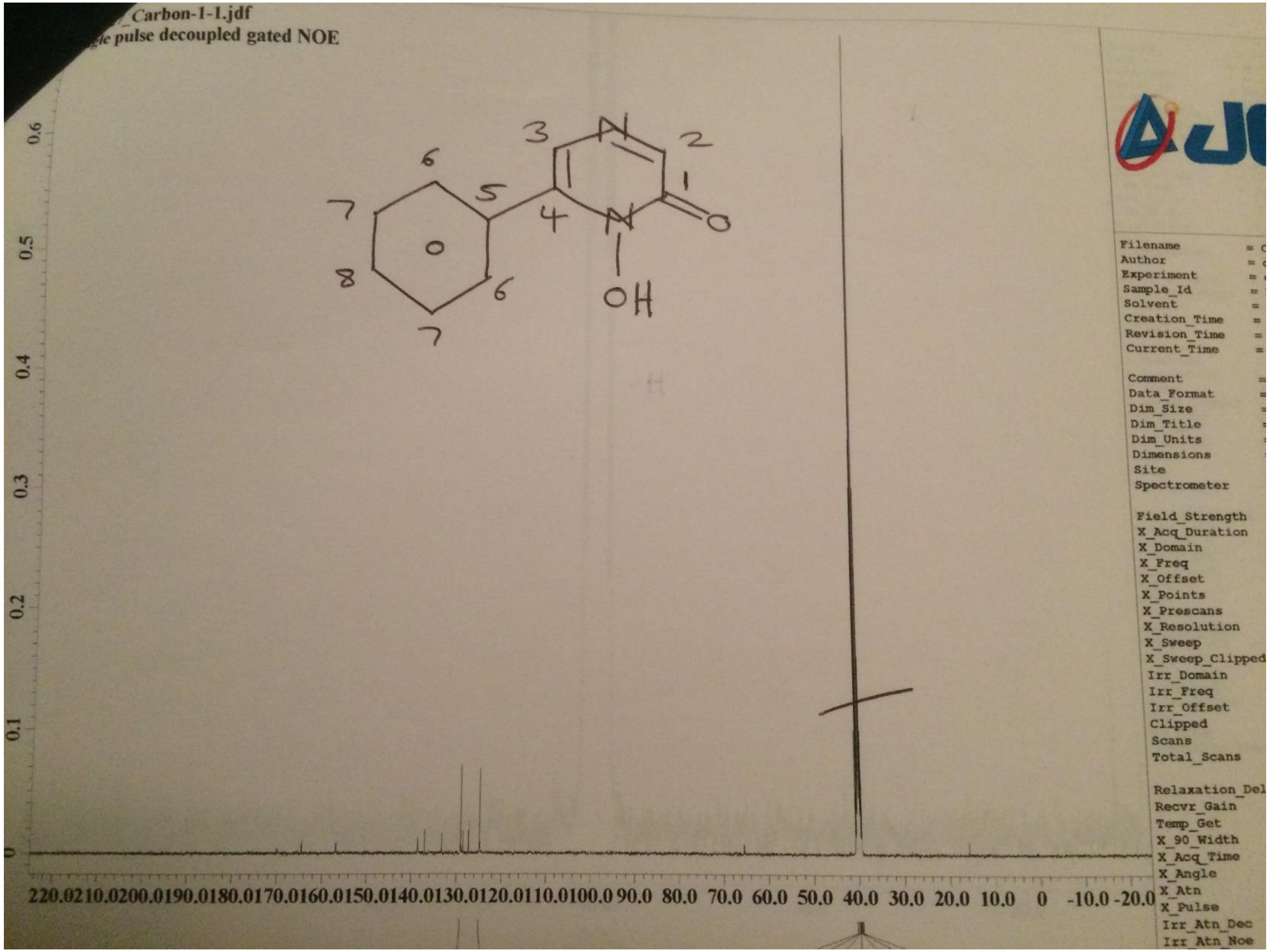

_Carbon-1-1.jdf
gle pulse decoupled gated NOE
0.6
0.5
0.4
0.3
0.2
0.1
0
220.0 210.0 200.0 190.0 180.0 170.0 160.0 150.0 140.0 130.0 120.0 110.0 100.0 90.0 80.0 70.0 60.0 50.0 40.0 30.0 20.0 10.0 0 -10.0 -20.0
Filename
Author
Experiment
Sample_Id
Solvent
Creation_Time
Revision_Time
Current_Time
Comment
Data_Format
Dim_Size
Dim_Title
Dim_Units
Dimensions
Site
Spectrometer
Field_Strength
X_Acq_Duration
X_Domain
X_Freq
X_Offset
X_Points
X_Prescans
X_Resolution
X_Sweep
X_Sweep_Clipped
Irr_Domain
Irr_Freq
Irr_Offset
Clipped
Scans
Total_Scans
Relaxation_Del
Recvr_Gain
Temp_Get
X_90_Width
X_Acq_Time
X_Angle
X_Atn
X_Pulse
Irr_Atn_Dec
Irr_Atn_Noe

1-Hydroxy-6-(4-methoxyphenyl)-pyrazin-2(1*H*)-one **10b**

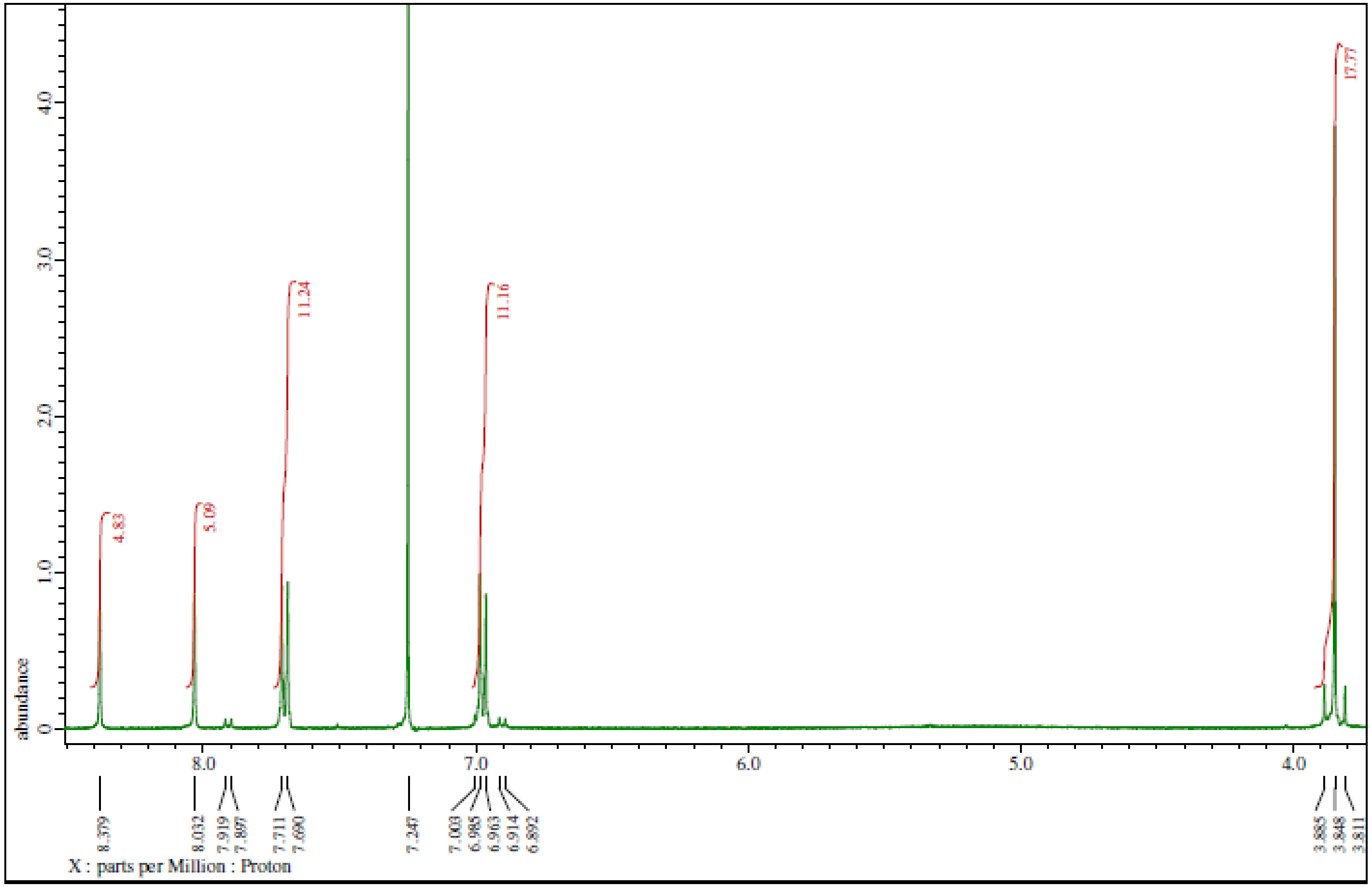

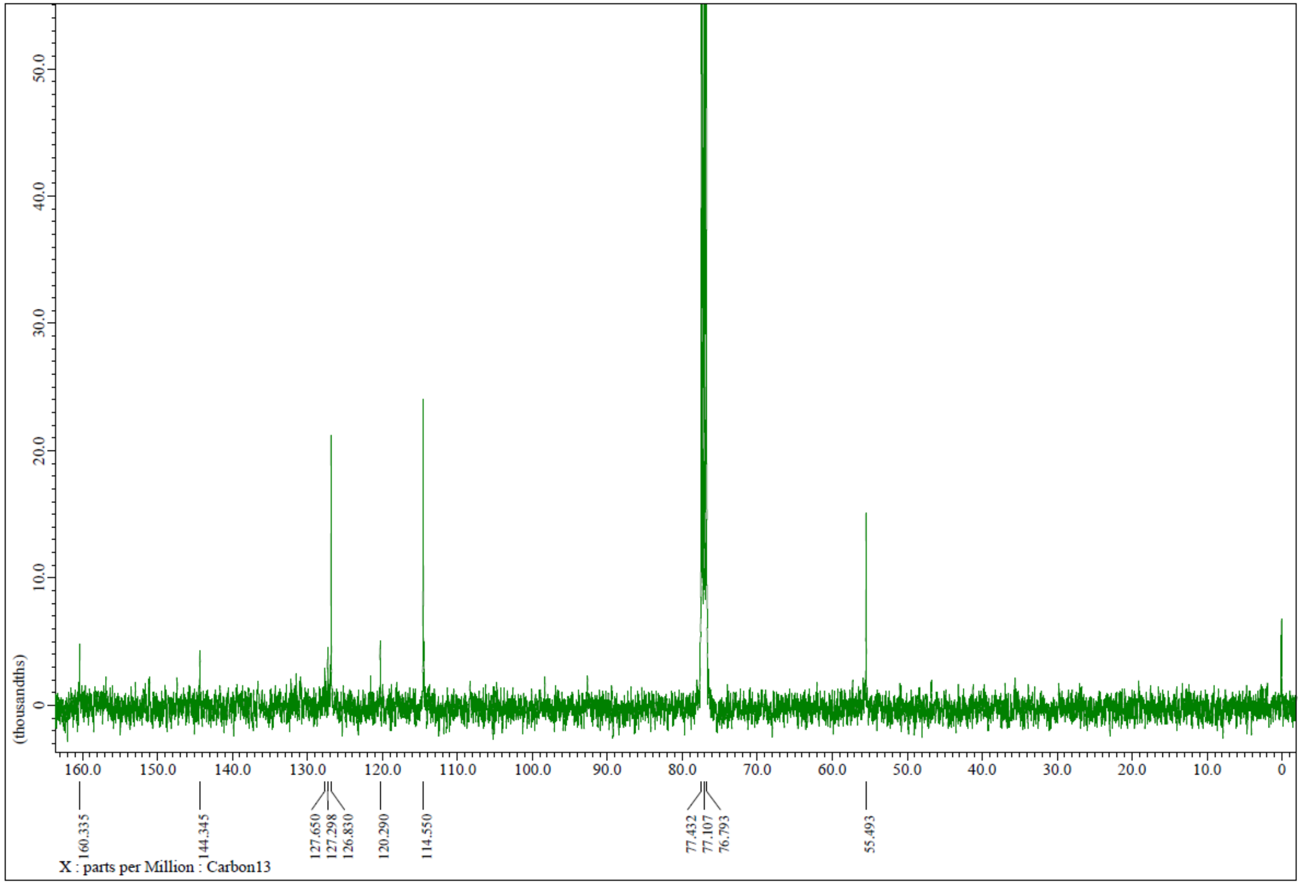

(thousandths)
50.0
40.0
30.0
20.0
10.0
0
160.0
150.0
140.0
130.0
120.0
110.0
100.0
90.0
80.0
70.0
60.0
50.0
40.0
30.0
20.0
10.0
0
160.335
144.345
127.650
127.298
126.830
120.290
114.550
77.432
77.107
76.793
55.493
X : parts per Million : Carbon13

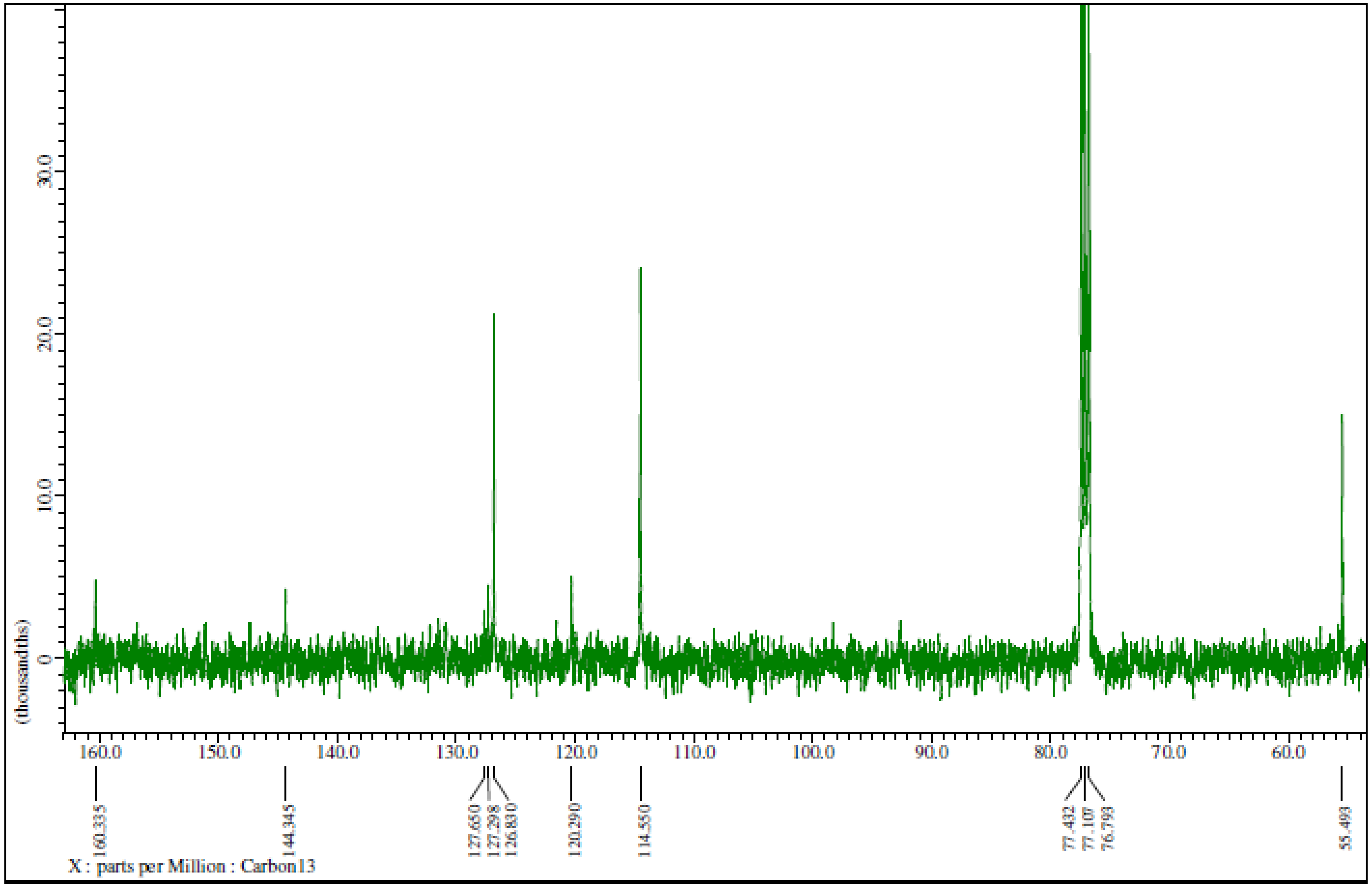

30.0
20.0
10.0
0
(thousandths)
160.0
150.0
140.0
130.0
120.0
110.0
100.0
90.0
80.0
70.0
60.0
160.335
144.345
127.650
127.298
126.830
120.290
114.550
77.432
77.107
76.793
55.493
X : parts per Million : Carbon13

1-Hydroxy-6-(4-fluorophenyl)-pyrazin-2(1*H*)-one **10c**

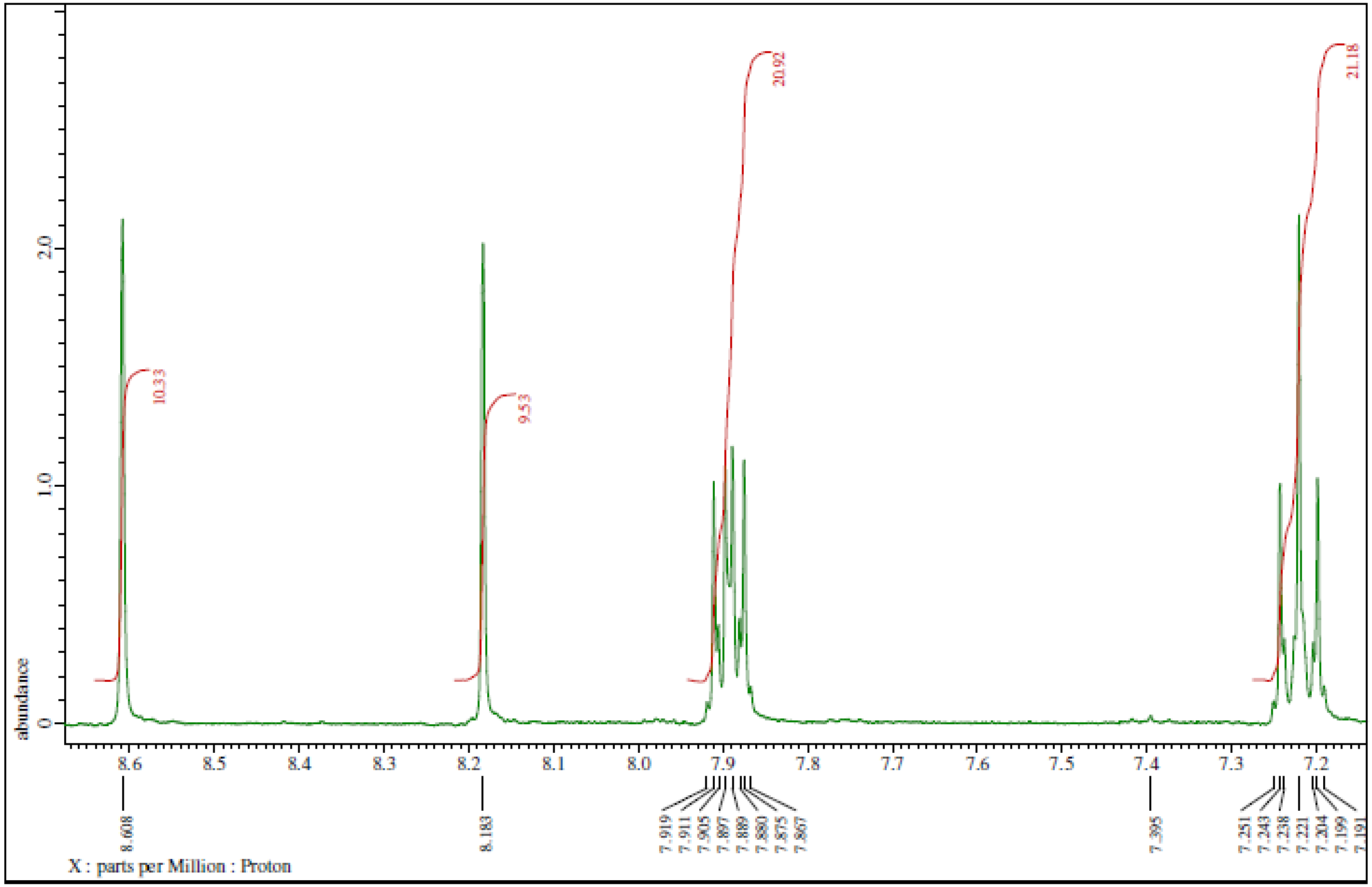

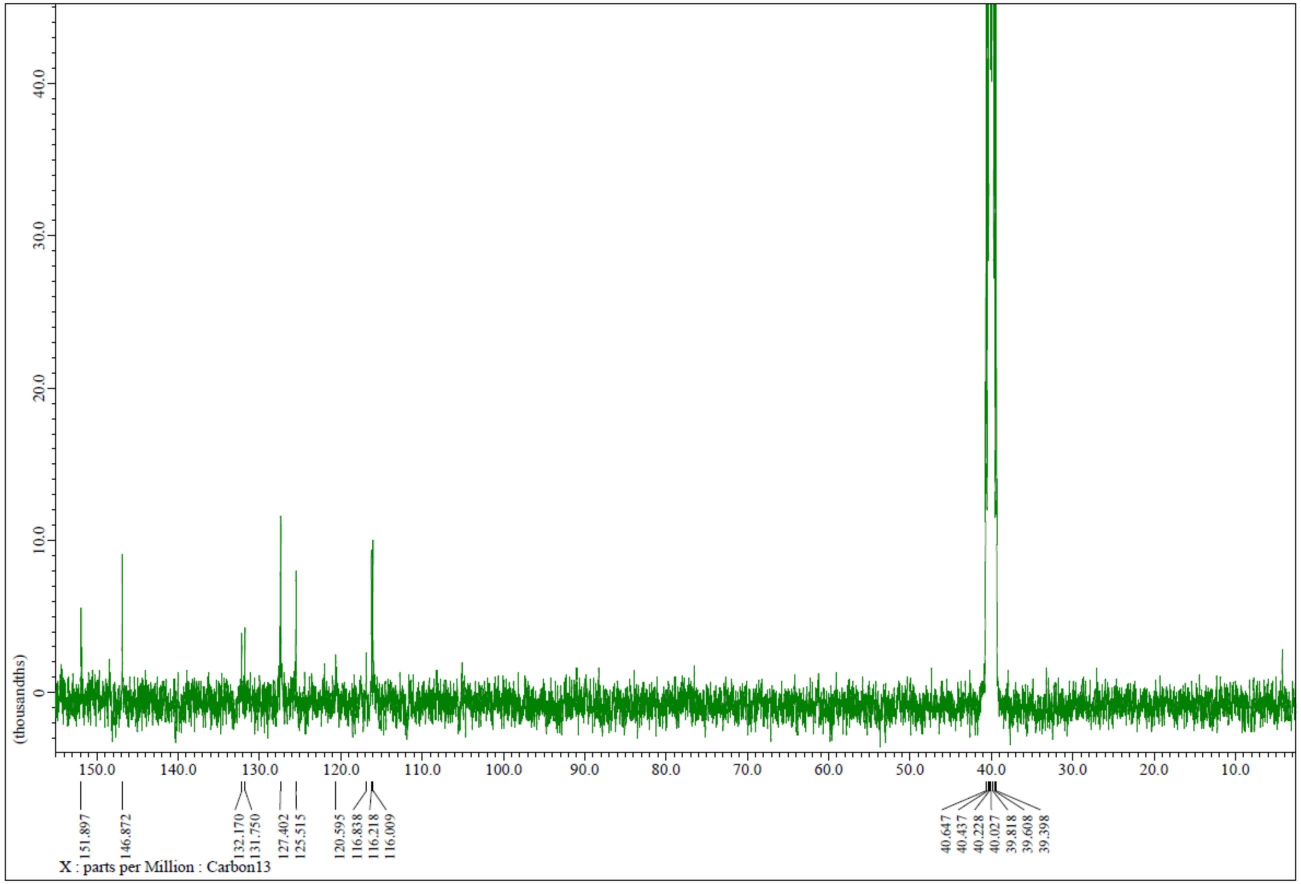

(thousandths)
40.0
30.0
20.0
10.0
0
150.0
140.0
130.0
120.0
110.0
100.0
90.0
80.0
70.0
60.0
50.0
40.0
30.0
20.0
10.0
151.897
146.872
132.170
131.750
127.402
125.515
120.595
116.838
116.218
116.009
40.647
40.437
40.228
40.027
39.818
39.608
39.398
X : parts per Million : Carbon13

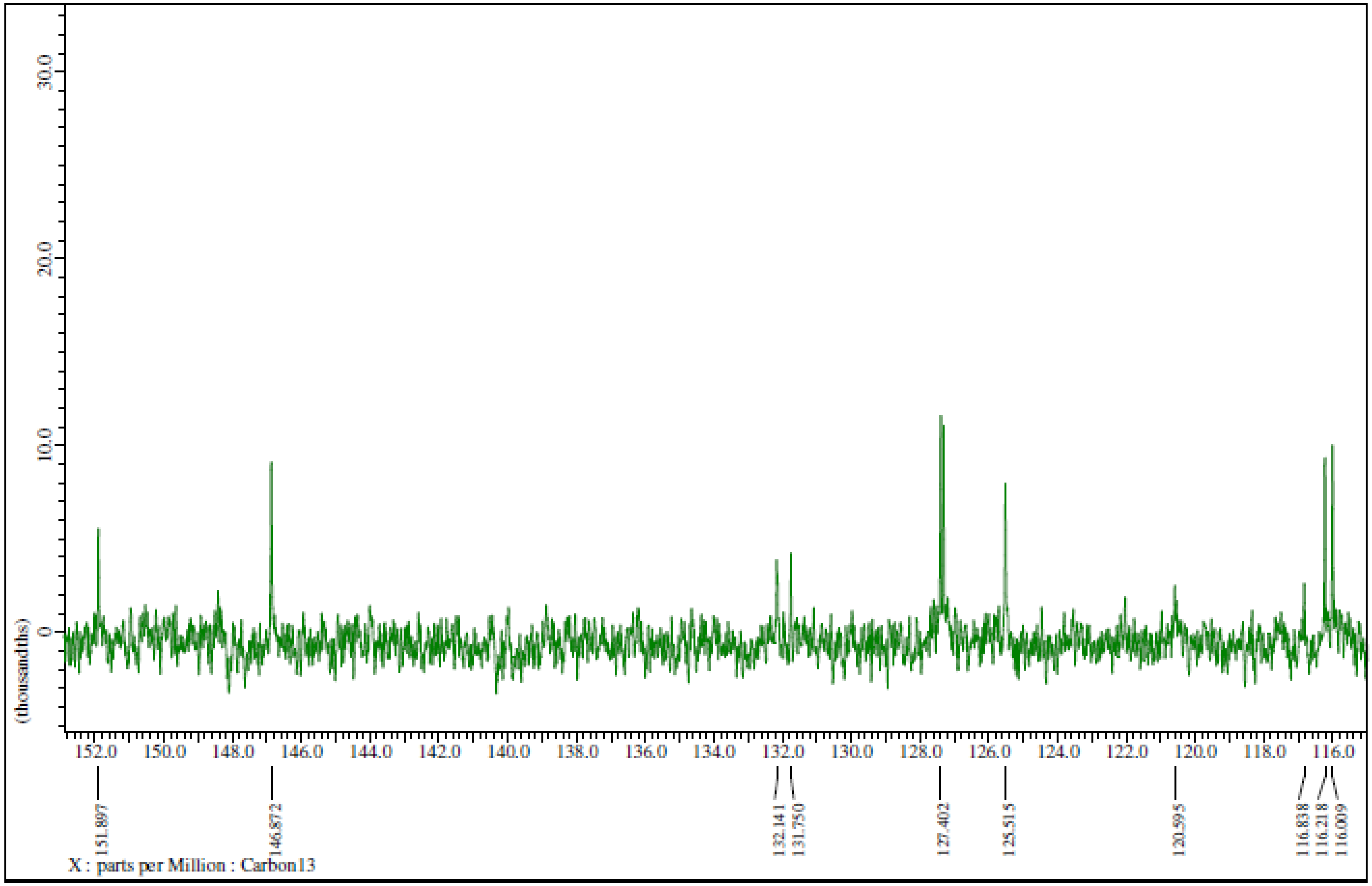

(thousandths)
0
10.0
20.0
30.0
152.0
150.0
148.0
146.0
144.0
142.0
140.0
138.0
136.0
134.0
132.0
130.0
128.0
126.0
124.0
122.0
120.0
118.0
116.0
151.897
146.872
132.141
131.750
127.402
125.515
120.595
116.838
116.218
116.009
X : parts per Million : Carbon13

1-Hydroxy-6-(4-hydroxyphenyl)-pyrazin-2(1*H*)-one **10d**

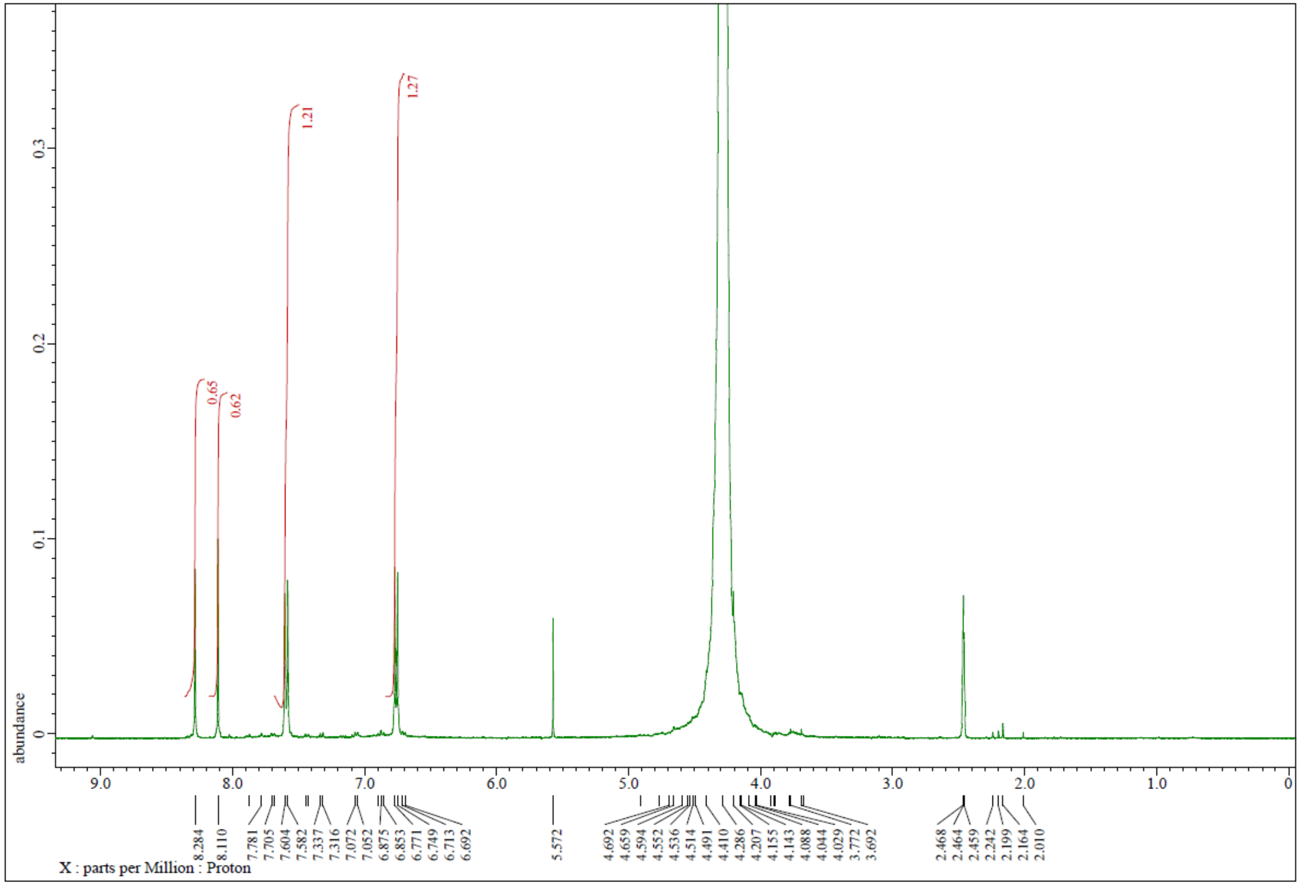

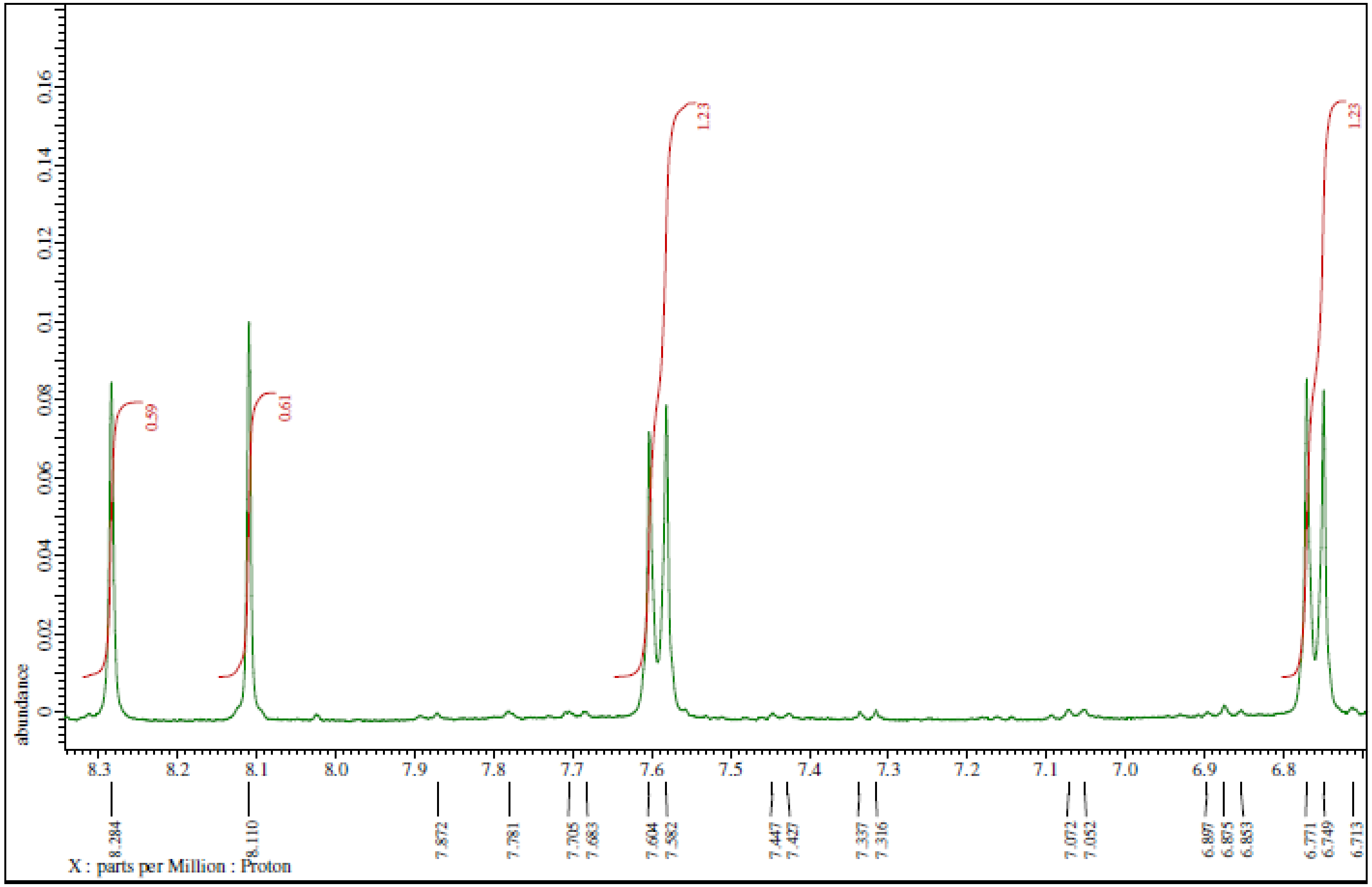
abundance
0
0.02
0.04
0.06
0.08
0.1
0.12
0.14
0.16
0.59
0.61
1.23
1.23
8.3
8.2
8.1
8.0
7.9
7.8
7.7
7.6
7.5
7.4
7.3
7.2
7.1
7.0
6.9
6.8
8.284
8.110
7.872
7.781
7.705
7.683
7.604
7.582
7.447
7.427
7.337
7.316
7.072
7.052
6.897
6.875
6.853
6.771
6.749
6.713
X : parts per Million : Proton

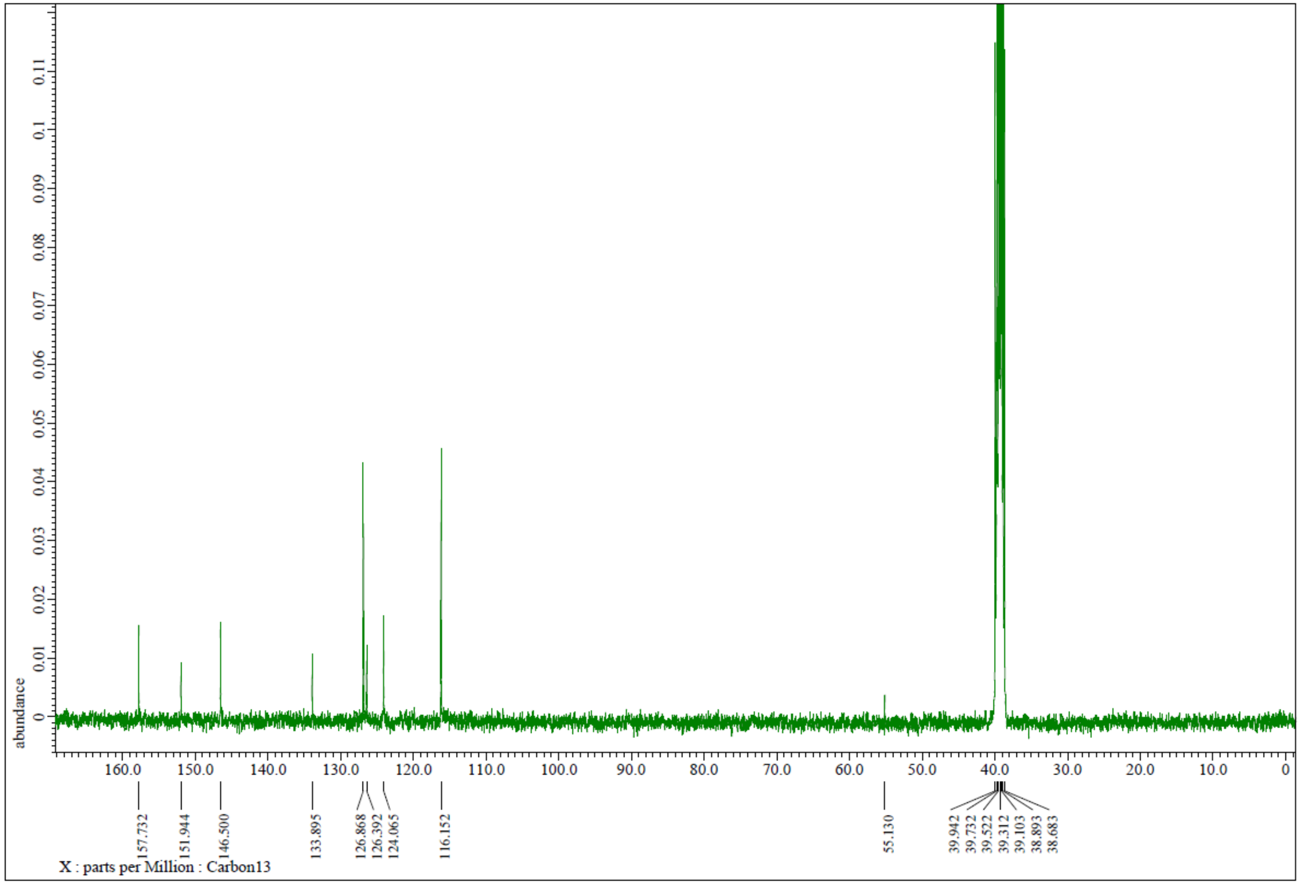
abundance
X : parts per Million : Carbon13
157.732
151.944
146.500
133.895
126.868
126.392
124.065
116.152
55.130
39.942
39.732
39.522
39.312
39.103
38.893
38.683

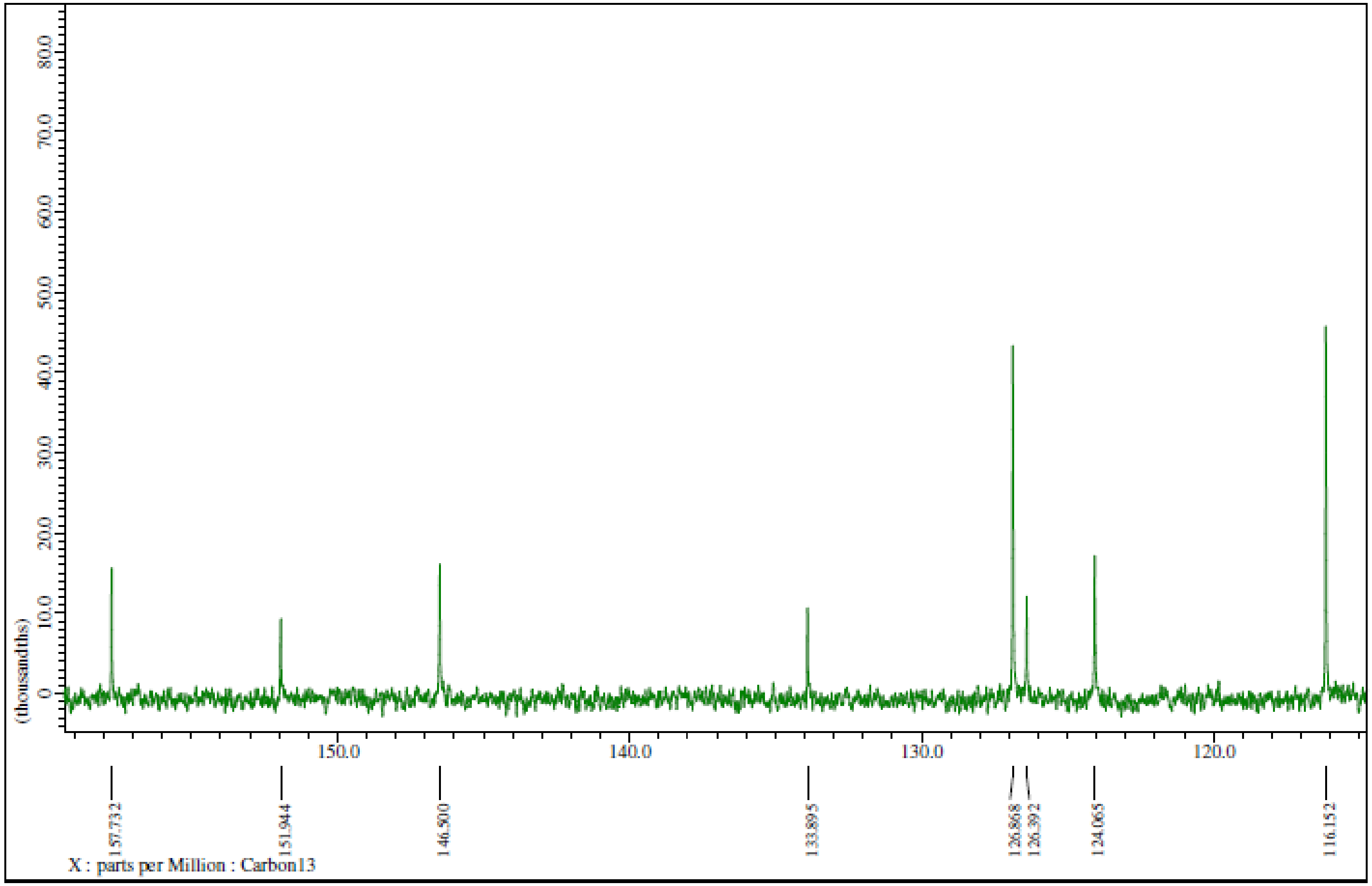
157.732
151.944
146.500
133.895
126.868
126.392
124.065
116.152
150.0
140.0
130.0
120.0
(thousandths)
X : parts per Million : Carbon13

1-Hydroxy-6-methylpyrazin-2(1*H*)-one **11a** and 1-Hydroxy-5-methylpyrazin-2(1*H*)-one **12a**

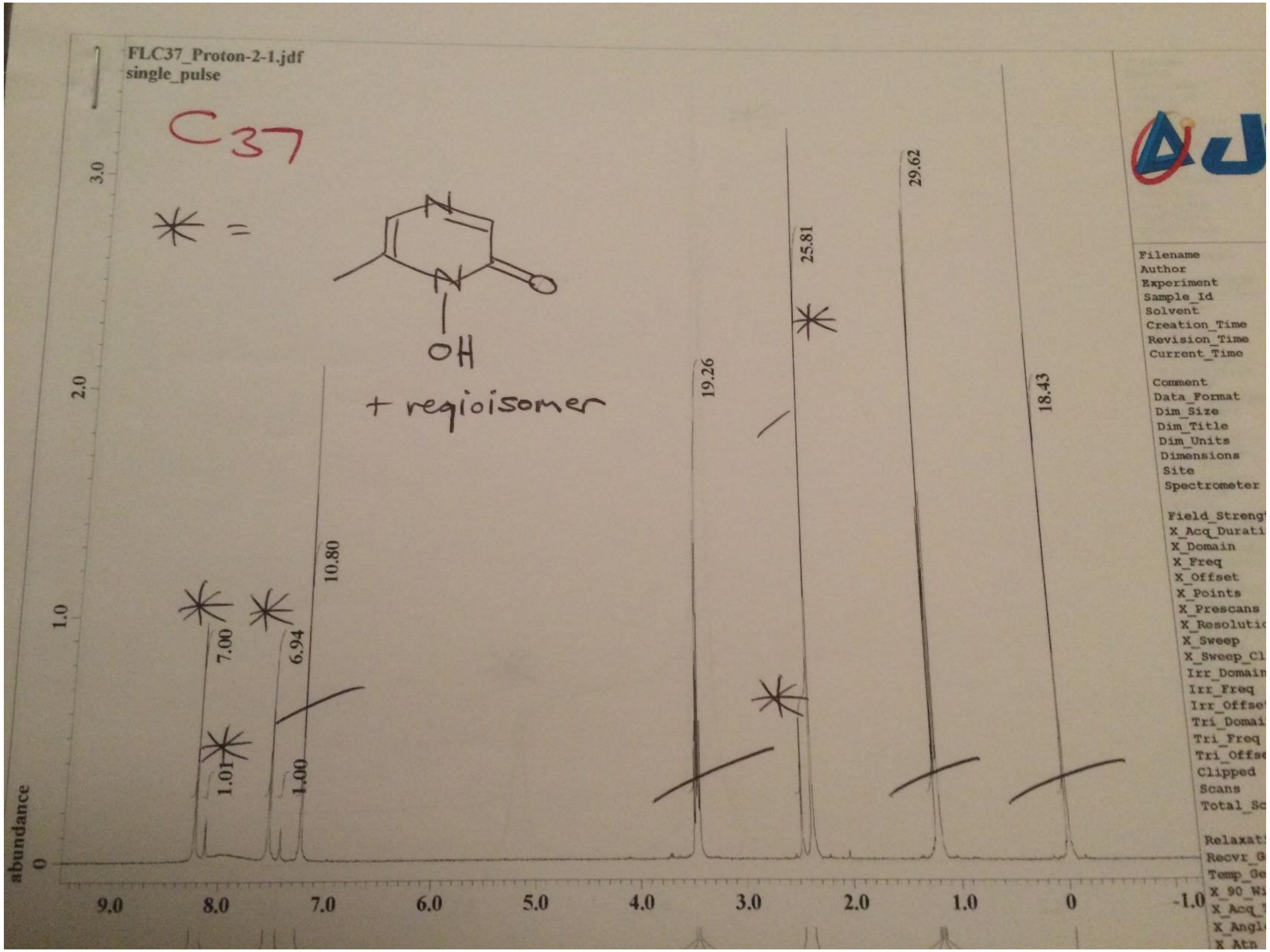

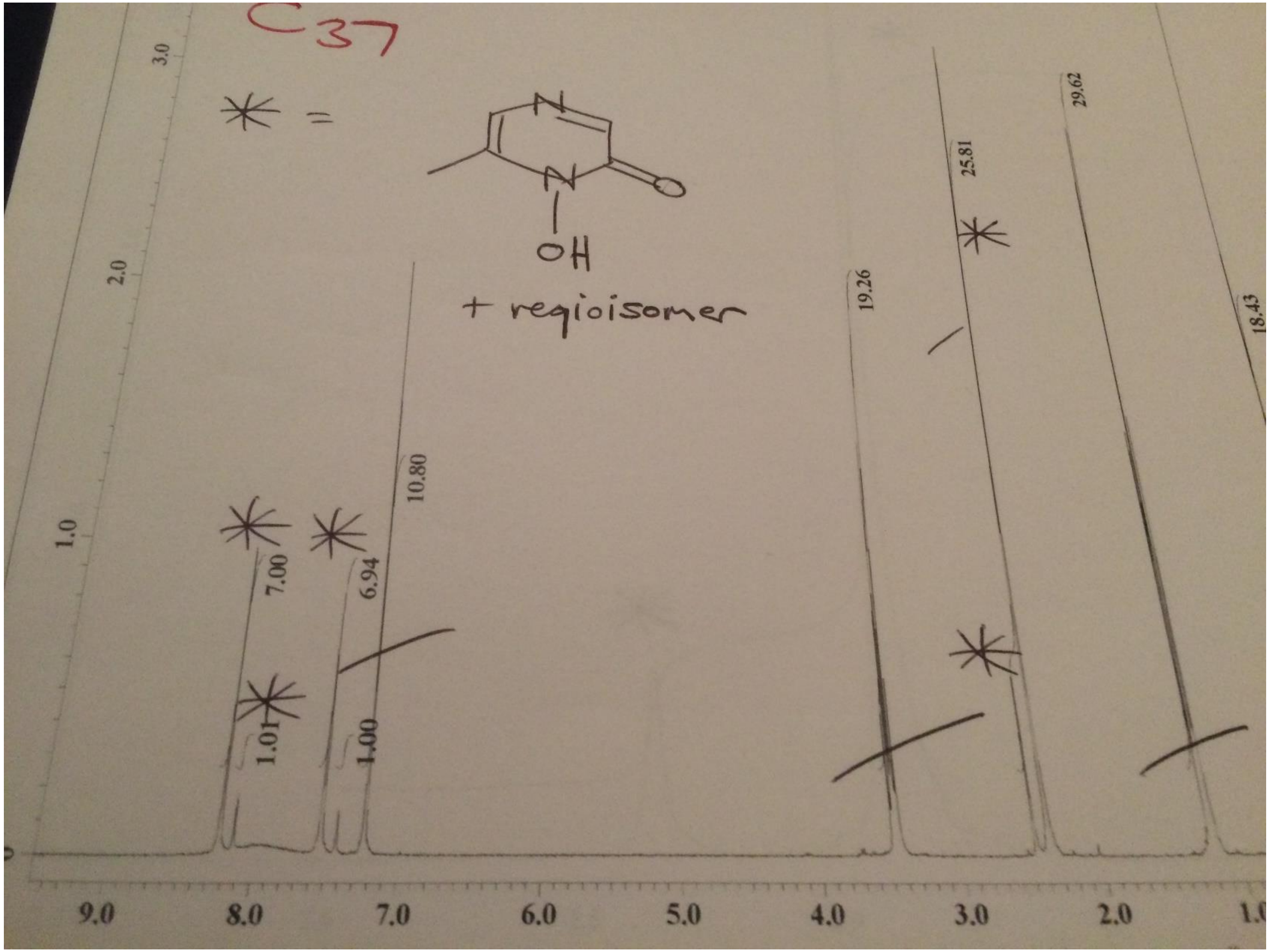
C37
* =
OH
+ regioisomer
7.00
6.94
10.80
1.01
1.00
19.26
25.81
29.62
18.43
9.0
8.0
7.0
6.0
5.0
4.0
3.0
2.0
1.0
2.0
3.0

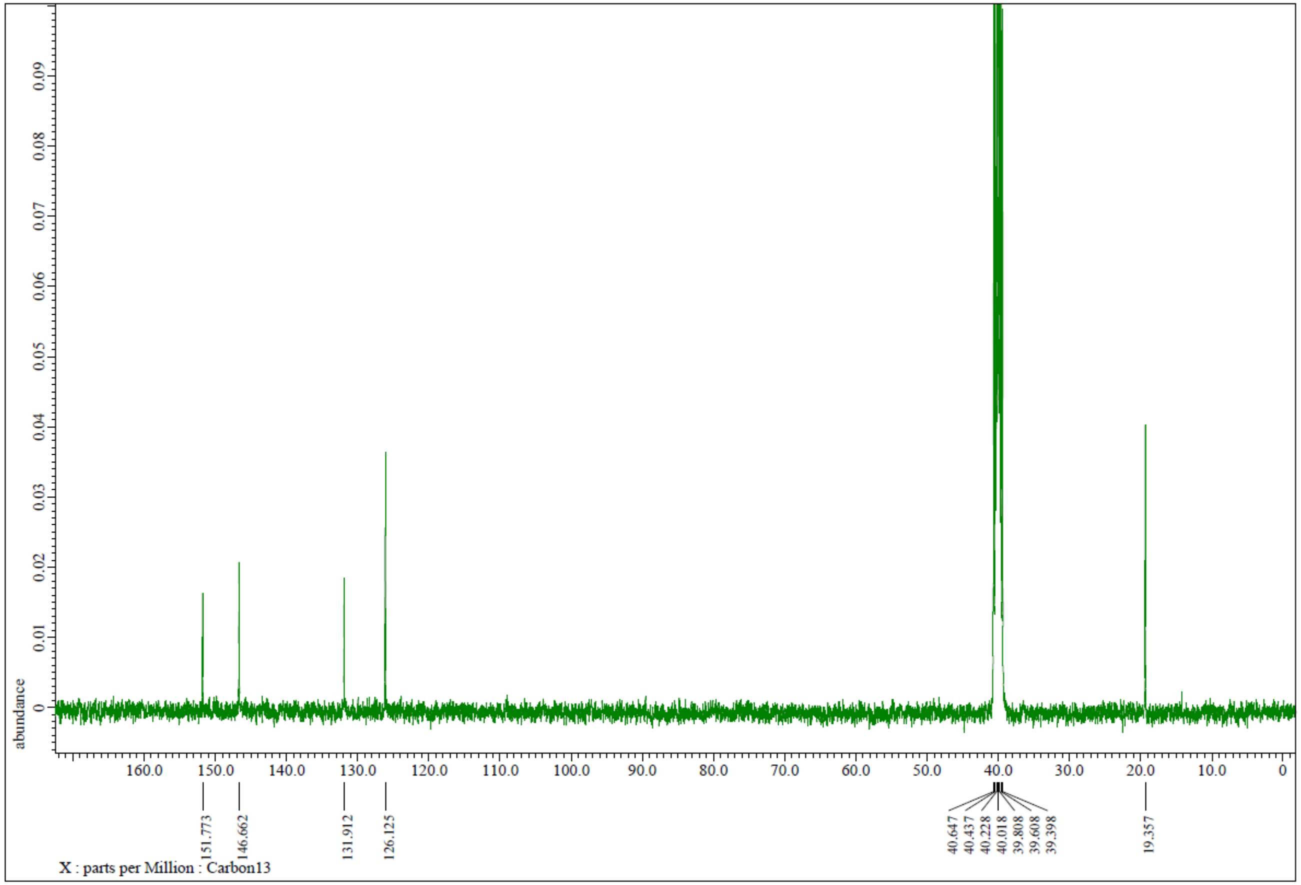
abundance
0
0.01
0.02
0.03
0.04
0.05
0.06
0.07
0.08
0.09
160.0
150.0
140.0
130.0
120.0
110.0
100.0
90.0
80.0
70.0
60.0
50.0
40.0
30.0
20.0
10.0
0
151.773
146.662
131.912
126.125
40.647
40.437
40.228
40.018
39.808
39.608
39.398
19.357
X : parts per Million : Carbon13

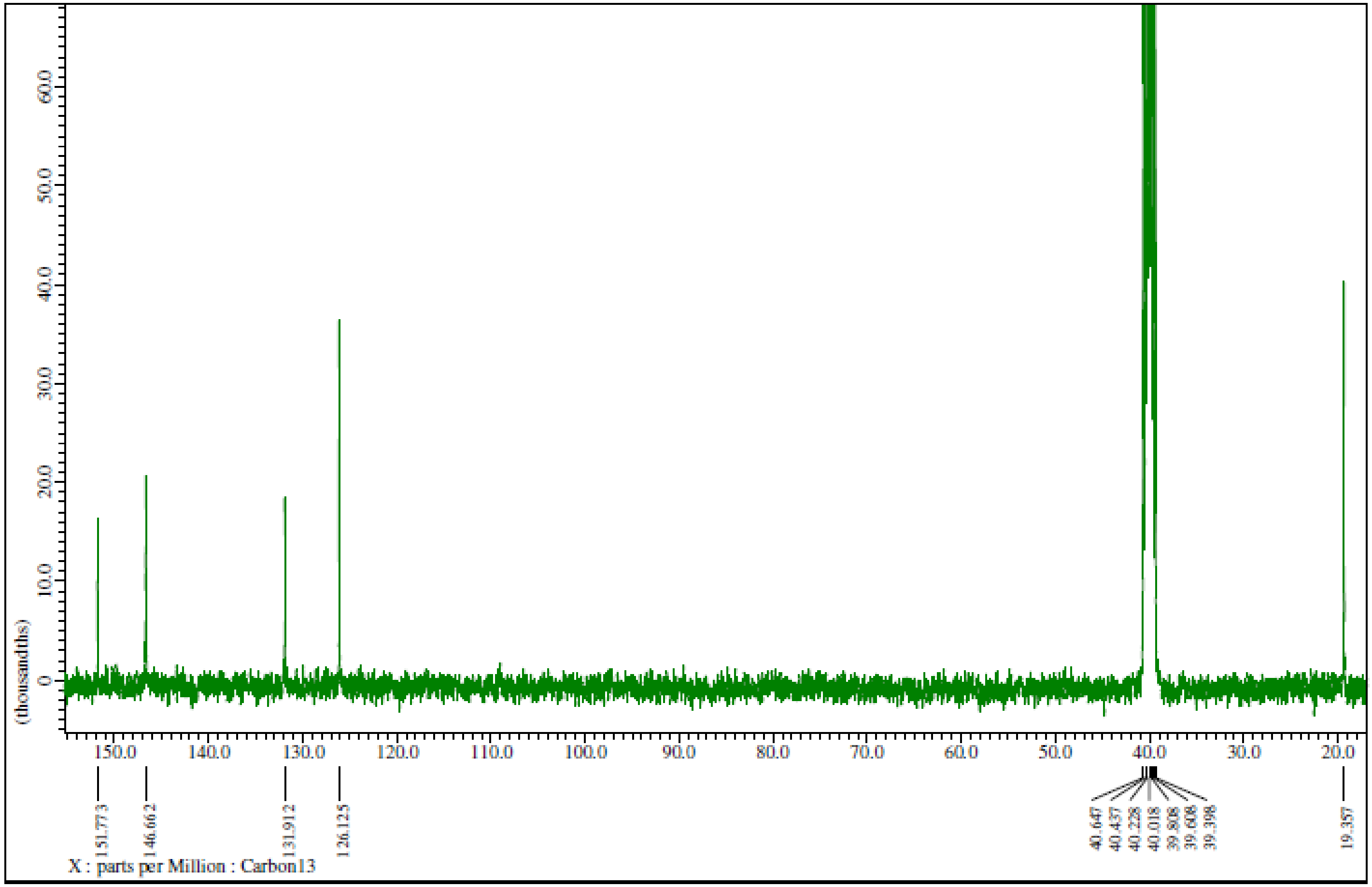

(thousandths)
0
10.0
20.0
30.0
40.0
50.0
60.0
150.0
140.0
130.0
120.0
110.0
100.0
90.0
80.0
70.0
60.0
50.0
40.0
30.0
20.0
151.773
146.662
131.912
126.125
40.647
40.437
40.228
40.018
39.808
39.608
39.398
19.357
X : parts per Million : Carbon13

4. Mass Spectra

1-Hydroxy-3-(4-hydroxybenzyl)-5,6-dimethylpyrazin-2(1*H*)-one **6d**

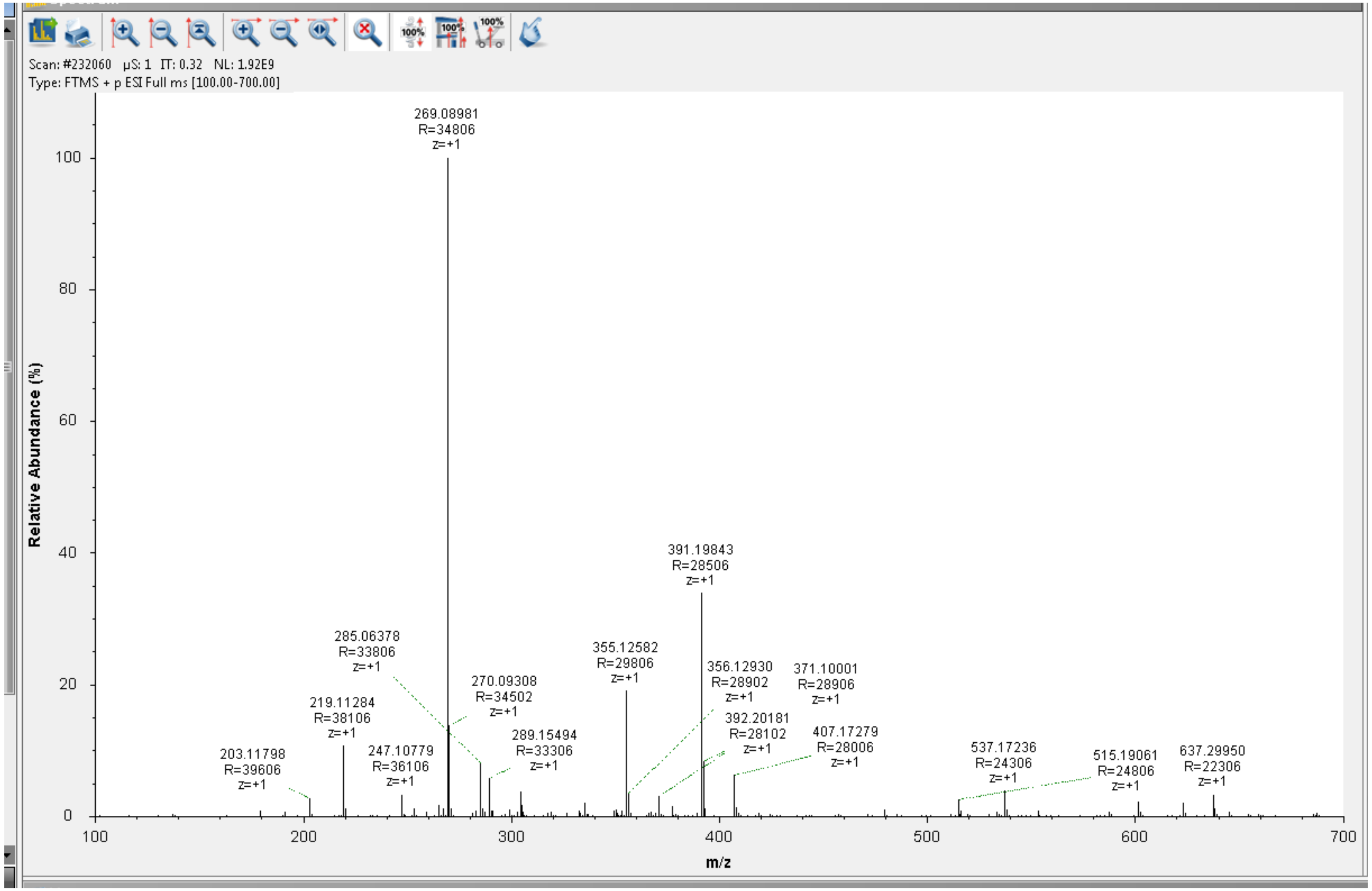

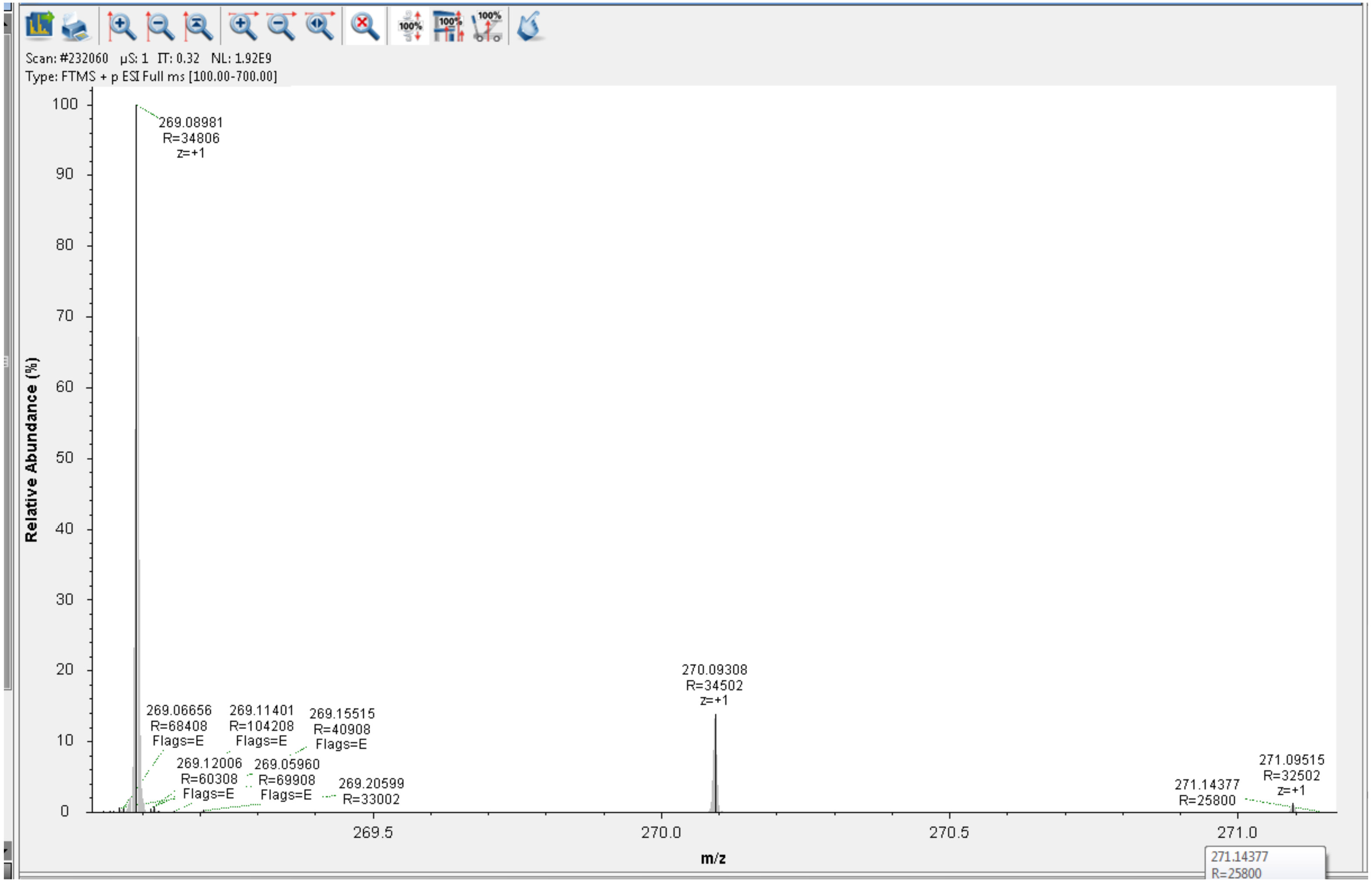
Scan: #232060 µS: 1 IT: 0.32 NL: 1.92E9
Type: FTMS + p ESI Full ms [100.00-700.00]
Relative Abundance (%)
m/z
269.08981
R=34806
z=+1
269.06656
R=68408
Flags=E
269.11401
R=104208
Flags=E
269.15515
R=40908
Flags=E
269.12006
R=60308
Flags=E
269.05960
R=69908
Flags=E
269.20599
R=33002
270.09308
R=34502
z=+1
271.14377
R=25800
271.09515
R=32502
z=+1

1-Hydroxy-6-phenylpyrazin-2(1*H*)-one **10a**

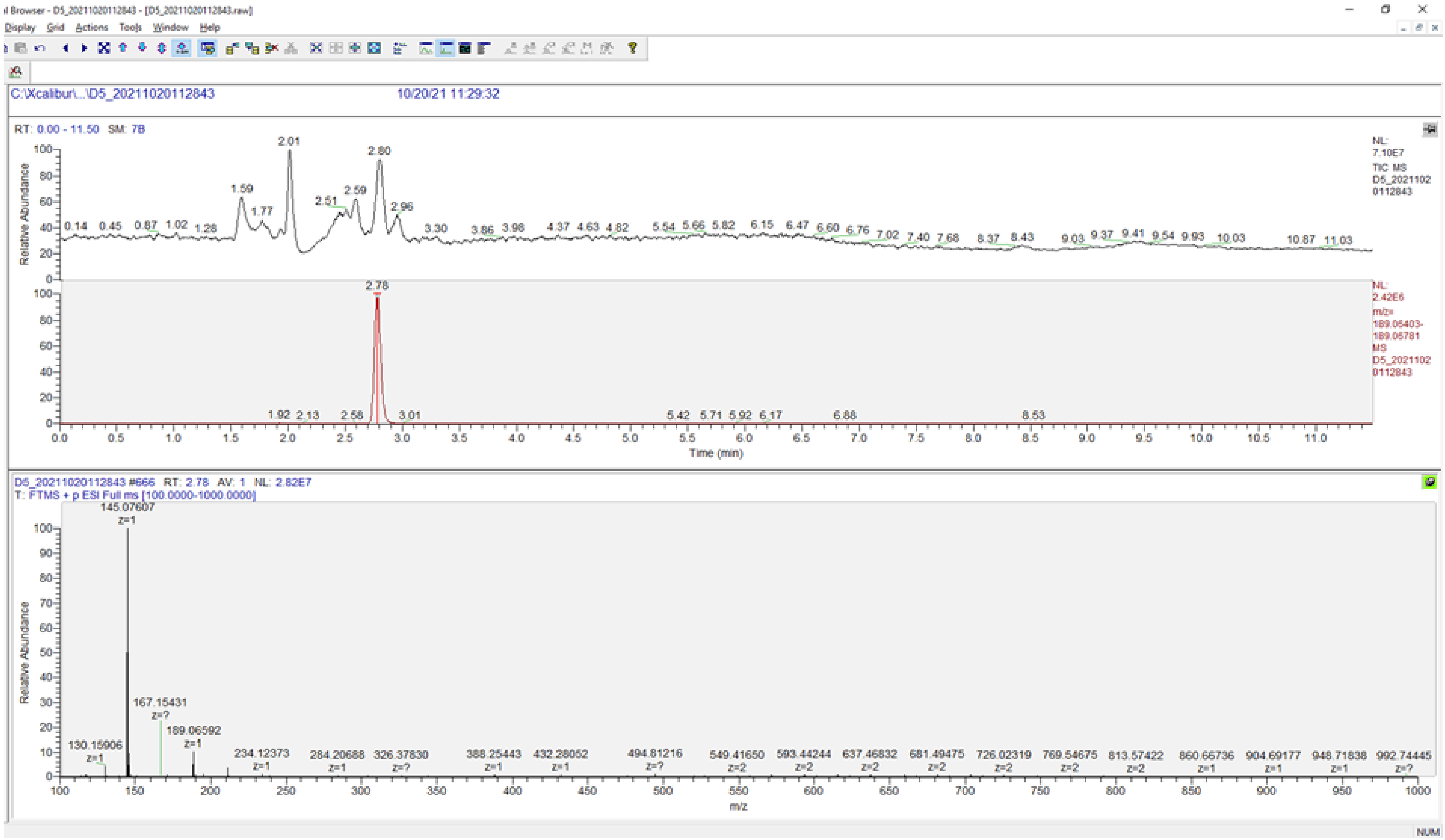

1-Hydroxy-6-(4-methoxyphenyl)-pyrazin-2(1*H*)-one **10b**

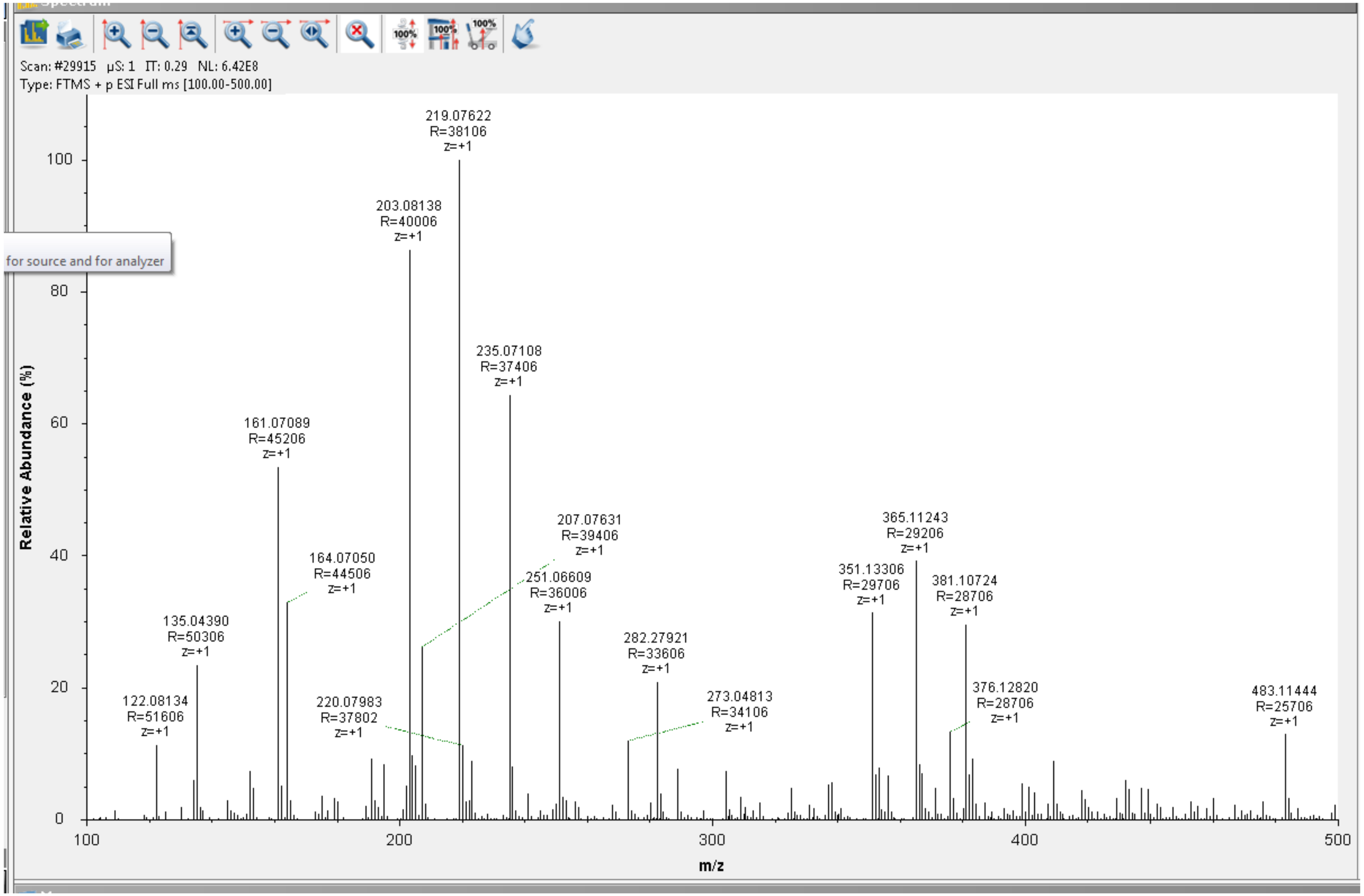

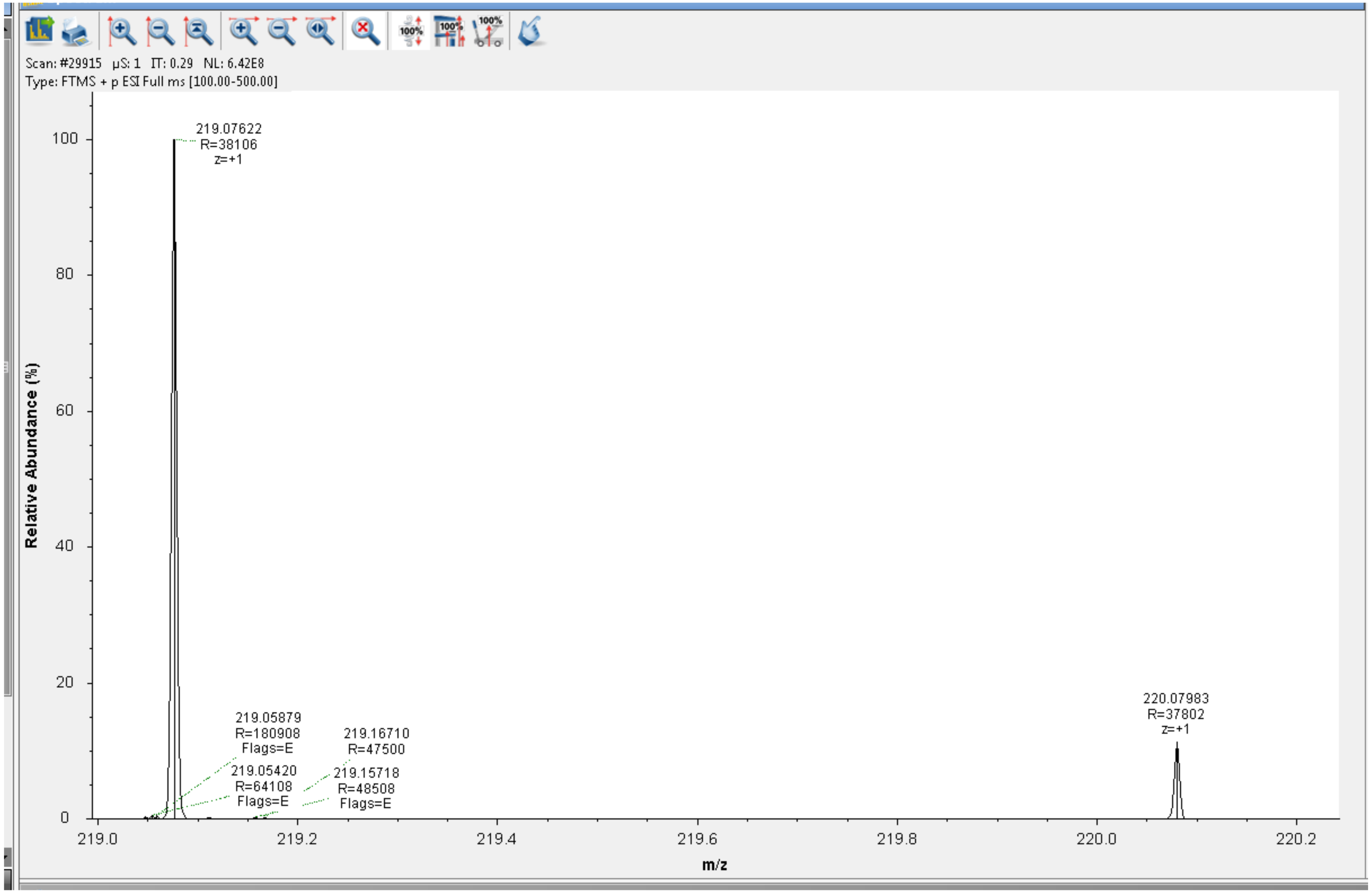

Scan: #29915 µS: 1 IT: 0.29 NL: 6.42E8
Type: FTMS + p ESI Full ms [100.00-500.00]
219.07622
R=38106
z=+1
219.05879
R=180908
Flags=E
219.16710
R=47500
219.05420
R=64108
Flags=E
219.15718
R=48508
Flags=E
220.07983
R=37802
z=+1
Relative Abundance (%)
m/z

1-Hydroxy-6-(4-fluorophenyl)-pyrazin-2(1*H*)-one **10c**

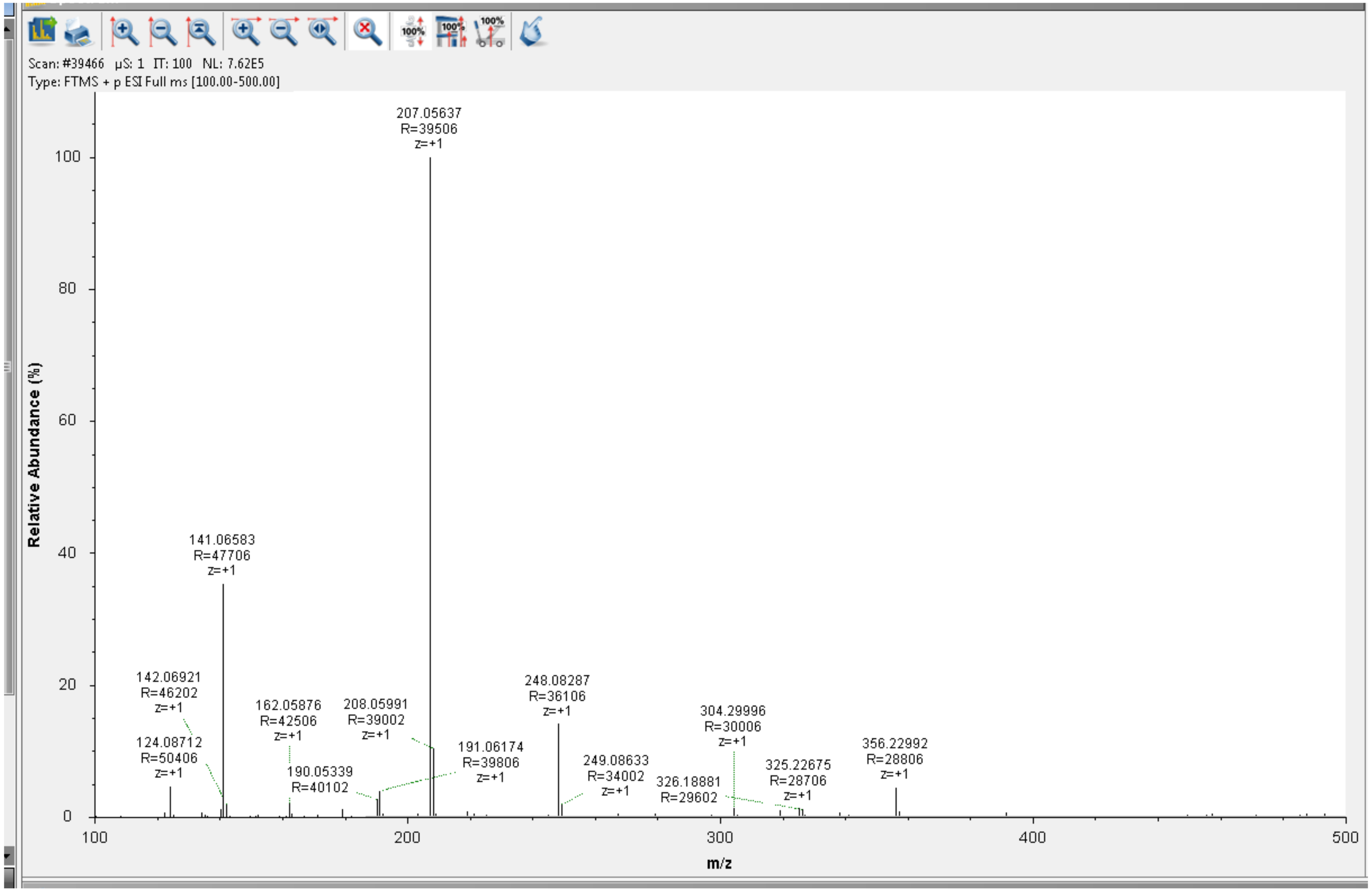

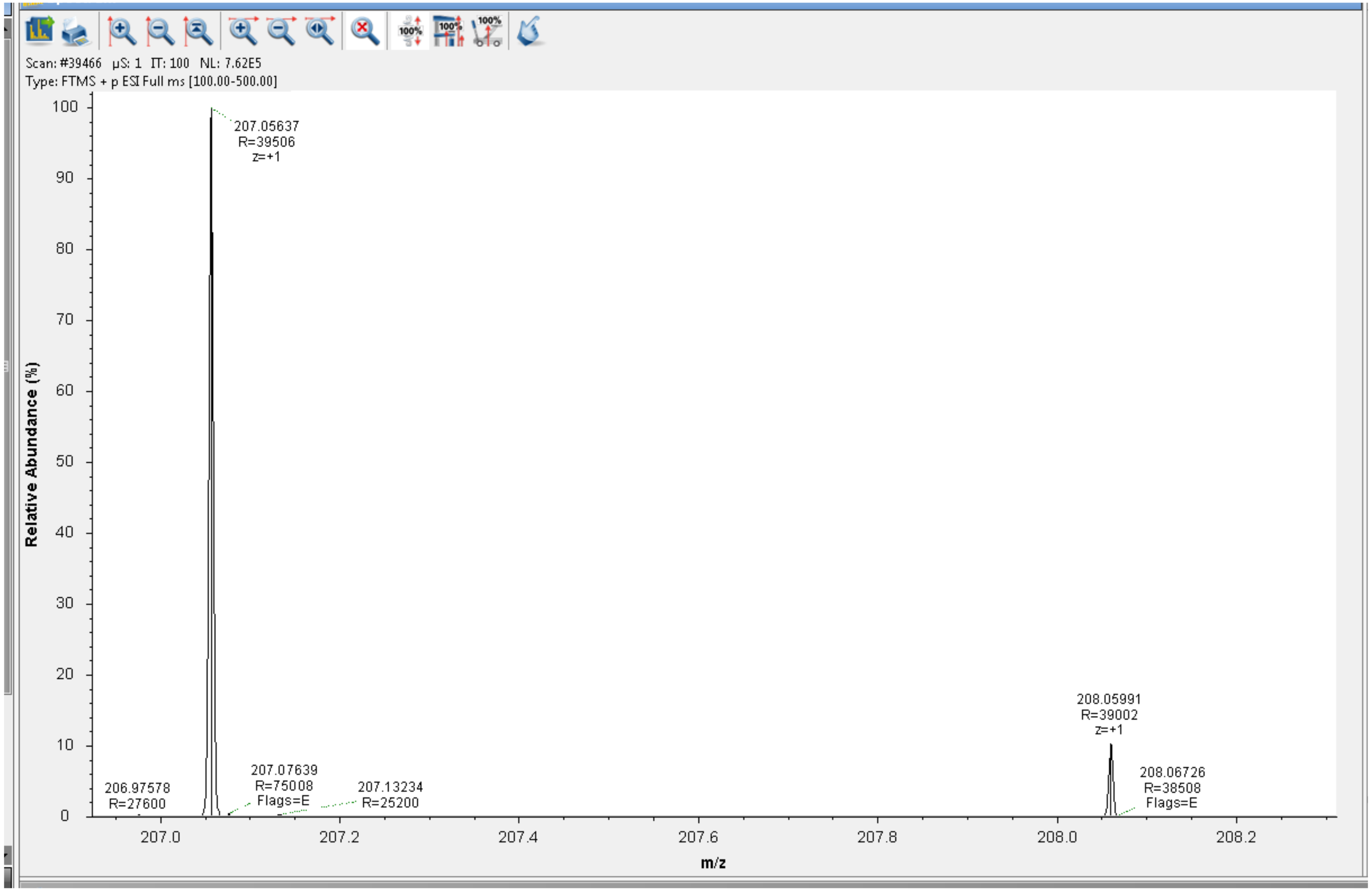

Scan: #39466 µS: 1 IT: 100 NL: 7.62E5
Type: FTMS + p ESI Full ms [100.00-500.00]
Relative Abundance (%)
m/z
207.05637
R=39506
z=+1
206.97578
R=27600
207.07639
R=75008
Flags=E
207.13234
R=25200
208.05991
R=39002
z=+1
208.06726
R=38508
Flags=E

1-Hydroxy-6-(4-hydroxyphenyl)-pyrazin-2(1*H*)-one **10d**

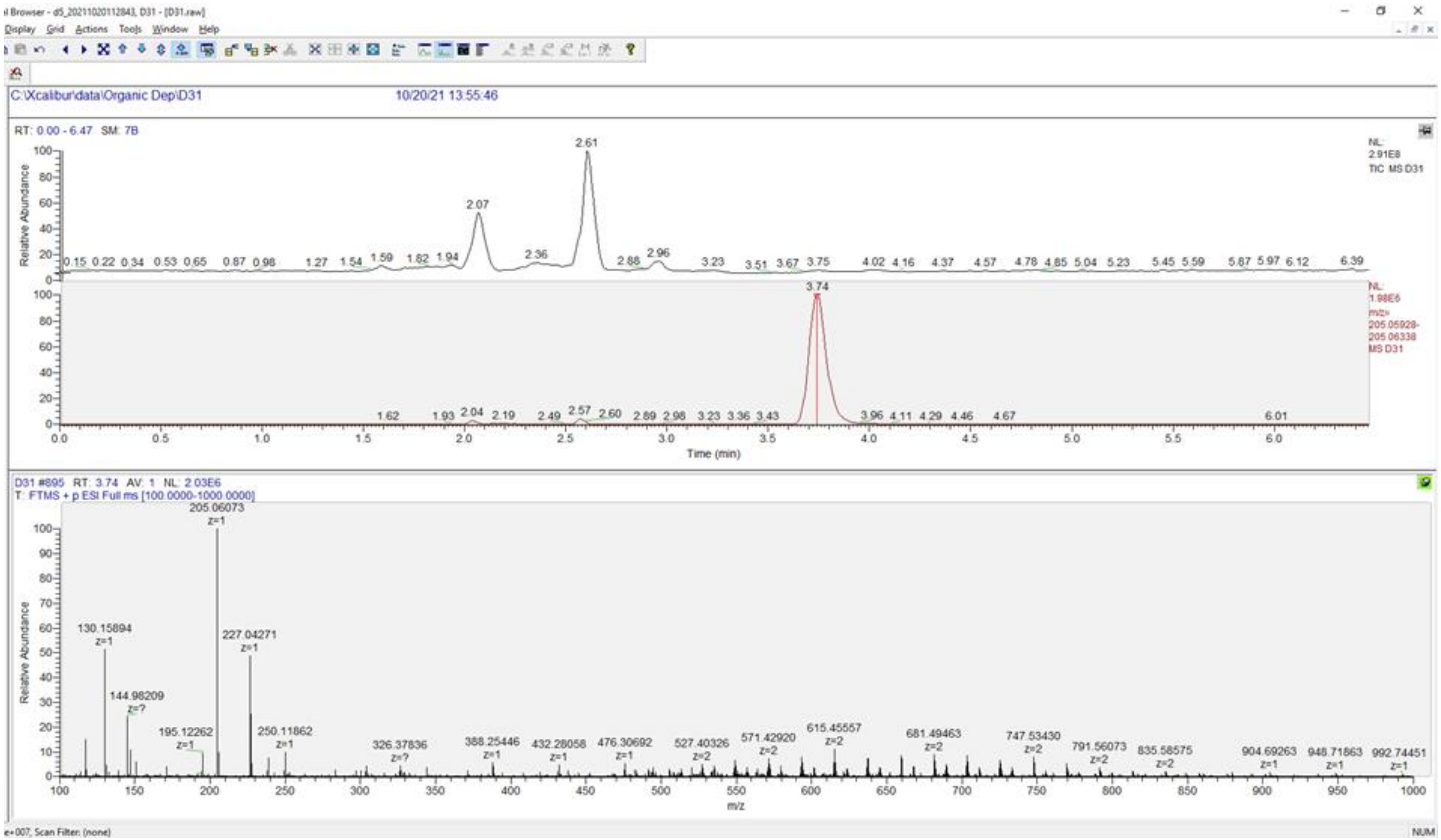

1-Hydroxy-6-methylpyrazin-2(1*H*)-one **11a** and 1-Hydroxy-5-methylpyrazin-2(1*H*)-one **12a**

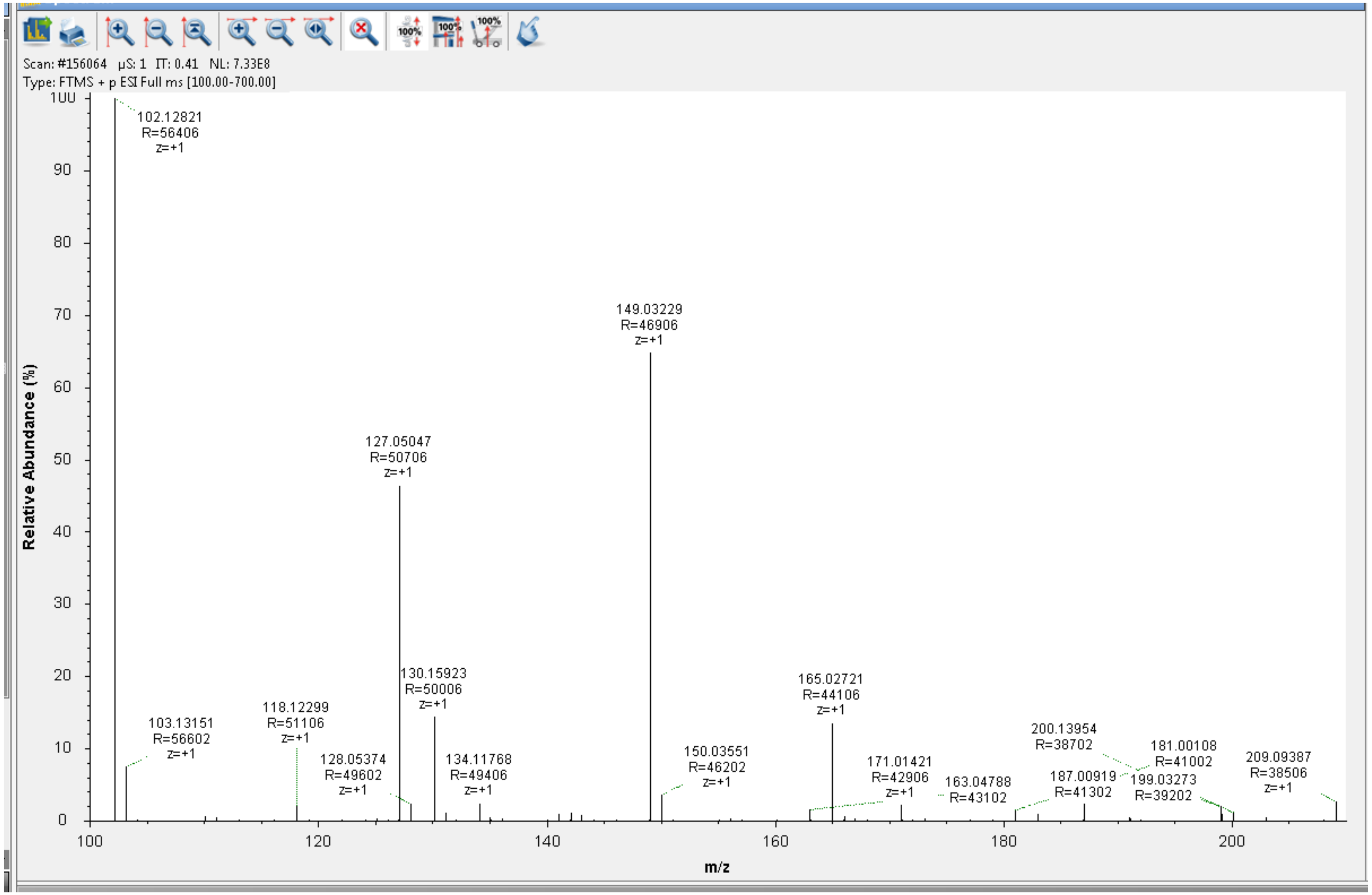

5. Determination of pKa Values of the Ligands and Stability Constants of the Complexes

Protonation studies with ligand 11a

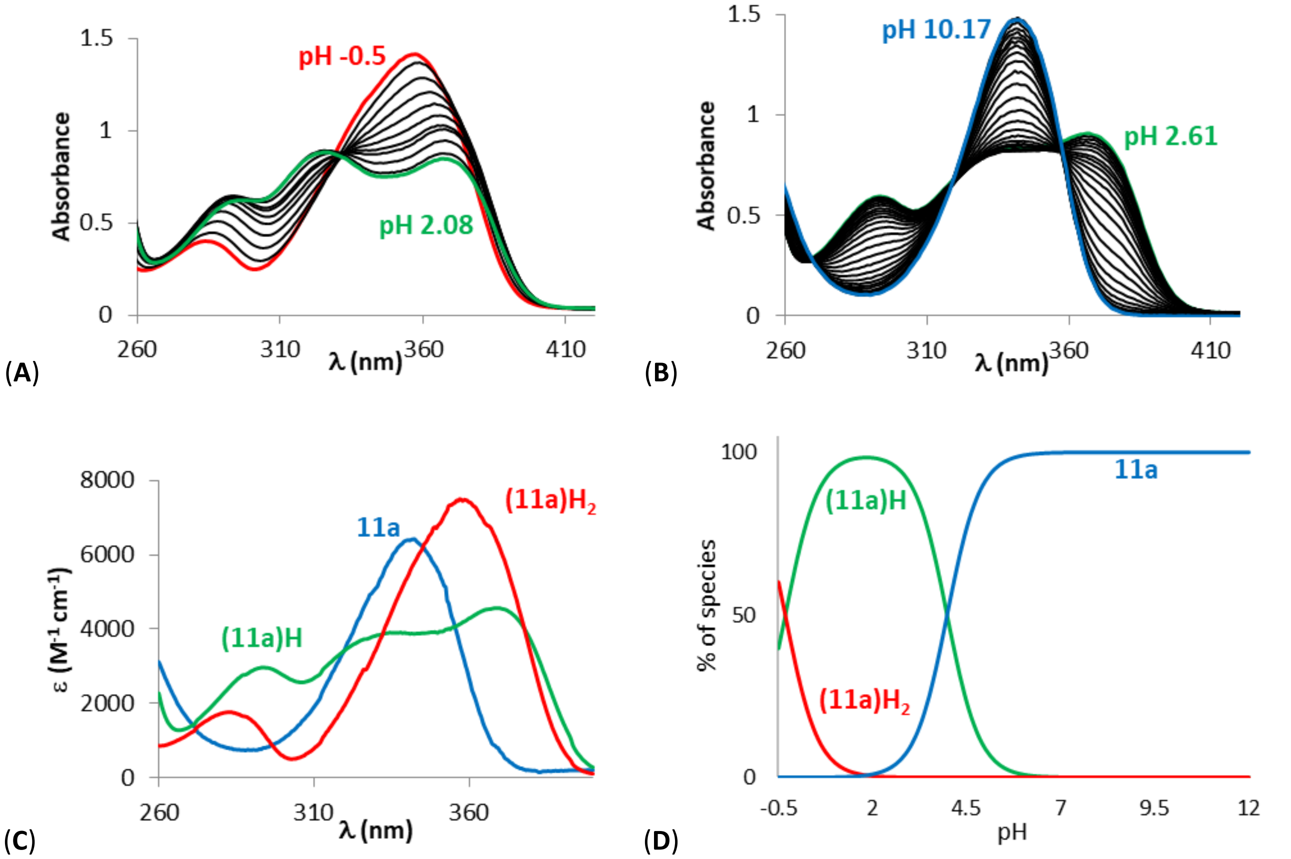

Figure S1. Spectrophotometric titrations vs pH of ligand **11a** between (**A**) −0.5 < pH < 2.08 (batch titration, [**11a**] = 2.57 × 10^{-4} M) and (**B**) 2.61 < pH < 10.17 (direct titration, [**11a**] = 2.55 × 10^{-4} M). (**C**) Electronic spectra and (**D**) distribution curves ([**11a**] = 2.55 × 10^{-4} M) of the protonated species of ligand **11a**. Solvent: H_2O, I = 0.1 M ($NaClO_4$), T= 25.0 °C.

Protonation studies with ligand 10a in water

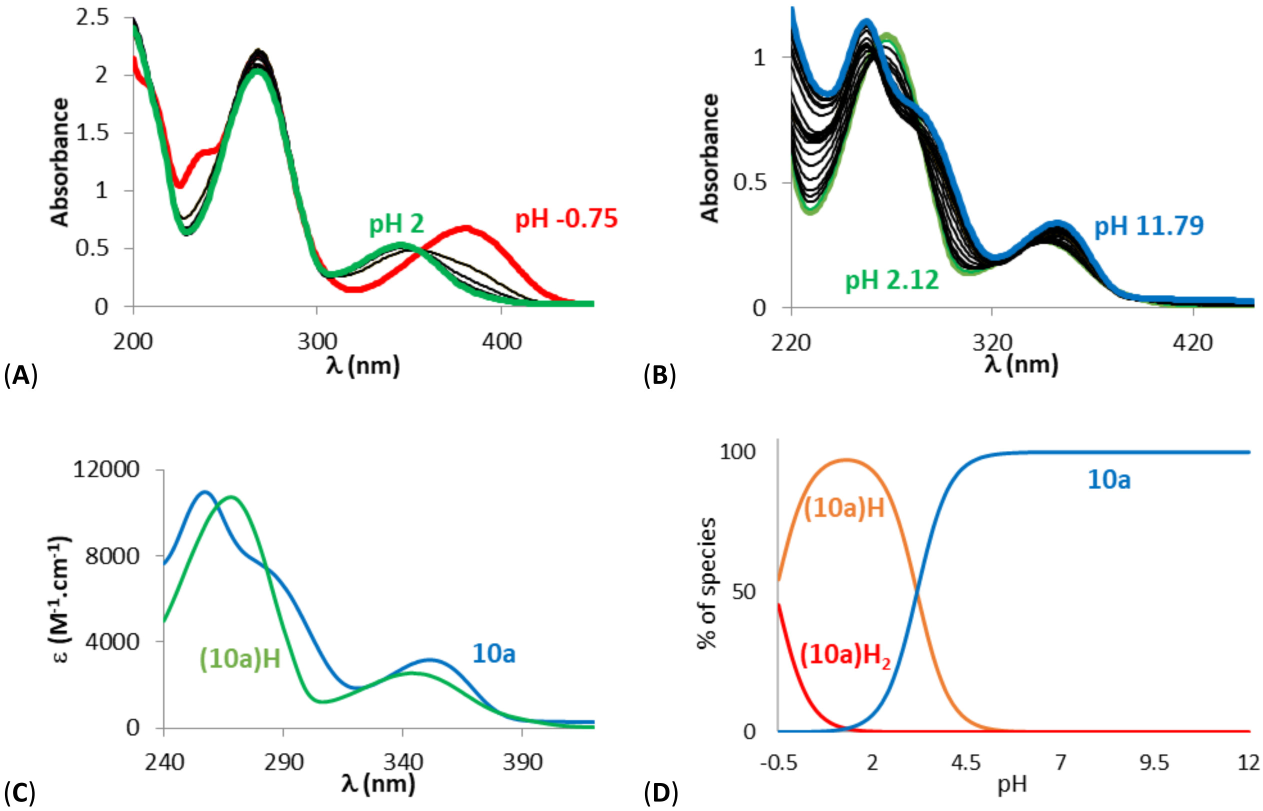

Figure S2. Spectrophotometric titrations vs pH of ligand **10a** between (**A**) −0.75 < pH < 2 (batch titration, [**10a**] = 1.95 × 10^{-4} M) and (**B**) 2.12 < pH < 11.79 (direct titration, [**10a**] = 1.02 × 10^{-4} M). (**C**) Electronic spectra and (**D**) distribution curves ([**10a**] = 1.95 × 10^{-4} M) of the protonated species of ligand **10a**. Solvent: H_2O, I = 0.1 M ($NaClO_4$), T= 25.0 °C.

Protonation studies with ligand 10a in MeOH/H_2O (80/20 w/w)

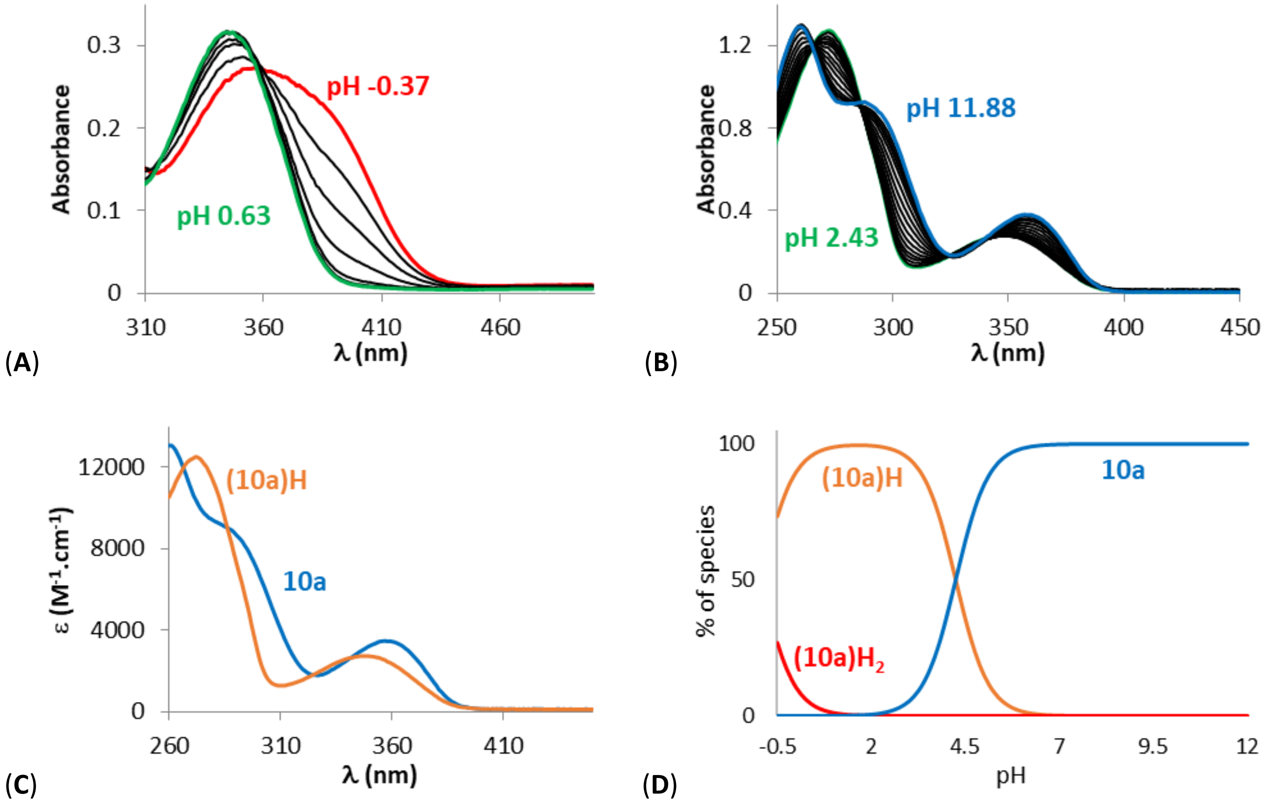

Figure S3. Spectrophotometric titrations vs pH of ligand **10a** between (**A**) −0.37 < pH < 0.63 (batch titration, [**10a**] = 1.02 × 10^{-4} M) and (**B**) 2.43 < pH < 11.88 (direct titration, [**10a**] = 1.01 × 10^{-3} M). (**C**) Electronic spectra and (**D**) distribution curves ([**10a**] = 1.02 × 10^{-4} M) of the protonated species of ligand **10a**. Solvent: MeOH/H_2O (80/20 w/w), I = 0.1 M ($NaClO_4$), T= 25.0 °C.

Protonation studies with ligand 6d

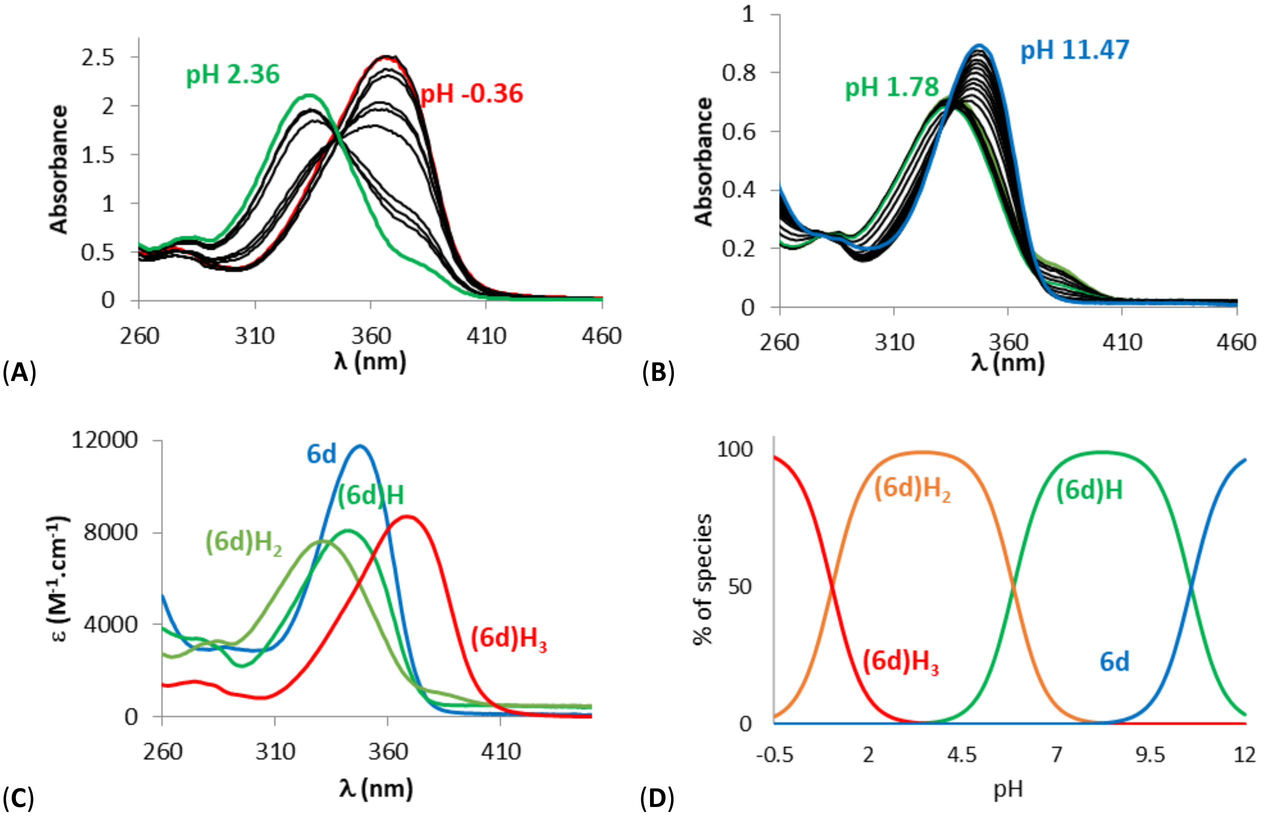

Figure S4. Spectrophotometric titrations vs pH of ligand **6d** between (**A**) −0.36 < pH < 2.36 (batch titration, [**6d**] = 3.0 × 10^{-4} M) and (**B**) 1.78 < pH < 11.47 (direct titration, [**6d**] = 9.98 × 10^{-5} M). (**C**) Electronic spectra and (**D**) distribution curves ([**6d**] = 3.0 × 10^{-4} M) of the protonated species of ligand **6d**. Solvent: MeOH/H_2O (80/20 w/w), I = 0.1 M ($NaClO_4$), T= 25.0 °C.

Protonation studies with ligand 6c

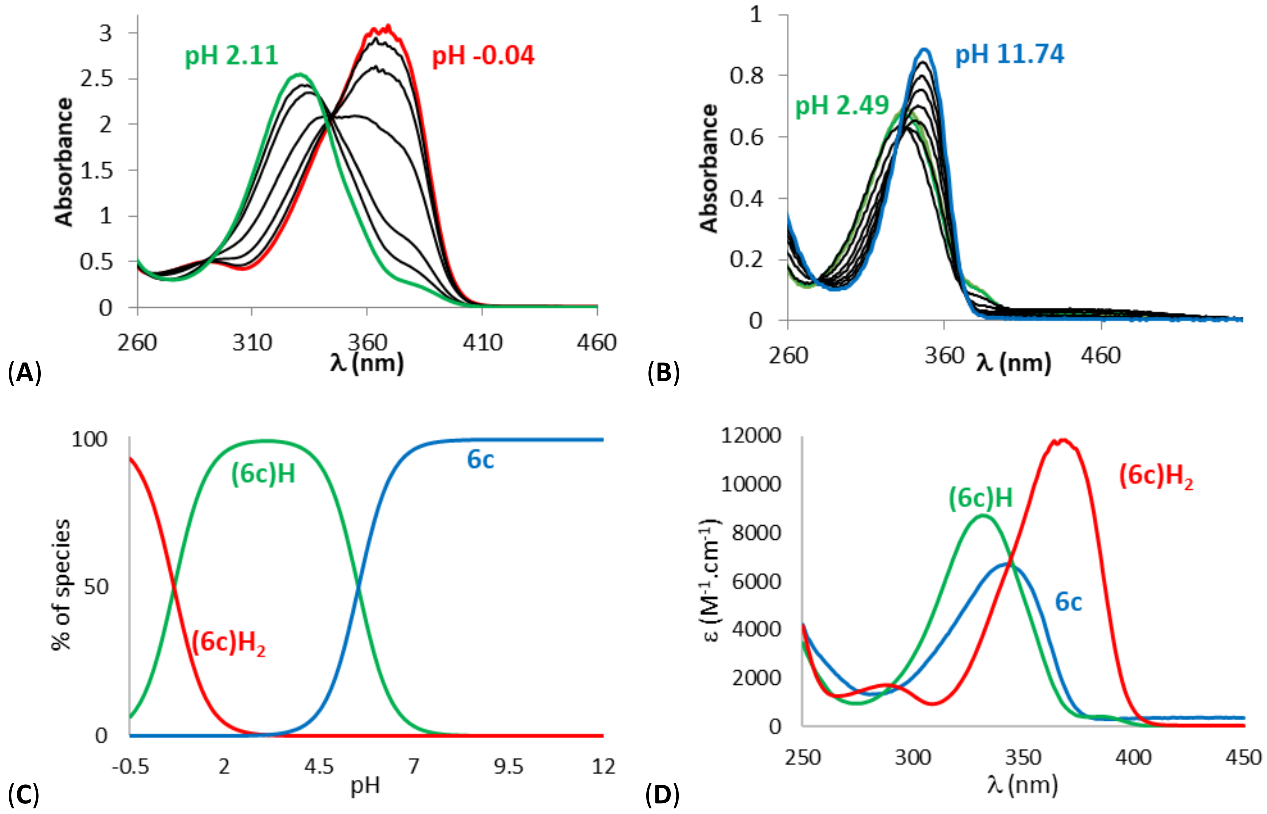

Figure S5. Spectrophotometric titrations vs pH of ligand **6c** between (**A**) −0.04 < pH < 2.11 (batch titration, [**6c**] = 3.0 × 10^{-4} M) and (**B**) 2.49 < pH < 11.74 (direct titration, [**6c**] = 9.98 × 10^{-5} M). (**C**) Electronic spectra and (**D**) distribution curves ([**6c**] = 1.54 × 10^{-4} M) of the protonated species of ligand **6c**. Solvent: MeOH/H_2O (80/20 w/w), I = 0.1 M ($NaClO_4$), T= 25.0 °C.

Fe^{3+} complexation studies with ligand 6a

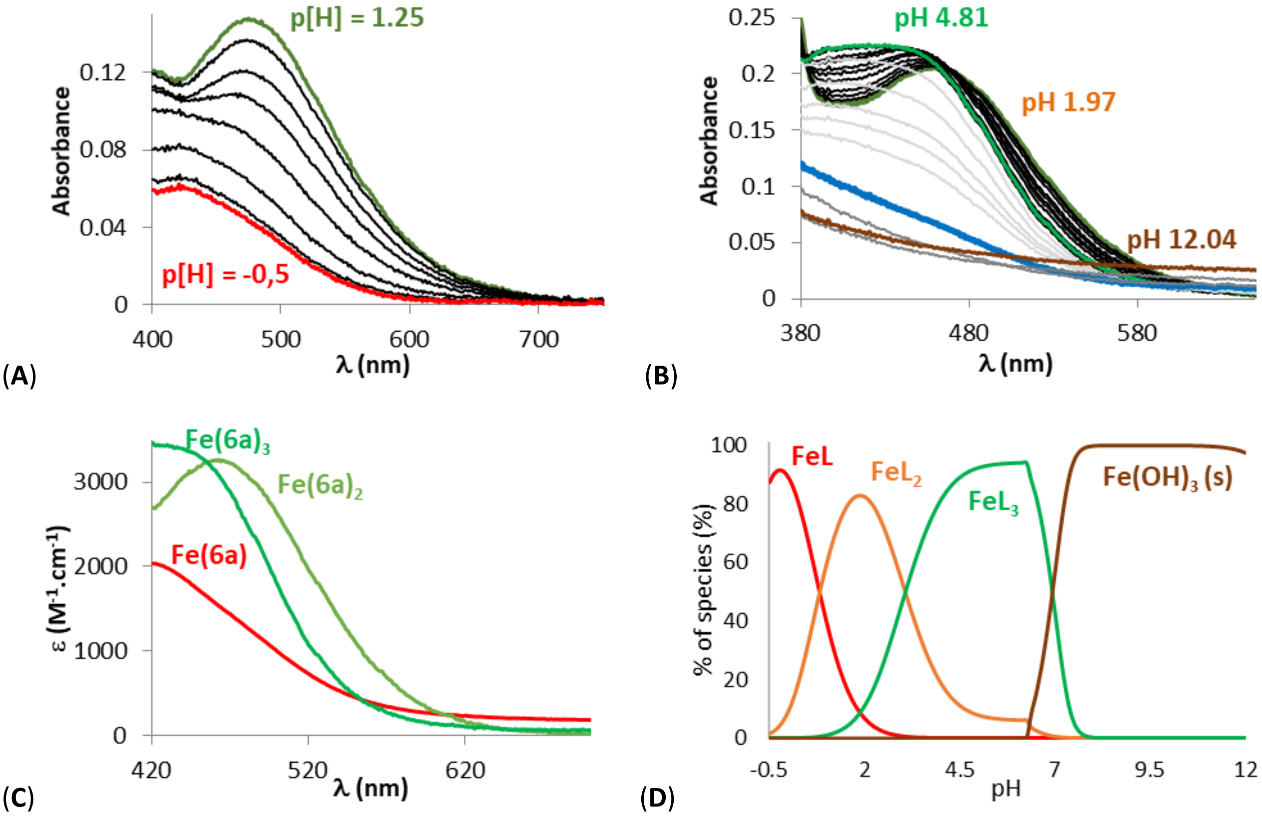

Figure S6. Spectrophotometric titration vs pH of Fe^{3+} complexes of ligand **6a** between (**A**) $-0.5 \leq pH \leq 1.25$ (batch titration, [**6a**] = 3.78×10^{-4} M, [Fe^{3+}] = 1.26×10^{-4} M) and (**B**) $1.97 \leq pH \leq 12.04$ (direct titration, [**6a**] = 2.38×10^{-4} M, [Fe^{3+}] = 7.14×10^{-5} M). (**C**) Electronic spectra and (**D**) distribution curves ([**6a**] = 2.38×10^{-4} M, [Fe^{3+}] = 7.14×10^{-5} M) of the Fe^{3+} complexes of **6a**. Solvent: H_2O, I = 0.1 M ($NaClO_4$), T= 25.0 °C.

Fe^{3+} complexation studies with ligand 10a

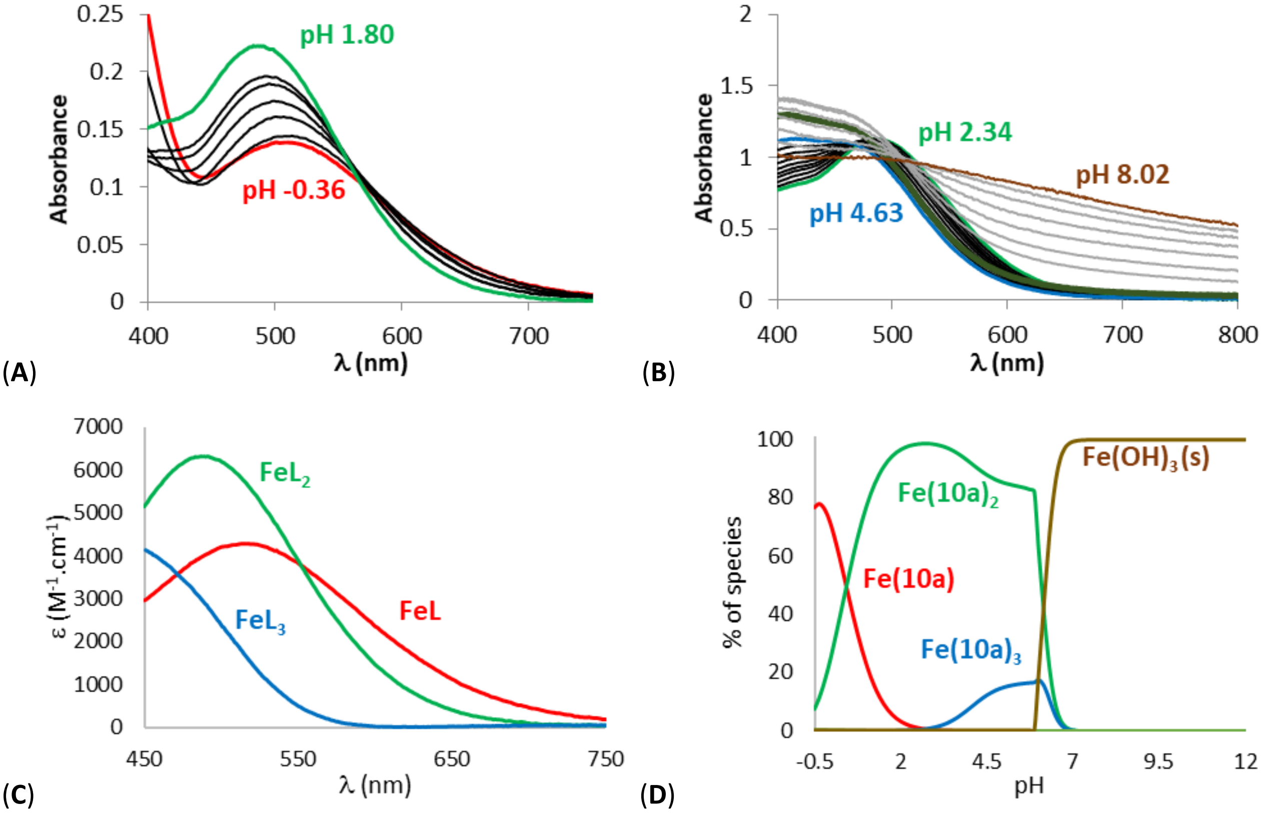

Figure S7. Spectrophotometric titration vs pH of Fe^{3+} complexes of ligand **10a** between (**A**) $-0.36 \leq pH \leq 1.80$ (batch titration, [**10a**] = 1.02×10^{-4} M, [Fe^{3+}] = 3.20×10^{-5} M) and (**B**) $2.34 \leq pH \leq 8.03$ (direct titration, [**10a**] = 1.02×10^{-3} M, [Fe^{3+}] = 3.12×10^{-4} M). (**C**) Electronic spectra and (**D**) distribution curves ([**10a**] = 1.02×10^{-4} M, [Fe^{3+}] = 3.20×10^{-5} M) of the Fe^{3+} complexes of **10a**. Solvent: MeOH/H_2O (80/20 w/w), I = 0.1 M ($NaClO_4$), T= 25.0 °C.

Fe^{3+} complexation studies with ligand 6d

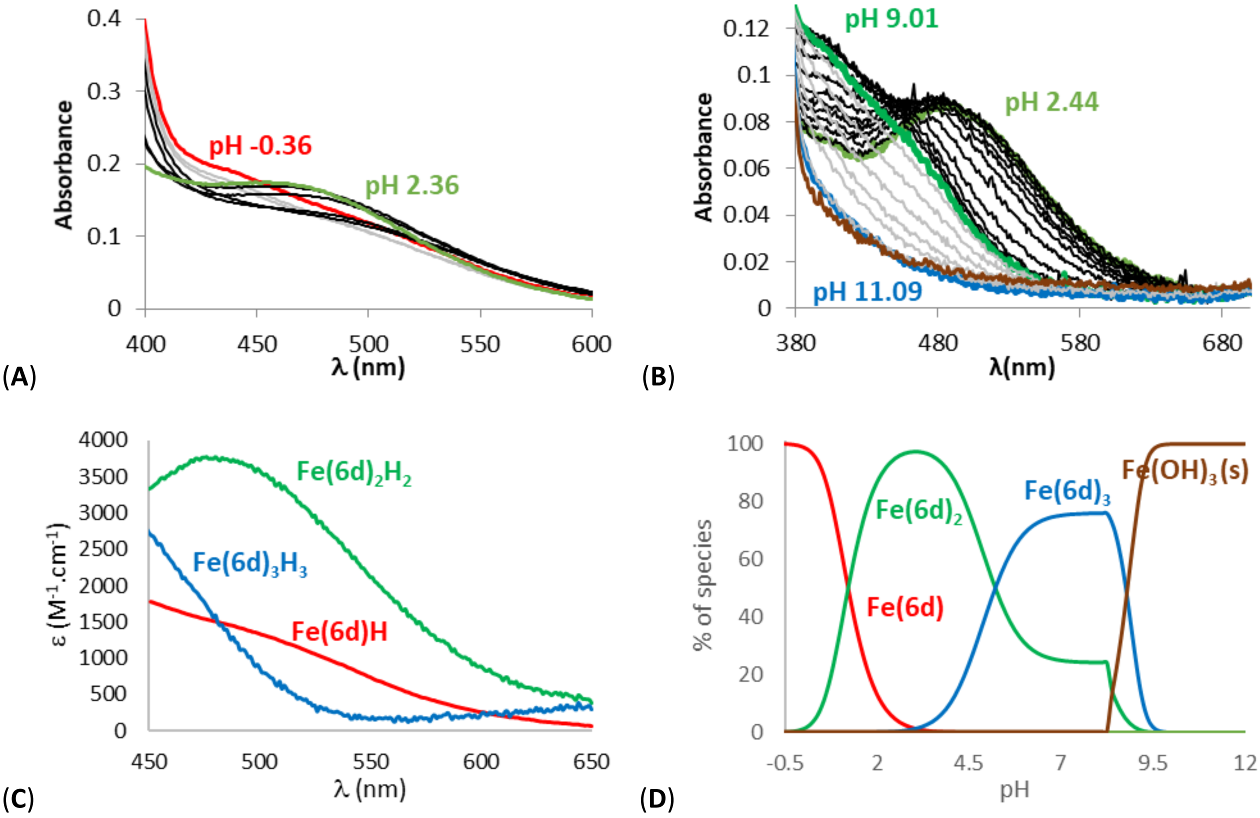

Figure S8. Spectrophotometric titration vs pH of Fe^{3+} complexes of ligand **6d** between (**A**) $-0.36 \leq pH \leq 2.36$ (batch titration, [**6d**] = 3.0×10^{-4} M, [Fe^{3+}] = 8.0×10^{-5} M) and (**B**) $1.97 \leq pH \leq 12.04$ (direct titration, [**6d**] = 1.04×10^{-4} M, [Fe^{3+}] = 3.23×10^{-5} M). (**C**) Electronic spectra and (**D**) distribution curves ([**6d**] = 1.04×10^{-4} M, [Fe^{3+}] = 3.23×10^{-5} M) of the Fe^{3+} complexes of **6d**. Solvent: MeOH/H_2O (80/20 w/w), I = 0.1 M ($NaClO_4$), T= 25.0 °C.

Fe^{3+} complexation studies with ligand 6c

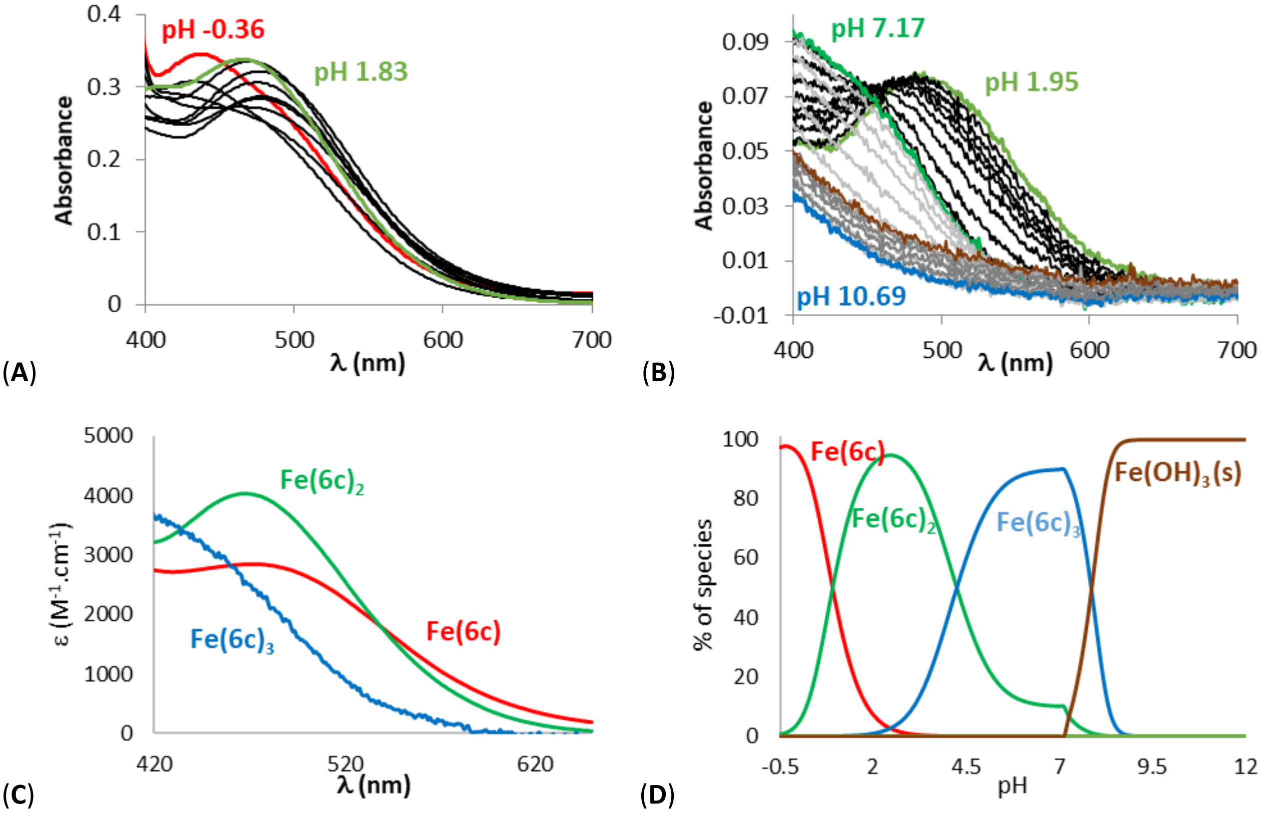

Figure S9. Spectrophotometric titration vs pH of Fe^{3+} complexes of ligand **6c** between (**A**) −0.36 ≤ pH ≤ 1.83 (batch titration, [**6c**] = 3.0 × 10^{-4} M, [Fe^{3+}] = 9.38 × 10^{-5} M) and (**B**) 1.97 ≤ pH ≤ 12.04 (direct titration, [**6c**] = 1.02 × 10^{-4} M, [Fe^{3+}] = 3.12 × 10^{-5} M). (**C**) Electronic spectra and (**D**) distribution curves ([**6c**] = 1.02 × 10^{-4} M, [Fe^{3+}] = 3.12 × 10^{-5} M) of the Fe^{3+} complexes of **6c**. Solvent: MeOH/H_2O (80/20 w/w), I = 0.1 M ($NaClO_4$), T= 25.0 °C.

6. BBB Penetration Scores

Table S1. Predicted BBB score of compound **6a**.

**6a**		
**Property**	**Value**	**T_0**
Number of Aromatic Rings (Aro_R)	1	0.82
Number of Heavy Atoms (HA)	10	0.65
Molecular Weight (MW)	140.14	
Number of Hydrogen Bond Acceptor (HBA)	3	
Number of Hydrogen Bond Donor (HBD)	1	
MWHBN [MWHBN = (MW^(-0.5)*HBN), where HBN=HBA+HBD]	0.34	0.71
Topological Polar Surface Area(TPSA)	55.12	0.62
pKa	4.58	0.23
**BBB SCORE**		3.88

Table S2. Predicted BBB score of compound **6c**.

**6c**		
**Property**	**Value**	**T_0**
Number of Aromatic Rings (Aro_R)	2	1.00
Number of Heavy Atoms (HA)	17	0.98
Molecular Weight (MW)	230.26	
Number of Hydrogen Bond Acceptor (HBA)	3	
Number of Hydrogen Bond Donor (HBD)	1	
MWHBN [MWHBN = (MW^(-0.5)*HBN), where HBN=HBA+HBD]	0.26	0.93
Topological Polar Surface Area(TPSA)	65.42	0.54
pKa	5.53	0.47
**BBB SCORE**		4.70

Table S3. Predicted BBB score of compound **6d**.

**6d**		
**Property**	**Value**	**T_0**
Number of Aromatic Rings (Aro_R)	2	1.00
Number of Heavy Atoms (HA)	18	0.99
Molecular Weight (MW)	246.26	
Number of Hydrogen Bond Acceptor (HBA)	4	
Number of Hydrogen Bond Donor (HBD)	2	
MWHBN [MWHBN = (MW^(-0.5)*HBN), where HBN=HBA+HBD]	0.38	0.55
Topological Polar Surface Area(TPSA)	75.35	0.47
pKa	5.96	0.58
**BBB SCORE**		4.05

Table S4. Predicted BBB score of compound **10a**.

**10a**		
**Property**	**Value**	**T_0**
Number of Aromatic Rings (Aro_R)	2	1.00
Number of Heavy Atoms (HA)	14	0.89
Molecular Weight (MW)	188.18	
Number of Hydrogen Bond Acceptor (HBA)	3	
Number of Hydrogen Bond Donor (HBD)	1	
MWHBN [MWHBN = (MW^(-0.5)*HBN), where HBN=HBA+HBD]	0.29	0.87
Topological Polar Surface Area(TPSA)	51.48	0.64
pKa	3.18	0.00
**BBB SCORE**		4.47

Table S5. Predicted BBB score of compound **10d**.

**10d**		
**Property**	**Value**	**T_0**
Number of Aromatic Rings (Aro_R)	2	1.00
Number of Heavy Atoms (HA)	15	0.93
Molecular Weight (MW)	204.18	
Number of Hydrogen Bond Acceptor (HBA)	4	
Number of Hydrogen Bond Donor (HBD)	2	
MWHBN [MWHBN = (MW^(-0.5)*HBN), where HBN=HBA+HBD]	0.42	0.40
Topological Polar Surface Area(TPSA)	53.5	0.63
pKa	4	0.11
**BBB SCORE**		3.83

Table S6. Predicted BBB score of compound **11a**.

**11a**		
**Property**	**Value**	**T_0**
Number of Aromatic Rings (Aro_R)	1	0.82
Number of Heavy Atoms (HA)	9	0.56
Molecular Weight (MW)	126.11	
Number of Hydrogen Bond Acceptor (HBA)	3	
Number of Hydrogen Bond Donor (HBD)	1	
MWHBN [MWHBN = (MW^(-0.5)*HBN), where HBN=HBA+HBD]	0.36	0.65
Topological Polar Surface Area(TPSA)	31.01	0.78
pKa	3.98	0.11
**BBB SCORE**		3.97

Table S7. Predicted BBB score of compound **2**.

**2**		
**Property**	**Value**	**T_0**
Number of Aromatic Rings (Aro_R)	1	0.82
Number of Heavy Atoms (HA)	10	0.65
Molecular Weight (MW)	141.12	
Number of Hydrogen Bond Acceptor (HBA)	3	
Number of Hydrogen Bond Donor (HBD)	2	
MWHBN [MWHBN = (MW^(-0.5)*HBN), where HBN=HBA+HBD]	0.42	0.39
Topological Polar Surface Area(TPSA)	34.37	0.76
pKa	6.02	0.60
**BBB SCORE**		3.87

Table S8. Predicted BBB score of compound **3**.

**3**		
**Property**	**Value**	$\mathbf{T_0}$
Number of Aromatic Rings (Aro_R)	1	0.82
Number of Heavy Atoms (HA)	11	0.72
Molecular Weight (MW)	155.11	
Number of Hydrogen Bond Acceptor (HBA)	4	
Number of Hydrogen Bond Donor (HBD)	2	
MWHBN [MWHBN = (MW^(-0.5)*HBN), where HBN=HBA+HBD]	0.48	0.00
Topological Polar Surface Area(TPSA)	79.53	0.44
pKa	3.7	0.06
**BBB SCORE**		2.46

Table S9. Predicted BBB score of DFP **1**.

DFP **1**		
**Property**	**Value**	**T_0**
Number of Aromatic Rings (Aro_R)	1	0.82
Number of Heavy Atoms (HA)	10	0.65
Molecular Weight (MW)	139.15	
Number of Hydrogen Bond Acceptor (HBA)	2	
Number of Hydrogen Bond Donor (HBD)	1	
MWHBN [MWHBN = (MW^(-0.5)*HBN), where HBN=HBA+HBD]	0.25	0.95
Topological Polar Surface Area(TPSA)	38.95	0.73
pKa	3.68	0.06
**BBB SCORE**		4.37

Table S10. Comparison of predicted BBB scores with percentage neuronal rescue from 6-OHDA neurotoxicity at 100 µM dose of the compound.

**Compound**	**BBB Score**	**% 6-OHDA Rescue (at 100 µM)**
**6a**	3.88	100
**6c**	4.70	89
**6d**	4.05	64
**10a**	4.47	63
**10d**	3.83	76
**11a**	3.97	60
DFP **1**	4.37	117
**2**	3.87	93
**3**	2.46	84

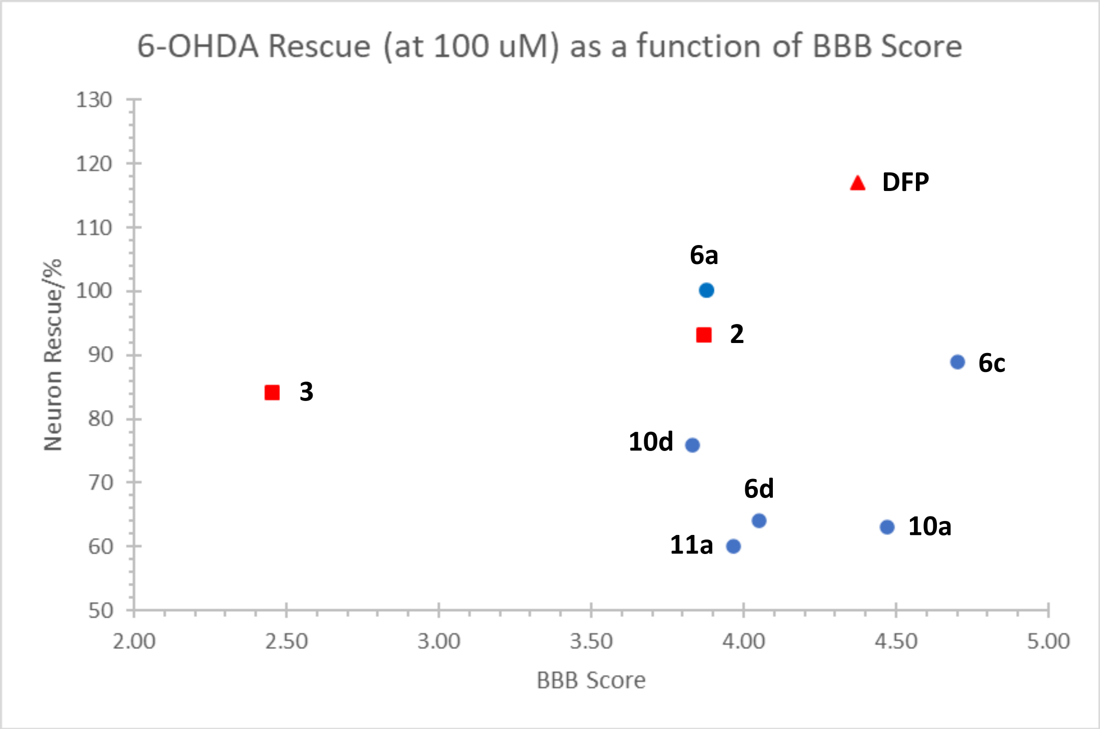

Figure S10. Plot of predicted BBB scores versus percentage neuronal rescue from 6-OHDA neurotoxicity at 100 μM dose of the compound, showing no clear correlation between the two properties (● = **6a**–**6d**, **10a**, **10d** and **11a**, ■ = **2** and **3**, ▲ = DFP **1**).

7. DPPH Antioxidant Assay

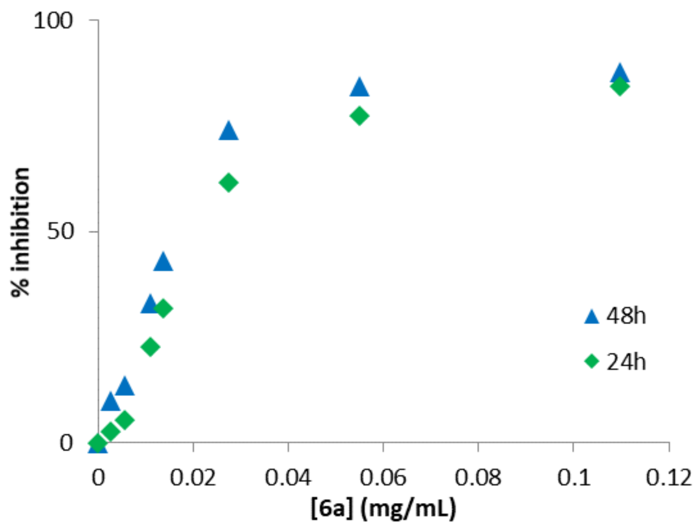

Figure S11. Percentage inhibition of the 2,2-diphenyl-1-picrylhydrazyl (DPPH) radical by ligand **6a** after 24 hours (24h) and 48 hours (48h).

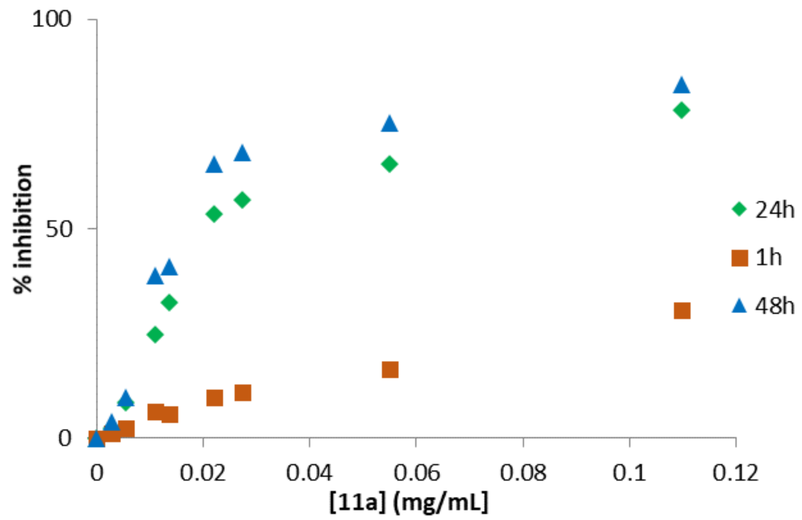

Figure S12. Percentage inhibition of the 2,2-diphenyl-1-picrylhydrazyl (DPPH) radical by ligand **11a** after 1 hour (1h), 24 hours (24h) and 48 hours (48h).

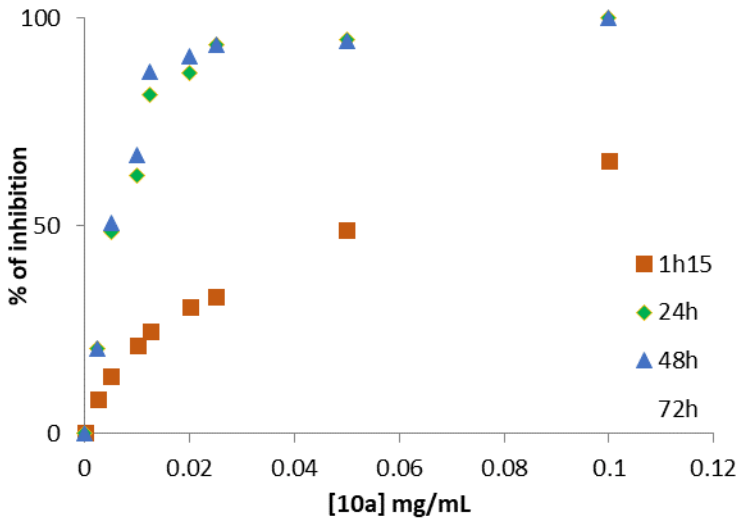

Figure S13. Percentage inhibition of the 2,2-diphenyl-1-picrylhydrazyl (DPPH) radical by ligand **10a** after 1 hour (1h), 24 hours (24h) and 48 hours (48h).

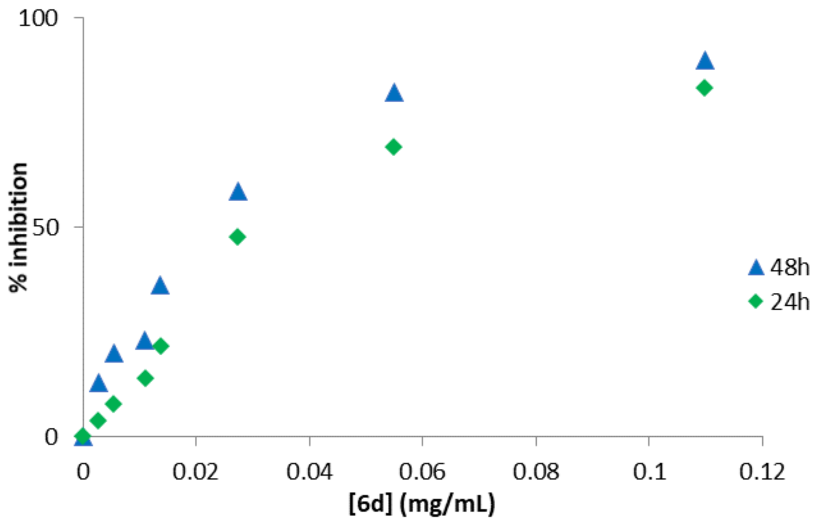

Figure S14. Percentage inhibition of the 2,2-diphenyl-1-picrylhydrazyl (DPPH) radical by ligand **6d** after 24 hours (24h) and 48 hours (48h).

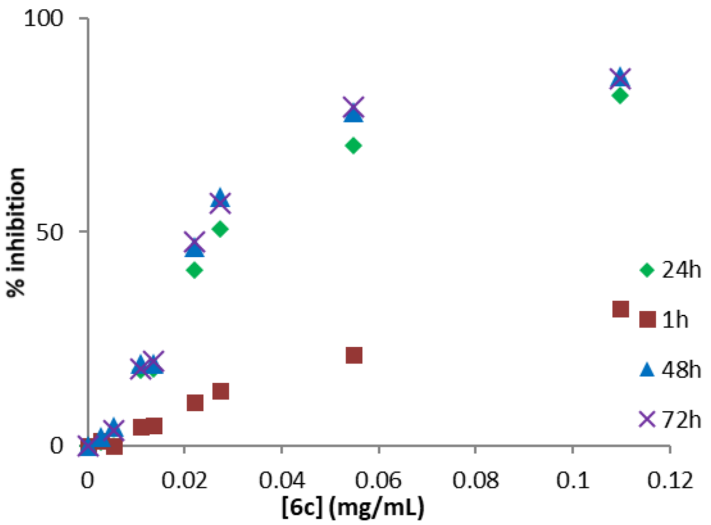

Figure S15. Percentage inhibition of the 2,2-diphenyl-1-picrylhydrazyl (DPPH) radical by ligand **6c** after 1 hour (1h), 24 hours (24h), 48 hours (48h) and 72 hours (72h).

8. Trolox Equivalent Antioxidant Capacity (TEAC) Assay

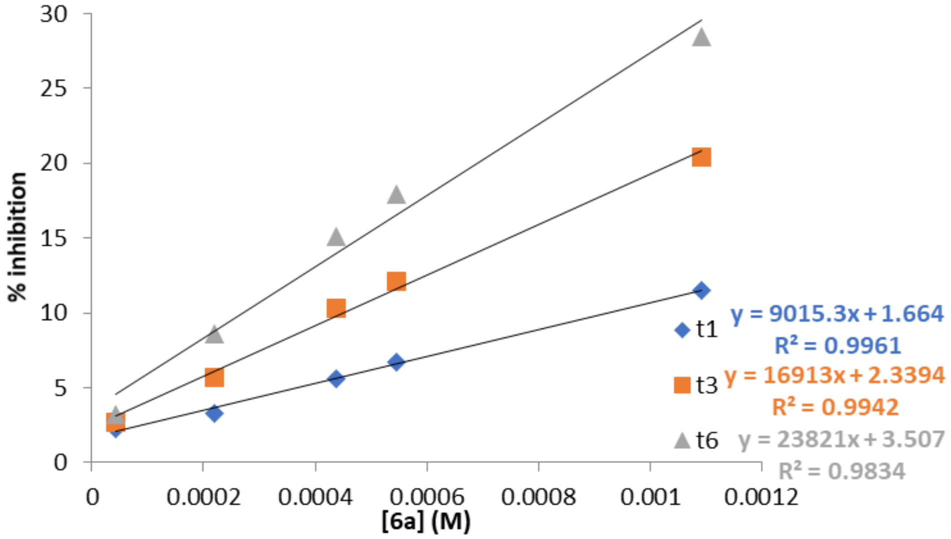

Figure S16. Percentage of ABTS inhibition (TEAC) by ligand **6a** (ABTS = 2,2'-azinobis-(3-ethylbenzothiazoline-6-sulfonic acid)).

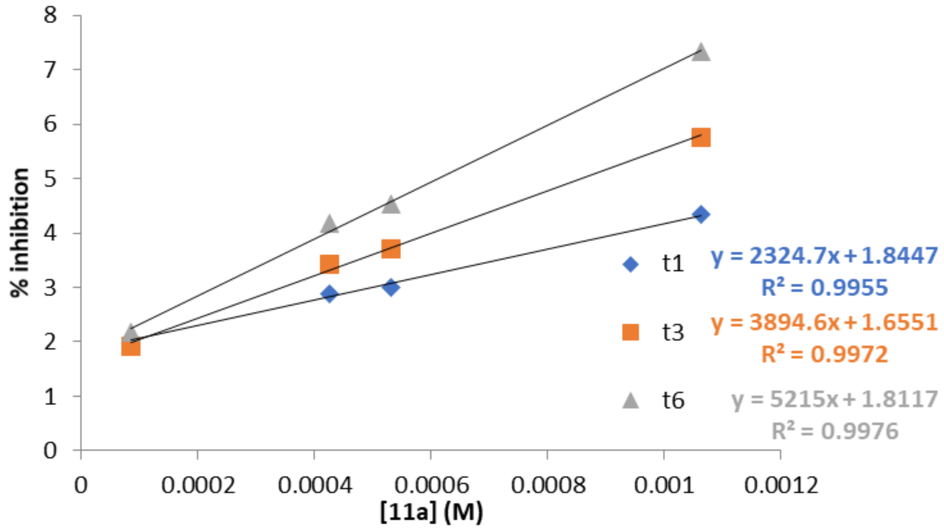

Figure S17. Percentage of ABTS inhibition (TEAC) by ligand **11a** (ABTS = 2,2'-azinobis-(3-ethylbenzothiazoline-6-sulfonic acid)).

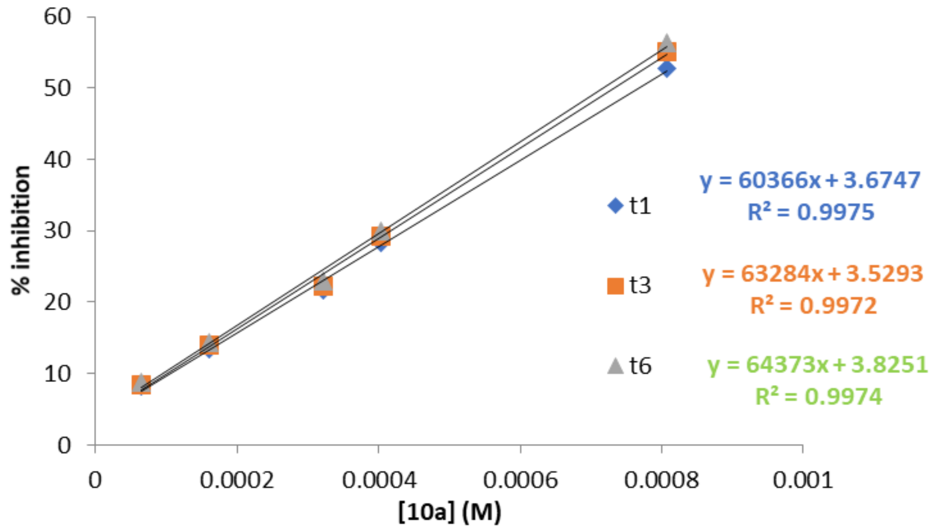

Figure S18. Percentage of ABTS inhibition (TEAC) by ligand **10a** (ABTS = 2,2'-azinobis-(3-ethylbenzothiazoline-6-sulfonic acid)).

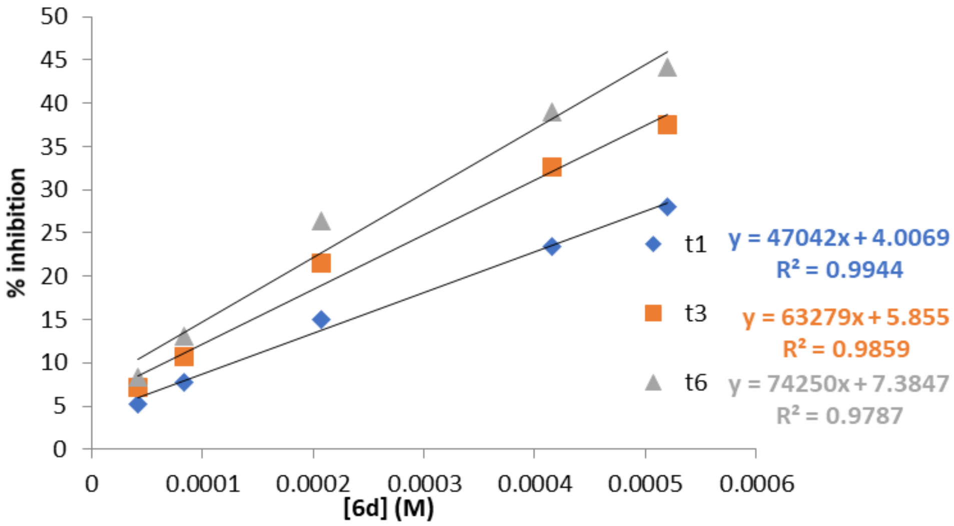

Figure S19. Percentage of ABTS inhibition (TEAC) by ligand **6d** (ABTS = 2,2'-azinobis-(3-ethylbenzothiazoline-6-sulfonic acid)).

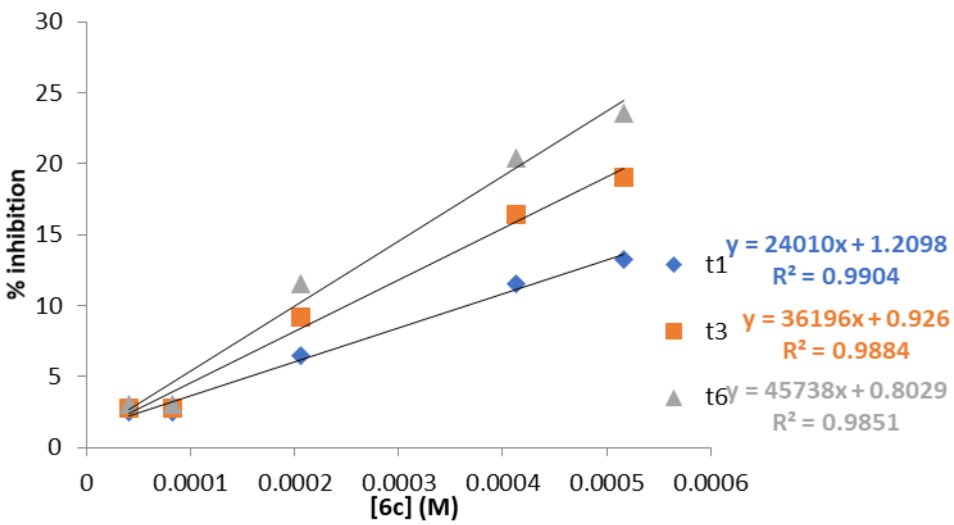

Figure S20. Percentage of ABTS inhibition (TEAC) by ligand **6c** (ABTS = 2,2'-azinobis-(3-ethylbenzothiazoline-6-sulfonic acid)).

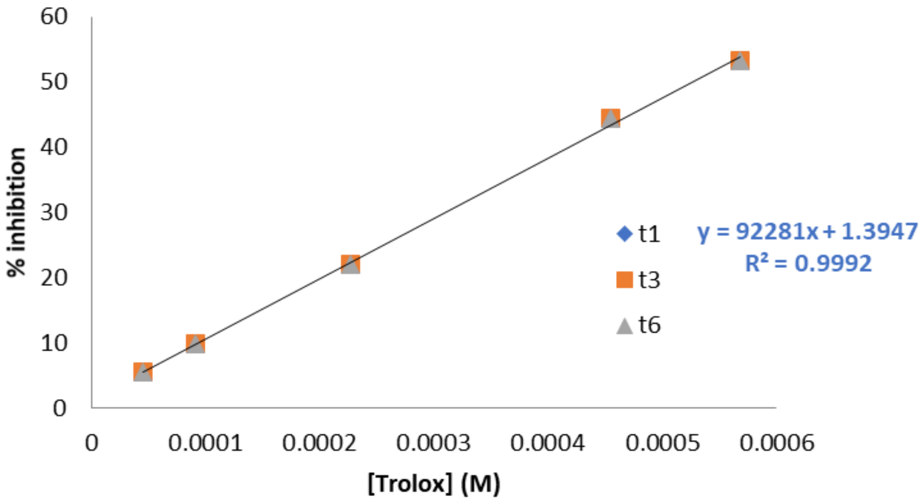

Figure S21. Percentage of ABTS inhibition (TEAC) by Trolox (ABTS = 2,2'-azinobis-(3-ethylbenzothiazoline-6-sulfonic acid)).

9. Neuroprotection against 6-OHDA Neurotoxicity

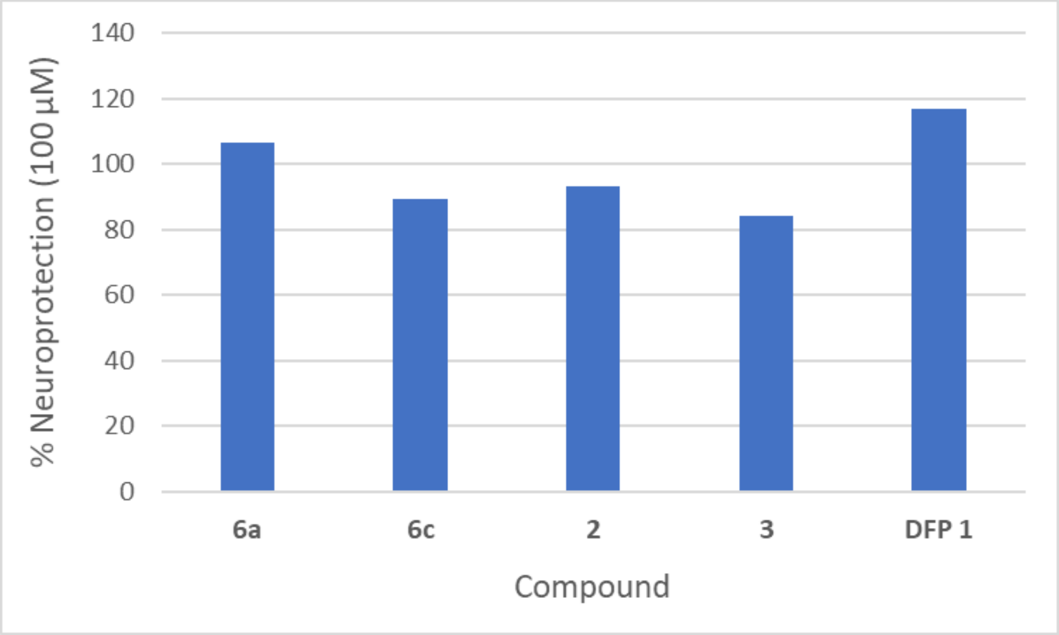

Figure S22. Comparison of the percentage neuroprotection against 6-hydroxydopamine (6-OHDA) neurotoxicity in SH-SY5Y neuroblastoma cells by compounds **6a**, **6c**, **2**, **3** and DFP **1** (at 100 µM compound dose).

10. References

1. (a) S. R. Safir and J. H. Williams, *J. Org. Chem.*, 1952, **17**, 1298–1301; (b) K. Tanaka, K. Matsuo, A. Nakanishi, Y. Kataoka, K. Takase and S. Otsuki, *Chem. Pharm. Bull.*, 1988, **36**, 2323–2330.

2. (a) L. W. Jones and M. C. Sneed, *J. Am. Chem. Soc.*, 1917, **39**, 668–674; (b) K. G. Cunningham, G. T. Newbold, F. S. Spring and J. Stark, *J. Chem. Soc.*, 1949, 2091–2094; (c) A. Cordi, J.-M. Lacoste, V. Audinot and M. Millan, *Bioorg. Med. Chem. Lett.*, 1999, **9**, 1409–1414.

3. A. Katoh, J. Ohkanda, Y. Itoh and K. Mitsuhashi, *Chem. Lett.*, 1992, 2009–2012.

4. F. Gutierrez, C. Tedeschi, L. Maron, J.-P. Daudey, R. Poteau, J. Azema, P. Tisnes and C. Picard, *Dalton Trans.*, 2004, 1334–1347.

5. J. Ohkanda, T. Tokumitsu, K. Mitsuhashi and A. Katoh, *Bull. Chem. Soc. Jpn.*, 1993, **66**, 841–847.

6. (a) M. Frankel, G. Zvilichovsky and Y. Knobler, *J. Chem. Soc.*, 1964, 3931–3940; (b) L. Marchio, N. Marchetti, C. Atzeri, V. Borghesani, M. Remelli and M. Tegoni, *Dalton Trans.*, 2015, **44**, 3237–3250.

7. (a) M. Tegoni, M. Furlotti, M. Tropiano, C.-S. Lim and V. L. Pecoraro, *Inorg. Chem.*, 2010, **49**, 5190–5201; (b) C. M. Zaleski, C.-S. Lim, A. D. Cutland-Van Noord, J. W. Kampf and V. L. Pecoraro, *Inorg. Chem.*, 2011, **50**, 7707–7717; (c) J. Jankolovits, C.-S. Lim, G. Mezei, J. W. Kampf and V. L. Pecoraro, *Inorg. Chem.*, 2012, **51**, 4527–4538.

8. E. E. Smissman and V. D. Warner, *J. Med. Chem.*, 1972, **15**, 681–682.

9. (a) D. Bebbington, N. J. T. Monck, S. Gaur, A. M. Palmer, K. Benwell, V. Harvey, C. S. Malcolm and R. H. P. Porter, *J. Med. Chem.*, 2000, **43**, 2779–2782; (b) D. Bebbington, C. E. Dawson, S. Gaur and J. Spencer, *Bioorg. Med. Chem. Lett.*, 2002, **12**, 3297–3300; (c) H. Schugar, D. E. Green, M. L. Bowen, L. E. Scott, T. Storr, K. Böhmerle, F. Thomas, D. D. Allen, P. R. Lockman, M. Merkel, K. H. Thompson and C. Orvig, *Angew. Chem. Int. Ed.*, 2007, **46**, 1716–1718; (d) D. E. Green, M. L. Bowen, L. E. Scott, T. Storr, M. Merkel, K. Böhmerle, K. H. Thompson, B. O. Patrick, H. J. Schugar and C. Orvig, *Dalton Trans.*, 2010, **39**, 1604–1615.

10. (a) J. Ohkanda and A. Katoh, *J. Org. Chem.*, 1995, **60**, 1583–1589; (b) J. Ohkanda and A. Katoh, *Tetrahedron*, 1995, **51**, 12995–13002; (c) J. Ohkanda and A. Katoh, *Chem. Lett.*, 1996, 423–424.

11. A.-H. Mai, S. Pawar and W. M. De Borggraeve, *Tetrahedron Lett.*, 2014, **55**, 4664–4666.

12. A. Volonterio, P. Bravo and M. Zanda, *Tetrahedron Lett.*, 2001, **42**, 3141–3144.

13. A. Volonterio, S. Bellosta, P. Bravo, M. Canavesi, E. Corradi, S. V. Meille, M. Monetti, N. Moussier and M. Zanda, *Eur. J. Org. Chem.*, 2002, 428–438.
